# Polyoxometalates: more than a phasing tool in protein crystallography

**DOI:** 10.1007/s40828-018-0064-1

**Published:** 2018-08-28

**Authors:** Aleksandar Bijelic, Annette Rompel

**Affiliations:** Universität Wien, Fakultät für Chemie, Institut für Biophysikalische Chemie, Althanstraße 14, 1090 Vienna, Austria

**Keywords:** Polyoxometalates, Hexatungstotellurate, Anderson–Evans structure, Protein crystallization, Crystallization Additive

## Abstract

Protein crystallography is the most widely used method for determining the molecular structure of proteins and obtaining structural information on protein–ligand complexes at the atomic level. As the structure determines the functions and properties of a protein, crystallography is of immense importance for nearly all research fields related to biochemistry. However, protein crystallography suffers from some major drawbacks, whereby the unpredictability of the crystallization process represents the main bottleneck. Crystallization is still more or less a ‘trial and error’ based procedure, and therefore, very time and resource consuming. Many strategies  have been developed in the past decades to improve or enable the crystallization of proteins, whereby the use of so-called additives, which are mostly small molecules that make proteins more amenable to crystallization, is one of the most convenient and successful methods. Most of the commonly used additives are, however, restricted to particular crystallization conditions or groups of proteins. Therefore, a more universal additive addressing a wider range of proteins and being applicable to a broad spectrum of crystallization conditions would represent a significant advance in the field of protein crystallography. In recent years, polyoxometalates (POMs) emerged as a promising group of crystallization additives due to their unique structures and properties. In this regard, the tellurium-centered Anderson–Evans polyoxotungstate [TeW_6_O_24_]^6−^ (TEW) showed its high potential as crystallization additive. In this lecture text, the development of POMs as tools in protein crystallography are discussed with a special focus on the so far most successful cluster TEW.

**Electronic supplementary material:**

The online version of this article (10.1007/s40828-018-0064-1) contains supplementary material, which is available to authorized users.

## X-ray crystallography

**Fig. 1:**
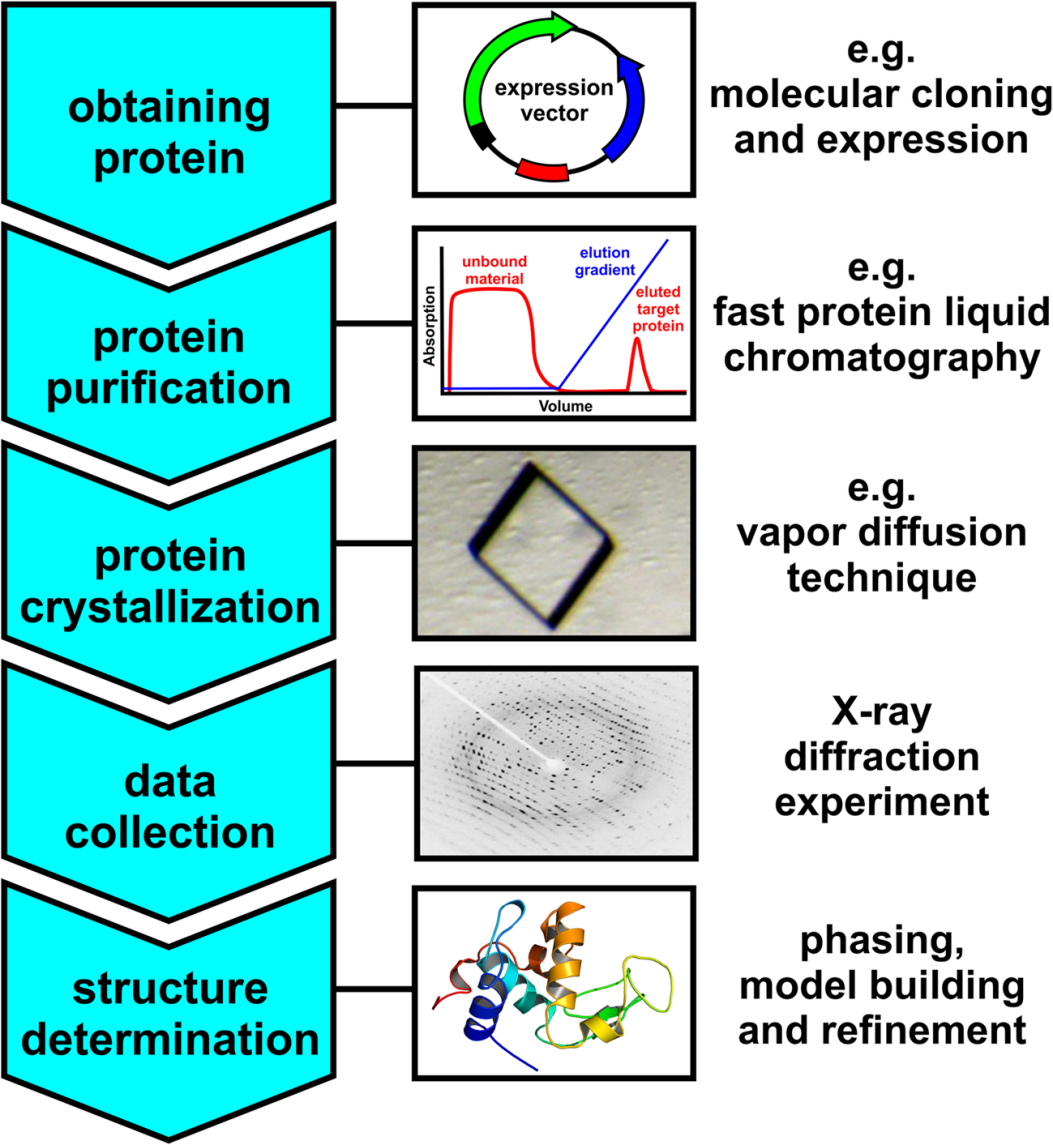
Flowchart representing the major steps during X-ray crystallography. The protein-obtaining process is symbolized by a basic expression vector (molecular biology approach), whereas the subsequent purification process is indicated by an affinity-chromatography chromatogram, where the protein (red sharp peak) is eluted by an elution gradient (blue line). In this way, the protein is separated from contaminants that did not bind to the chromatographic column and consequently flew through the column immediately (broad red peak). The crystallization process is symbolized by a single crystal (hen egg white lysozyme). The following X-ray diffraction experiment is represented by the picture of a diffraction pattern (hen egg white lysozyme), which shows the diffracted reflections as small spots (black spots) over the whole detector area (bright background). The final structure elucidation procedure is symbolized by the 3D crystal structure of a protein (hen egg white lysozyme)

Structural biology is concerned with the molecular structure and dynamics of biological macromolecules, particularly proteins and nucleic acids. The molecular structure of proteins determines their properties and functions, which is of tremendous interest to scientists working in many areas of life sciences as proteins are involved in the most basic processes of life. Furthermore, most of the therapeutically active compounds target proteins and thus structural knowledge is indispensable for revealing relevant drug–protein  interactions to improve existing or design novel drugs. X-ray crystallography is currently the most commonly applied method for macromolecular structure determination as accurate molecular structures can be obtained reliably for very large proteins or even molecular complexes (> 100 kDa) at atomic resolution. This is also reflected in the Protein Data Bank (PDB, http://www.rcsb.org), where ~ 90% of all deposited protein structures were elucidated by X-ray crystallography. The first 3D structure of a protein, which was determined by X-ray crystallography, was that of myoglobin solved by John C. Kendrew (1917–1997) in 1958 [1]. Only 2 years later the structure was joined by that of hemoglobin, which was solved by Max F. Perutz (1914–2002) [2]. Perutz and Kendrew received the Nobel Prize in Chemistry in 1962 for their groundbreaking work. Since then X-ray crystallography has been applied to solve thousands of protein structures leading to the deposition of > 125,000 protein structures in the PDB since 1976 (as of May 2018). The process of X-ray crystallography consists in general of five major steps, namely obtaining sufficient amounts of the target protein, protein purification, crystallization, data collection and structure determination (Fig. 1).

**Fig. 2:**
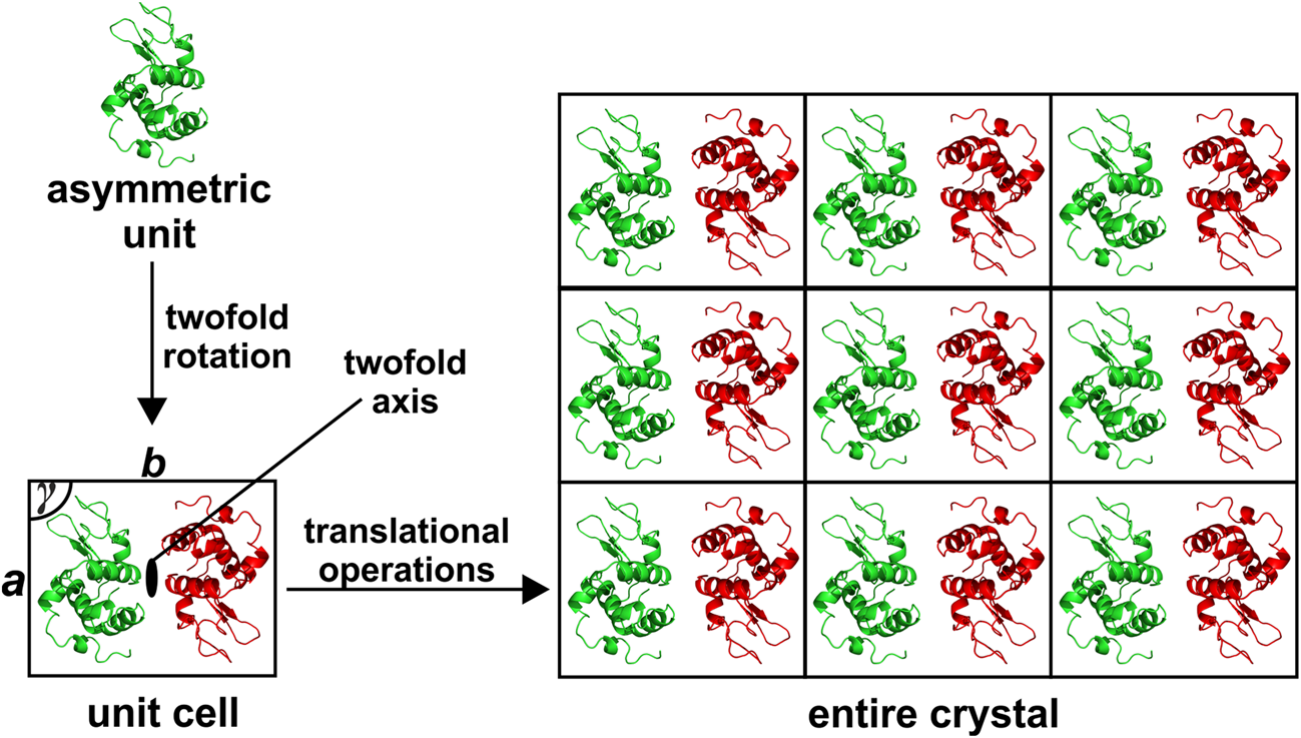
Assembly of a crystal. In this example the asymmetric unit consists of one protein molecule (hen egg white lysozyme, green molecule). The unit cell is generated by applying a rotation of 180° around the twofold axis producing a copy of the molecule (symmetry mate, red molecule). The unit cell describing parameters, lattice constants *a* and *b* and the corresponding angle *γ*, are indicated. Note that for the sake of simplicity, only a 2D crystal is depicted lacking the third dimension. The entire crystal is then built by translationally stacking up unit cells in the 2D space (2D in the figure but 3D in the real crystal)

Depending on the protein of interest, the sample is more or less elaborately obtained. Optimally, the protein of interest is commercially available, which is often the case when investigating the interaction of a (structurally) known protein with different ligands. If the research project requires the structure elucidation of an (structurally) unknown or commercially not available protein, the sample has to be obtained either directly from the natural source or by means of molecular biology. Protein isolation from natural source is accompanied by many difficulties with respect to crystallization [3]. Depending on the nature of the protein and its source organism, the protein of interest might be expressed in small amounts (e.g. as its expression might be associated with certain stimuli), and therefore, a huge amount of the source has to be processed to receive enough protein for crystallization (~ 2–10 mg). Furthermore, the presence of different but structurally highly similar isoforms of the target protein and post-translational modifications (PTMs) lead to inhomogeneous samples hampering the formation of single crystals. Therefore, the molecular biological approach represents the most widely used technique to produce sufficient amounts of the target protein for crystallization. For this purpose, the gene of the target protein is cloned into an expression system and overexpressed in a host cell, which most commonly is an engineered strain of the bacterium *Escherichia coli* (*E. coli*) [4]. Bacterial expression systems are robust and thus usually able to express the target protein in large amounts. Moreover, they ususally do not decorate the target protein with PTMs, which can be either an asset or a drawback. On the one hand the lack of PTMs reduces the inhomogeneity of the sample facilitating crystallization, but on the other hand PTMs are often necessary for the correct folding and function of the protein [5]. When enough protein is produced, the sample is purified to near-homogeneity (removal of proteinogenic and non-proteinogenic contaminants) by different types of chromatography, e.g., hydrophobic interaction, affinity, ion-exchange and size exclusion chromatography. Afterwards, the protein sample is concentrated and subjected to crystallization to obtain single crystals. A protein crystal is a highly ordered array of protein molecules consisting units that repeat throughout the 3D space, which are called unit cells (Fig. 2). These unit cells are spanned by three vectors with defined lengths (*a, b, c*) and angles (*α, β, γ*). Based on the unit cell, the whole crystal can be built up by applying solely translational operations (without any rotation). Unit cells, in turn, are composed of asymmetric units, which are the minimal arrangement that can generate the whole crystal by applying symmetry operations including both translational and rotational symmetry elements (Fig. 2). The grown single crystal is then subjected to the X-ray diffraction experiment. The collected data from the experiment is used to solve the structure of the target protein by mathematical means using sophisticated software ultimately yielding a 3D model of the protein of interest.

## X-ray diffraction experiment

**Fig. 3:**
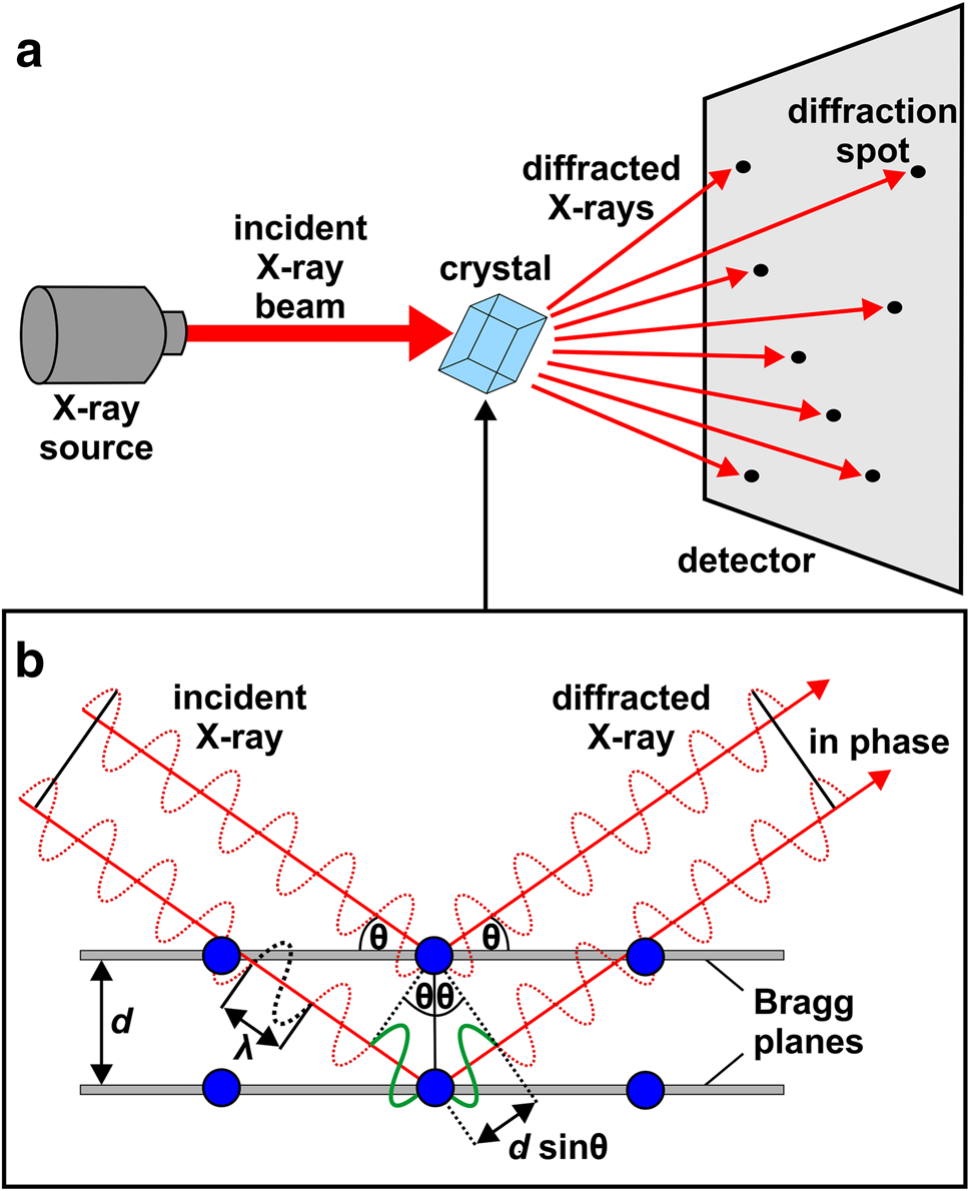
X-ray diffraction experiment. **a** Basic scheme of an X-ray diffraction experiment. An incident X-ray beam enters the crystal and the diffracted rays produce a diffraction pattern (diffraction spots), which are recorded on a detector. **b** Diffraction according to Bragg’s Law. Constructive interference (in phase) is depicted. Constructive interference is only possible, if the path difference (*d* sinθ) between the waves (red dotted waves) is an integer multiple of the wavelength λ, which in this figure is 1 λ (shown as green solid wave). Thus, the amplitudes of the diffracted waves add up producing a measurable signal on the detector. If the path difference is a non-integer multiple of λ, the scattered waves will interfere destructively yielding no observable diffraction. The two grey bars represent two Bragg planes with an interplanar distance spacing of *d*. Each plane contains three lattice points (blue spheres), which in the case of protein crystallography represent atoms of the protein

As an assistance for the readers that are not familiar with X-ray crystallography, the X-ray diffraction experiment will be briefly explained in the following paragraphs. Why do we need X-rays? In all forms of microscopy the resolution, or in other words the amount of detail that can be seen, is determined by the wavelength of the applied electromagnetic radiation. X-rays are high energy electromagnetic waves exhibiting wavelengths in the range of 0.1–100 Å. Thus, they are suitable to ‘see’ the protein in atomic detail since interatomic distances are of that magnitude, e.g., a C–C bond ~ 1.5 Å [6]. During the X-ray diffraction experiment, the X-rays are scattered by electrons of the protein crystal (Fig. 3a). When photons travel through a crystal they interact with electrons and induce oscillation in them leading to the electrons emitting partial waves themselves. These partial waves do mostly interfere destructively, but a few of them superimpose constructively (in phase) in certain directions giving rise to so-called “reflections”, which  are then observed on the detector. The probability of observing diffraction in a certain direction is proportional to the amplitude of the resulting wave (structure factor *F*). This phenomenon of X-ray diffraction by crystals was discovered by Max T. von Laue (1879–1960), for which he was awarded the Nobel Prize for Physics in 1914 [7]. The diffraction experiments of von Laue evidenced both the electromagnetic wave nature of X-rays and the space lattice of crystals. He was the first to mathematically explain the conditions at which diffraction occurs [8]. In 1913, building on the work of von Laue, William L. Bragg (1890–1971) has formulated a simplified interpretation of the conditions that give rise to a constructive interference during the X-ray diffraction experiment, leading to the famous and indispensable Bragg’s Law [9]:

$$n\lambda\,=\,2d\sin \theta .$$

To explain the X-ray diffraction event, Bragg established hypothetical planes (Bragg planes) within the crystal. These planes contain atoms of the crystalline system and are separated by the interplanar distance *d* and are better exemplified by imaginary mirror planes that reflect X-rays (Fig. 3b). According to this, when incoming X-rays of the wavelength *λ* hit equivalent lattice planes at an angle *θ* and are reflected at the same angle, the diffracted waves will interfere constructively (= generating an observable reflection), if and only if the path difference between these X-rays are an integer multiple *n* of the wavelength. For all incident X-rays that do exhibit angles not fulfilling Bragg’s Law the scattered waves will not be in phase (destructive interference) and thus no reflection will be observed at that angle.

## Bottlenecks in macromolecular X-ray crystallography

There is a number of obstacles in macromolecular X-ray crystallography (e.g., obtaining sufficient amounts of the target protein in a pure form), but the major hurdles are the crystallization process itself (= growing single crystals that diffract X-rays to high resolution) and the so-called ‘phase problem’.

## The ‘phase problem’ and its solution

**Fig. 4:**
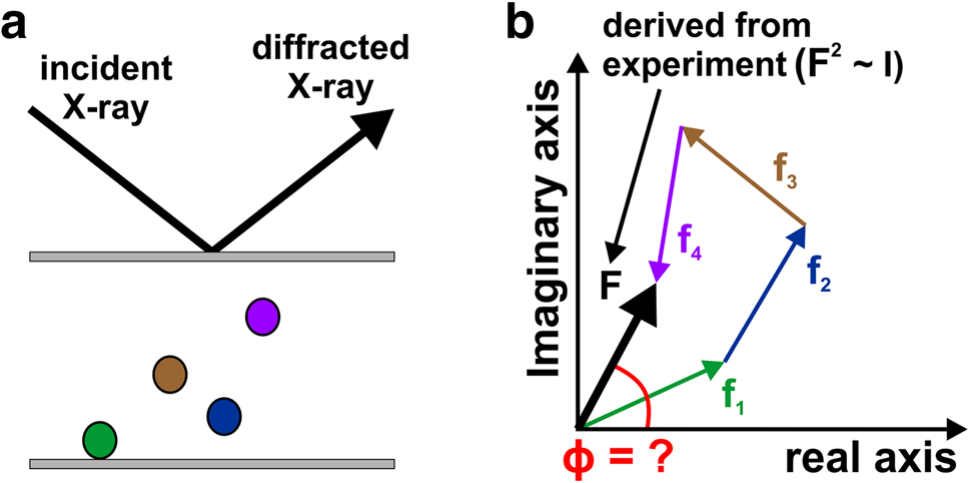
The ‘phase problem’. **a** A scheme of an X-ray diffraction event. Two Bragg planes (grey bars) are shown, together with four atoms (colored spheres) that do not lie on the planes and are thus phase shifted (only atoms located on the planes diffract in phase). Every atom (located on or close to Bragg planes) contributes to every diffracted ray with a relative phase *ϕ*, which is determined by the atom’s relative distance from the planes. **b** The contribution of each atom to the observable (detected) overall reflection (black vector) are depicted as vectors (complex numbers), whereby the vector colors match to those of the atoms shown in a). The figure indicates that the resulting diffraction (*F*) has a particular amplitude (length of the vector) and phase (angle) that results from the summation of all (in this case four) individual scattering factors (*f*_1_–*f*_4_) with each having its own phase and amplitude. The amplitude can be derived from the diffraction experiment as it is proportional to the root of the measured intensity (*I*). Unfortunately, the phase is lost during the experiment (indicated by a red question mark). Therefore, only the electronic properties of the atoms (i.e., diffracting power) can be derived from the experiment but not their structural properties (i.e., phase angle) describing their position within the crystal

Every diffracted X-ray wave that reaches the detector during the X-ray diffraction experiment has a particular amplitude (magnitude of scattered X-rays, see Fig. 3b) and phase angle (*ϕ*, angle describing the relative displacement between waves). To describe the diffraction event in a mathematical way, crystallographers use a quantity that is called the structure factor (*F*), which expresses both the amplitude and the phase of any observed reflection (Fig. 4) [6]. The structure factor itself is a complex number consisting of a real and an imaginary part, and represents a simple summation of all contributing atoms in the unit cell (Fig. 4b). Each atom exhibits its own atomic scattering factor (*f*_*i*_, *i* = atom), which is a measure of the diffracting power of an individual atom and depends on the atom’s identity (specifically, the number of electrons), its movement relative to the incident X-ray, the scattering angle *θ* and to a lesser extent also on the wavelength *λ* of the incident X-ray beam. To solve the protein structure by mathematical means, both the amplitude and the phase of every diffracted ray is needed. The amplitude of the structure factor is provided by the diffraction experiment as the measured intensity (*I*) of the diffracted X-rays is proportional to the square of the amplitude (*F*^2^ ~ *I*). However, the detector is not able to measure the phase angles (*ϕ*) of the reflections, which carry the bulk of the structural information (i.e., positional information). This is referred to as the ‘phase problem’.

There are several methods to recover the ‘lost’ phase information, namely molecular replacement (MR), single or multiple isomorphous replacement (SIR, MIR) and single or multiple wavelength anomalous dispersion (SAD, MAD) [10–12]. MR is the simplest method to solve the ‘phase problem’ as initial phases are obtained from a structurally related protein (as a rule of thumb a sequence identity of ~ 20–30% is required) of which structure (and thus phases) is known and available [13]. The method uses algorithms to find a positioning of the structural homolog (so-called search model) that fits the experimental data. Once the homolog is correctly positioned, its calculated phases are used to phase the unknown structure. The other methods work independently of a homolog but require additional experimental effort and are called experimental phasing. SIR/MIR requires crystals of the native target protein and at least one of a heavy atom derivative. This derivative must be reasonably isomorphous to the native crystal, hence the addition of the heavy atoms must not change the crystal’s cell dimensions compared to that of the native crystal. According to a general rule of thumb, a change in the cell dimensions of *d*_min_/4 is acceptable (*d*_min_ = resolution limit). For example, for a 2.5 Å dataset, a 0.6 Å change in the unit cell (between that of the native and the derivative crystal) might provide valuable phases via isomorphous replacement [14]. However, in percentage terms, unit cell differences as small as ~ 0.5–1.0% can already render isomorphous replacement unsuccessful. The heavy atoms are introduced into the protein structure via co-crystallization (protein and heavy atom solution are mixed prior to crystallization) or soaking (a preformed protein crystal is soaked in a heavy metal containing solution allowing the heavy atoms to diffuse into the protein structure), where they bind to different amino acid side chains. The incorporated heavy atoms contribute strongly to the X-ray diffraction due to their large number of electrons (the scattering contribution of an atom is proportional to the square of the number of its electrons) in comparison to the common light atoms occurring in proteins (carbon, nitrogen and oxygen). As a result, the change in the scattered intensity from the addition of the heavy atoms can be easily determined. These differences in scattered intensities (native crystal vs. heavy atom derivate) do largely reflect the scattering contribution of the heavy atoms and can, therefore, be used to determine the position of the heavy atoms. The heavy atom positions, together with the experimentally measured structure factor amplitudes of the native protein and its heavy atom derivate(s), are used to deduce the phase, e.g., geometrically via Harker construction [12]. Isomorphism between the crystals of the native and heavy atom derivative(s) is indispensable for detecting the intensity differences reliably. To achieve the required isomorphism is, however, very difficult, and therefore, SAD/MAD is more often used nowadays. This technique requires the incorporation of anomalous scatterers into the protein of interest. Anomalous scatterers are atoms that possess an absorption edge within the wavelength range of X-rays that are used for protein crystallography (~ 0.7–2.5 Å) [15]. In this case the differences in scattered intensities originate from the wavelength-dependent change of the scattering factor of the anomalous scatterers. X-ray absorption at (or close to) the absorption edge of the anomalous scatterers leads to a phase shift that differs significantly from that observed during normal elastic scattering. Thus, MAD requires data from only one single crystal but at different wavelengths to exploit the effects of anomalous X-ray dispersion, whereby data is usually collected at three wavelengths. One data set is collected at the peak of the absorption edge (maximal anomalous signal) of the anomalous scatterer, one at the inflection point of the absorption peak and one remote from the peak (high and low dispersion to obtain maximum dispersive differences = maximum wavelength-dependent difference in the scattering factor). In the case of SAD data is only collected at one single wavelength, namely at that of the absorption edge peak. Similar to MIR, the protein phases are derived from the induced differences in the scattering behavior (dispersion differences) between the data sets collected at different wavelengths. It must be ensured that the single crystal, which is used for MAD, is not destroyed during the experiment by radiation as it has to withstand the radiation dosage of multiple measurements (at multiple wavelengths). Therefore, the use of heavy atoms/anomalous scatterers, which are featured prominently in polyoxometalates, presents an  established way of overcoming the ‘phase problem’.

## Protein crystallization

**Fig. 5:**
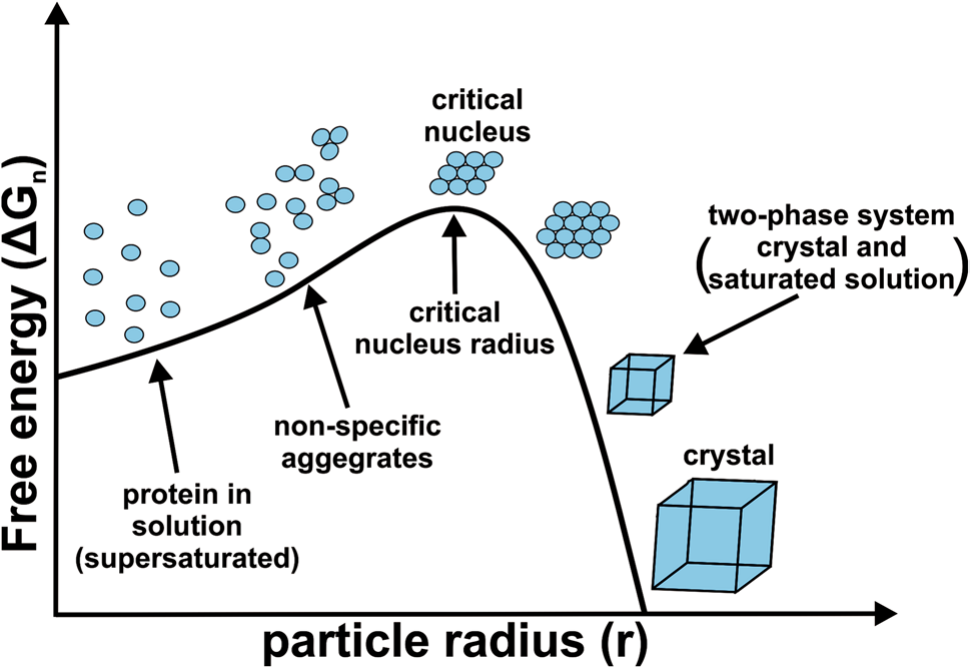
The Gibbs free energy change (Δ*G*_n_) associated with the nucleation of a nucleus with radius *r*. A kinetic energy barrier (peak of the curve) has to be overcome to form a nucleus that is above the critical size to induce the desired phase separation (saturated solution and solid crystal). At a sufficient supersaturation, the protein molecules in solution will collide with each other forming non-specific aggregates. However, some of these aggregates will reach a certain size and more and more protein molecules will attach to their surfaces, ultimately leading to a critical nucleus. Once the size of a nucleus is above the critical size, the gain in binding enthalpy by the absorption of more protein molecules to the nucleus’ surface will compensate the entropic loss during the growth. At this point the system can proceed to yield a single crystal of appropriate size, which is suitable for the X-ray diffraction experiment. Small light blue circles represent protein molecules

Due to the rapid progress of synchrotron facilities (X-ray source) providing X-ray beams of high flux (highly intensive photon beam) and high brilliance (highly collimated photon beam) of which wavelength is tunable (choice of wavelength for MAD) and the further development of phasing programs, the ‘phase problem’ poses only a minor hurdle nowadays. Therefore, obtaining high-quality single crystals represents the rate limiting step in macromolecular crystallography [16]. Protein crystallization is mainly a ‘trial and error’ procedure due to the highly complex physics involved and the dependence on the interplay of multiple factors (e.g., protein and precipitant concentration, pH, temperature, ionic strength, etc.),  leading to a general unpredictability of this process [17]. The reason for this is that crystal formation is an inherently unlikely process as proteins are partially highly flexible and dynamic, which is required for their functionality, and the interactions that hold them together in a crystal (ionic, dipole, van der Waals interactions and hydrogen bonds) are weak, noncovalent and few in number [6]. Furthermore, the few intermolecular contacts between the protein molecules in a crystal are highly specific and depend on the surface characteristics of the protein. This means that the distribution of charged and polar amino acids on the solvent-exposed protein surface determines the crystallizability of a protein. This is also the reason why some proteins are seemingly impossible to crystallize by nature as their surface characteristics are incompatible with crystal lattice formation. But what do we need crystals for? X-ray crystallography depends on the evaluation of structural information produced by the diffraction of X-rays from the protein of interest, which are recorded by detectors. Only ~ 1% of the incident X-ray beam gets diffracted by the protein’s electrons. Therefore, crystals (ordered 3D arrays of protein molecules) are needed to amplify the intensity (signal) of the scattered X-rays as the diffraction from one single protein molecule would not be measurable. To obtain single crystals, a highly purified protein solution needs to be brought to supersaturation, which is required to overcome the kinetic barrier for the formation of crystal nuclei (phase separation) [18]. Nucleation [19] is the most critical step in protein crystallization as it represents the first-order transition from a disordered to an ordered state [20]. A critical nucleus with the necessary size represents the high energy intermediate in the nucleation process. The nucleus at this stage can either fall apart again or continue to grow into a crystal (Fig. 5). The latter event requires a nucleus of larger radius, and therefore, a higher degree of supersaturation.

**Fig. 6:**
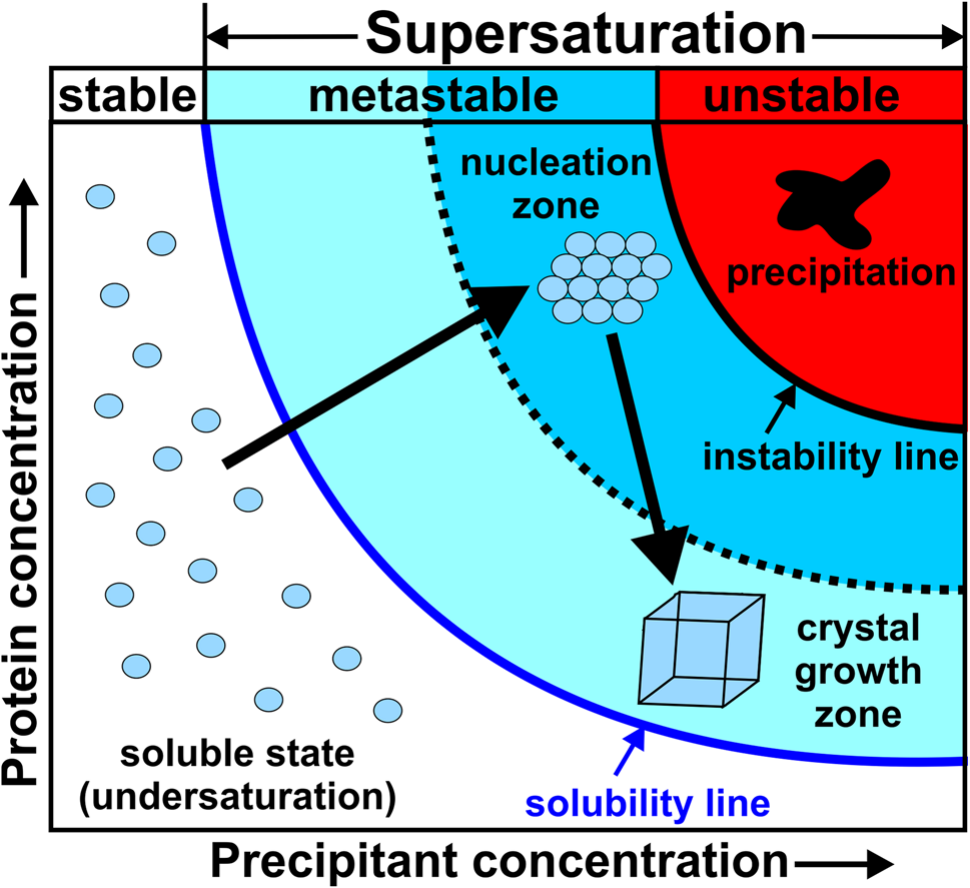
Phase diagram of protein crystallization. The solubility line (dark blue line) divides the diagram into an undersaturated (white background) and supersaturated (light blue, blue and red background) zone. The former zone represents a single phase and stable solution, where the protein molecules (represented as light blue circles) stay in solution. With increasing saturation the solubility line is crossed and the solution becomes supersaturated. The supersaturated region is subdivided into a metastable (light blue and blue background) and unstable (red background) zone. The metastable region consists of a crystal growth (light blue background) and nucleation (blue background) zone. In the crystal growth zone only transient nuclei are formed that do not reach the critical size. If the supersaturation proceeds (indicated by a large black arrow starting from the zone of the soluble state), the nucleation zone is reached, where nuclei can achieve a critical size and become stable (nucleation). As the nuclei become larger and crystals start to appear, the protein concentration in the solution is decreased and the system reaches the crystal growth zone again (indicated by the black arrow pointing downwards into the direction of the crystal growth zone). In this zone crystals continue to grow at lower supersaturation. At too high supersaturation, the unstable zone (red background) is reached, where the proteins precipitate amorphously

Too high protein concentrations promote the disordered aggregation of macromolecules (precipitation), whereas low protein concentrations cannot induce supersaturation. Therefore, it is important to attain an adequate level of supersaturation for nucleation. As a supersaturated protein solution is crowded with protein molecules, they collide frequently with each other and under favorable conditions they collide in orientations that promote the formation of specific protein–protein contacts. In this way, the required nuclei are formed. Accordingly, the nuclei grow as more and more protein molecules are attached to them. This process is associated with the transition of protein molecules from the liquid into the solid-like phase (nuclei) resulting in a decrease in protein concentration in the crystallization solution. The crystallization solution is brought to a thermodynamically metastable state, where the ordered aggregation of the protein molecules is promoted [21]. The required supersaturation is reached by the addition of a precipitant to the protein solution, which in general decreases the solubility of proteins by withdrawing water molecules from their hydration shell making them more likely to associate with each other [22]. Figure 6 shows the phase diagram of a common protein-crystallizing process, where, among others, the nucleation and crystal growth zones are illustrated in dependence of the protein and precipitant concentration. The crystallization process does not only depend on the suitable amount of protein and precipitant but also on other factors like protein purity/homogeneity, pH, temperature, ionic strength, etc. The crystallization diagram combines information about thermodynamically defined phase relations and potential regions of different kinetic processes (nucleation and growth). Crystallization is thermodynamically only possible, if a metastable protein solution reverts towards equilibrium by separating into a stable, protein-rich phase (i.e. crystal) and a saturated growth solution, whereby kinetic processes determine whether the thermodynamically possible occurence of crystals will actually turn into reality [22]. In general, the kinetic events are much harder to control than the thermodynamic conditions, which can be relatively easily adjusted (e.g., by the composition of the crystallization solution, temperature, etc.). Therefore, it is the task of a crystallographer to elaborate the appropriate (thermodynamic and kinetic) conditions, which drives the system towards phase zones that favor crystallization [23].

**Fig. 7:**
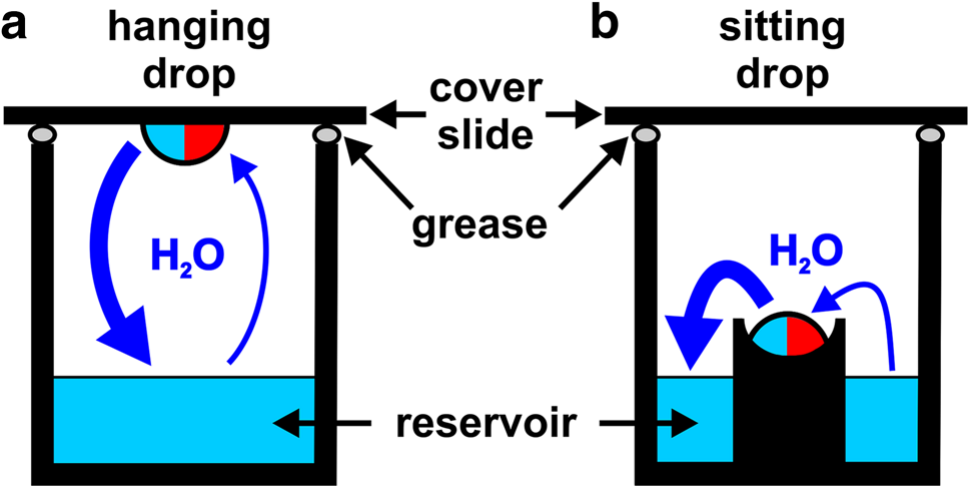
Vapor diffusion crystallization techniques. **a** Hanging drop and **b** sitting drop setup. The wells are covered by slides and sealed by grease. The crystallization drop consists of the crystallization cocktail containing the precipitant (light blue part of the drop) and the protein solution (red part of the drop). Equilibration via vapor diffusion is indicated by dark blue arrows

There are several prominent techniques to obtain single crystals, namely batch crystallization [24], micro-dialysis [25], liquid–liquid free interface diffusion [26] and vapor diffusion [27]. Since the latter technique is the most commonly used one, we will describe only this briefly. The vapor diffusion technique can be performed in two setups, the hanging or sitting drop format (Fig. 7). Both setups share the same mechanism as a protein solution is mixed with a crystallization cocktail solution containing the precipitant yielding the final crystallization drop. In the hanging drop setup a few microliters (usually 0.1–5 µL) of both the protein and crystallization cocktail solution are placed on a siliconized cover slide, which then covers a well containing only the crystallization cocktail (0.05–1 mL), the so-called reservoir (Fig. 7a). In the sitting drop setup the protein and crystallization cocktail solution are mixed within a depression of a small elevated post, which is placed within the crystallization cocktail containing reservoir (Fig. 7b). In both cases the wells are sealed with grease to avoid evaporation and external influences. As the reservoir contains a higher concentration of the precipitant than the crystallization drop (the crystallization drop is diluted by the protein solution), water vapor diffusion from the crystallization drop into the reservoir is triggered to achieve a chemical equilibrium between the crystallization drop and the reservoir. As a result of the vapor diffusion, the crystallization drop shrinks and the concentration of both the protein and precipitant increases slowly until the solubility limit of the protein is exceeded leading to a supersaturated solution. This represents the starting point of the crystallization process, in the course of which nucleation, phase separation and hopefully crystal growth appear (see Fig. 6).

## The use of additives in protein crystallization

Crystallization is a thermodynamically and kinetically highly complex process, which is still not fully understood. The variation of common parameters like protein and/or precipitant concentration, pH, temperature etc. often fail to yield high-quality single crystals. Simple strategies have been developed to improve the crystal quality of initially formed protein crystals or to generally increase the crystallizability of proteins. The use of additives is an established method in protein crystallization to improve or to enable the crystallization of a given protein. The additive is mostly added to the crystallization cocktail (co-crystallization), however, it can also be part of the protein solution. Generally, additives are compounds, mainly small molecules or ions, that have the ability to increase the crystallization probability of a protein by mediating or stabilizing crystal lattice formation. There are two kinds of additives, namely compounds used on a rational basis and compounds with the potential to exhibit features that might affect the crystallization of proteins. The former group includes molecules that affect the protein’s physicochemical properties and/or conformation due to physiological reasons such as substrates, inhibitors, cofactors or other effector molecules. For example, an apoprotein (e.g., an enzyme in absence of its cofactor) might be significantly less prone to crystallization than the respective holoprotein (with bound cofactor) or another ligand-bound form of the protein. Therefore, the use of cofactors and/or ligands can dramatically increase the crystallizability of a given (apo)protein. A reason for this could be that holoproteins are in general less dynamic due to the cofactor/effector-induced structural rigidity. The other group of additives includes molecules/ions that affect the crystallization behavior of proteins in different ways. For example, molecules that affect macromolecule-solvent interactions (similar to the precipitant) like chaotropes and kosmotropes [28]. Molecules that alter the solubility characteristics of macromolecules such as detergents (especially important for membrane proteins) [29] or ions that stabilize certain protein conformations (which are potentially more prone to crystallization) can also have beneficial effects on the crystallization outcome [30]. The most promising molecules of this group are those which are able to provide reversible ‘crosslinks’ within the protein (intramolecular) or between different protein molecules (intermolecular) via hydrogen bonds, electrostatic or hydrophobic interactions (since these ‘crosslinks’ are not of a covalent nature, the term is marked by inverted commas in this review) [31]. These kind of interactions not only stabilize the crystal lattice but can also lead to new crystal contacts (protein–protein contacts). Multivalent and charged ions or molecules are usually used as additives with “crosslinking” properties. The use of most of these additives is, however, limited to certain proteins and/or particular crystallization conditions rendering them futile for the crystallization of many proteins. Therefore, there is still a need for new additives that are able to promote the crystallization of a wide range of proteins, including specific ‘uncrystallizable’ proteins, and tolerate a broad spectrum of crystallization conditions. In this regard, the group of polyoxometalates (POMs) [32] could represent promising prospects as additives in protein crystallization. Due to their unique structures and properties like high solubility, thermal stability and charge, POMs seem to be ideal for the interaction with proteins in a crystallization-promoting manner.

## Polyoxometalates

**Fig. 8:**
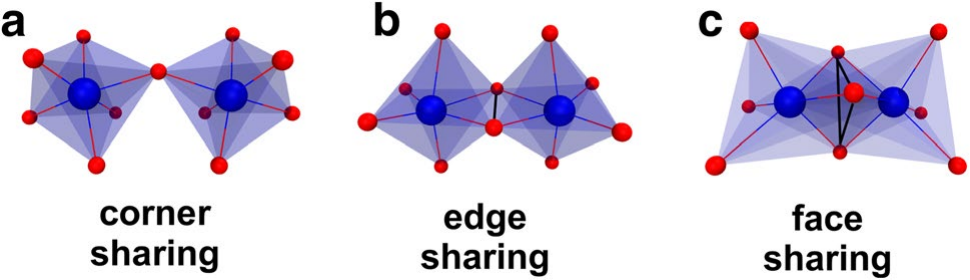
Common coordination modes of octahedral {MO_6_} units. **a** Corner sharing, **b** edge sharing and **c** face sharing mode are illustrated. The octahedra are depicted transparently for the sake of clarity. The edge and face area of the edge and face sharing mode, respectively, are highlighted in black. Color code: dark blue, M; red, oxygen

Polyoxometalates (POMs) are a class of inorganic clusters composed of oxygen and early transition metal atoms (Mo, W, V, Nb, Ta), usually in their highest oxidation states. They exhibit an overwhelming diversity in size and structure with outstanding properties and functions [32]. POMs have been studied vigorously leading to their application in many fields like catalysis [33], nanoscience [34], medicine [35–40] and recently macromolecular crystallography [41, 42]. POMs comprise isopolyanions and heteropolyanions exhibiting the general formula [M_*m*_O_*y*_]^*n*−^ and [X_*x*_M_*m*_O_*y*_]^*n*−^, respectively. M is the so-called addenda atom or polyatom (early transition metal ion), mostly Mo^6+^, W^6+^ or V^5+^. X is the heteroatom, which is either a main group or also a transition metal. While there is no restriction in the choice of the heteroatom X, the addenda atoms are restricted to transition metals possessing a favorable charge/radius ratio and empty d-orbitals to form M–O bonds with oxygen atoms via *dπ*–*pπ* overlapping (electron transfers from filled p-orbitals of the oxygen atoms to empty d-orbitals of the addenda metals). POMs are composed of {MO_*y*_} units (*y* = 4–7) with *y* representing the coordination number of the addenda atom M. These {MO_*y*_} units, with the distorted octahedral {MO_6_} unit being the most common building block, are packed together (self-assembly) in various ways to yield POMs exhibiting different shapes and sizes. This self-assembly occurs mainly via edge and/or corner sharing of the {MO_*y*_} units and only in the rarest cases via face sharing. The reason for this is that in the former coordination modes the  Coulomb repulsions between the addenda metal ions  are significantly reduced due to an increased metal–metal distance (Fig. 8).

Isopolyanions, are in general synthesized by the acidification of a solution containing the addenda metal oxoanion via Brønsted acid-base condensation–addition reactions. Heteropolyanions are obtained in the same way but in the presence of a heteroatom in the form of an oxo/hydroxoanion [42–44]. Examples for the synthesis of both POM types are shown in the following with phosphorus being the heteroatom in the heteropolyanion case:

$${\text{Isopolyanions}}:~7{\text{WO}}_{4}^{{2 - }}+{\text{ }}8{\text{ }}{{\text{H}}^+} \to {\text{ }}{\left[ {{{\text{W}}_7}{{\text{O}}_{24}}} \right]^{6 - }}+{\text{ }}4{\text{ }}{{\text{H}}_2}{\text{O}}$$

$$\begin{aligned} {\text{Heteropolyanions}}:~12{\text{MoO}}_{4}^{{2 - }} ~ & + {\text{PO}}_{4}^{{3 - }} + {\text{ }}24{\text{ H}}^{ + } \to {\text{ }}\left[ {{\text{PMo}}_{{12}} {\text{O}}_{{40}} } \right]^{{3 - }} \\ & + {\text{ }}12{\text{ H}}_{2} {\text{O.}} \\ \end{aligned}$$

Note that these equations are oversimplifications as the exact mechanism of POM formation, especially the driving force for the self-assembly process, is still more than elusive. The self-assembly of POMs depends on a series of factors like pH, concentration of the constituents, temperature, reaction media, counter ions, etc. [32].

**Fig. 9:**
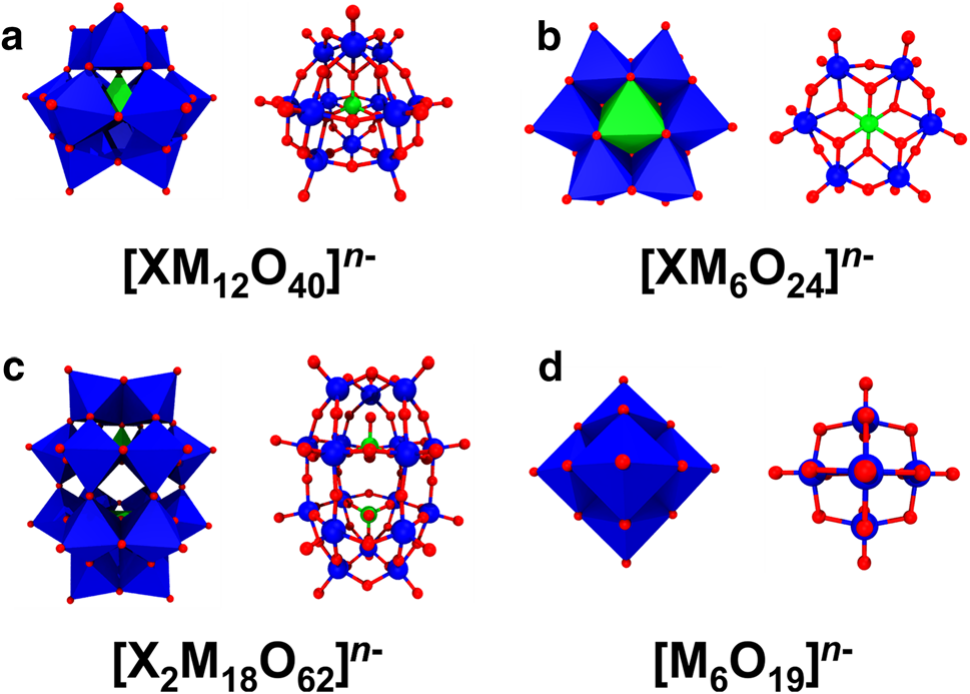
Overview of the most prominent POM structures. **a** Keggin, **b** Anderson–Evans, **c** Wells–Dawson and **d** Lindqvist structure. The structures are shown in both octahedra (left) and ball and stick (right) mode. Color code: dark blue, M; green, X; red, oxygen

More than 200 years ago, the group of POMs was already anticipated and investigated by some of the most famous scientists like Carl W. Scheele (1742–1786), Jöns J. Berzelius (1779–1848) and Linus C. Pauling (1901–1994). In 1826, Berzelius published the very first POM-related report describing the synthesis of a POM, which is now known as (NH_4_)_3_[PMo_12_O_40_] [45]. However, it still took 85 more years until the structure of this first POM was determined by James F. Keggin (1905–1993), a doctorate student of the (already mentioned) famous Nobel laureate (1915 in Physics) and founder of molecular crystallography William L. Bragg [46]. In the following years Keggin contributed greatly to the understanding of POM structures and their self-assembly by the packing of {MO_6_} units, and therefore, in tribute to him POMs exhibiting the general formula [XM_12_O_40_]^*n*−^ (= [XO_4_{(MO_6/2_)_3_}_4_]^*n*−^) are called Keggin structures (Fig. 9a).

**Fig. 10:**
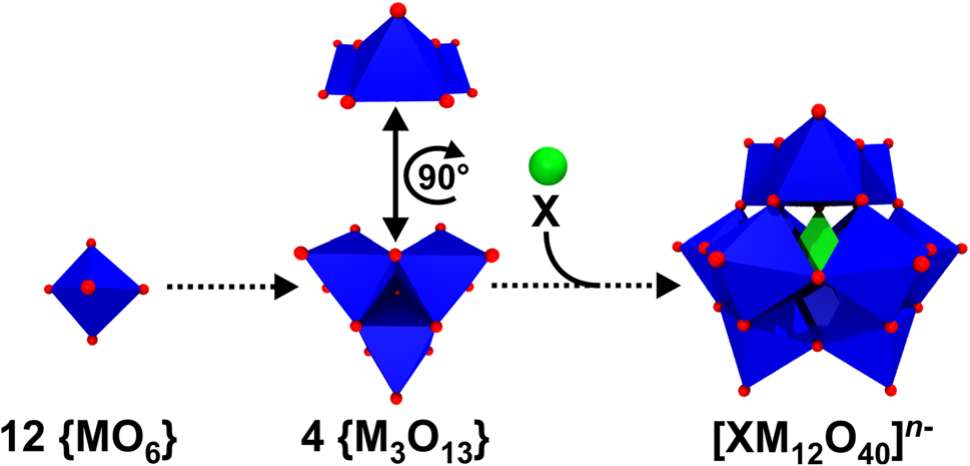
Structural assembly of the Keggin anion. In the middle the {M_3_O_13_} unit is shown from two perspectives to clarify that the ‘cap’ and the ‘belt’ of the Keggin structure are build up by the same {M_3_O_13_} unit. Color code: dark blue, M; X, green; red, oxygen

**Fig. 11:**
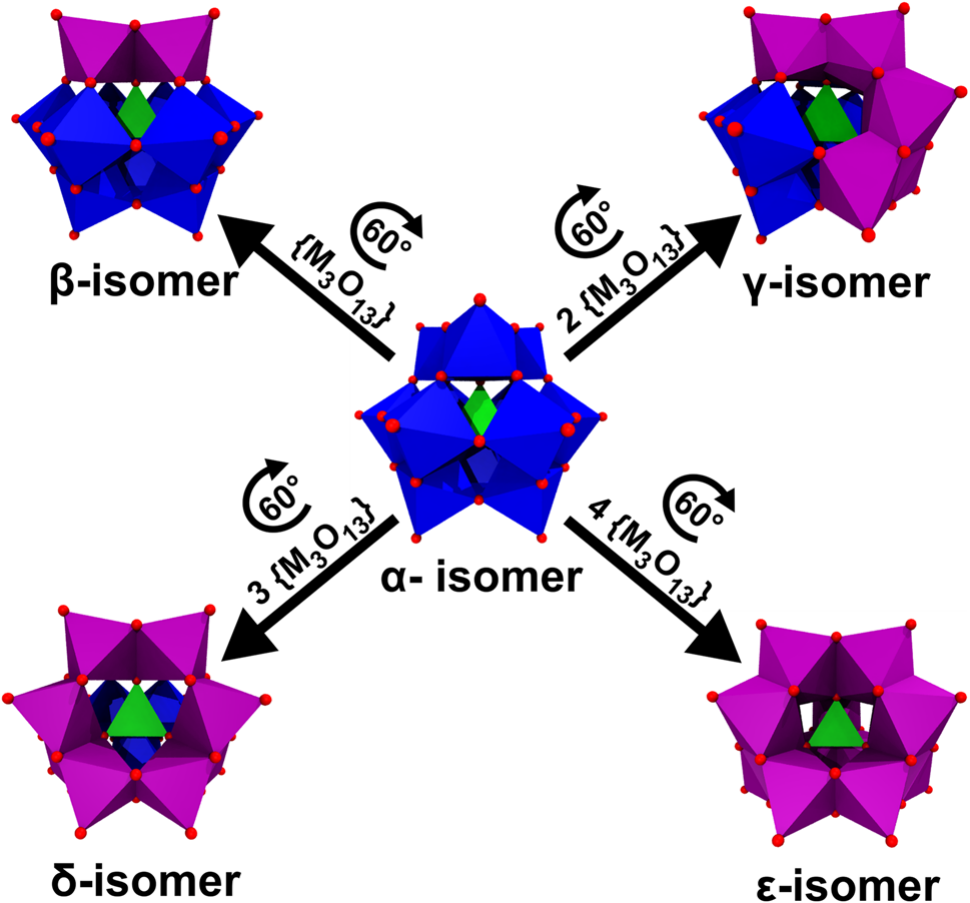
Polyhedral structures of all Keggin isomers. Purple {M_3_O_13_} units represent the triads, which have been rotated by 60° with respect to the α-isomer to obtain the next isomer. Color code: dark blue/purple, M; green, X; red, oxygen

The Keggin structure (α-isomer) exhibits tetrahedral symmetry (*T*_d_) and is composed of 12 octahedral {MO_6_} units, which are arranged in such a way that three edge-shared {MO_6_} units form larger {M_3_O_13_} units with four of the latter building up the structure via corner sharing (Fig. 10). Depending on the rotational orientation of these {M_3_O_13_} units, the Keggin structure can exhibit up to five different isomers, namely the *α*-, *β*-, γ-, *δ*- or *ε*-isomer (Fig. 11).

**Fig. 12:**
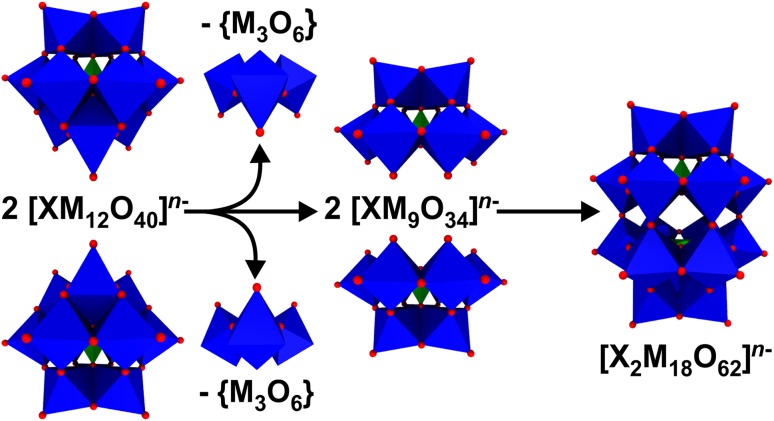
Structural assembly of the Wells–Dawson anion. Please note that the addenda atom M of the {M_3_O_6_} fragment is not octahedrally coordinated as oxygen atoms are missing to complete the octahedral arrangement. The {M_3_O_6_} fragment is depicted in octahedra mode only for clarity (to better understand which parts are missing in the lacunary units). For more information, please see the text. Color code: dark blue, M; green, X; red, oxygen

In 1937, John S. Anderson (1908–1990) predicted the structure of some 6-heteropolyacids like that of the hexamolybdoperiodate [I(Mo_6_O_24_)]^5−^ [47, 48]. A decade later, Anderson’s assumption was structurally confirmed by the crystal structures of the hexamolybdotellurates *Z*_6_[TeMo_6_O_24_] (*Z* = ${\text{NH}}_{4}^{+}$ or K^+^), which were crystallized by Howard T. Evans Jr. (1920–2000). Therefore, POMs of the general type [XM_6_O_24_]^n−^ (= [XO_6_(WO_6/2_)_6_]^n−^) are referred to as Anderson–Evans structures (Fig. 9b) [48, 49]. The Anderson–Evans structure, which exhibits a trigonal symmetry (*D*_3h_), is composed of a central octahedrally arranged {XO_6_} heteroatom that is surrounded by a planar arrangement of six edge sharing {MO_6_} units. About the same time (1939–1953) Alexander F. Wells (1912–1994) and Barrie Dawson (1925–1974) were engaged in the structure elucidation of other POMs like that of 18-heteropolyoxotungstates during which Wells suggested the structure of the trigonal anion [P_2_W_18_O_62_]^6−^ [50]. 14 years later, the structure was crystallographically confirmed by his colleague Dawson, which is why POM structures of the formula [X_2_M_18_O_62_]^*n*−^ (= [(XO_4_)_2_(MO_6/2_)_18_]^*n*−^) are called Wells–Dawson structures nowadays (Fig. 9c) [51]. The Wells–Dawson polyanion (α-isomer) exhibits a trigonal symmetry (*D*_3h_) and is formed by the fusion of two [XM_9_O_34_]^*n*−^ building blocks via corner-sharing. The [XM_9_O_34_]^*n*−^ building block represents a Keggin anion that lacks one {M_3_O_6_} unit and is, therefore, referred to as trilacunary Keggin structure (Fig. 12). In 1952, the renowed POM-chemist  Ingvar Lindqvist (1921–1991) provided the first ever X-ray structural analysis of a paratungstate. The proposed structure [W_12_O_46_]^20−^ with 12 {WO_6_} octahedra was based exclusively on the determination of the positions of the tungsten atoms [52]. During the following years many unsuccessful attempts were undertaken to confirm the assumed correctness of Lindqvist’s structural proposal. William N. Lipscomb Jr. (1919–2011) was the first who noticed that no comparable POM contains more than two terminal oxygen atoms per {WO_6_} octahedron, one of Lindqvist’s assumptions. By not changing the tungsten positions, the future Nobel Prize winner in Chemistry (1976) proposed a more compact structure, namely [W_12_O_42_]^12−^ and as protonated form [H_2_W_12_O_42_]^10−^ (= [H_2_(WO_6/1.895_)_6_(WO_6/1.565_)_6_]^10−^) [53]. Direct evidence was obtained by ^1^H broad-line NMR spectroscopy of lithium, sodium, and potassium paratungstate indicating  that the two non-acidic protons are located in the polyanion’s center and are separated by 2.22(2) Å [54]. One year later Lipscomb’s structural proposal was confirmed by the X-ray structural analysis of ammonium paratungstate, (NH_4_)_10_[H_2_W_12_O_42_][55]. Later on, the location of the two protons could be exactly determined by neutron diffraction of ammonium paratungstate [56]. Besides proposing the structure of paratungstate, Lindqvist crystallographically solved the structures of a series of other POMs, among them that of hexaniobate [Nb_6_O_19_]^8-^ (= [(NbO_6/1.89_)_6_]^8-^) [57]. Therefore, POMs exhibiting the general formula [M_6_O_19_]^*n*−^ (= [(MO_6/1.89_)_6_]^*n*−^) are named Lindqvist structures in his honor (Fig. 9d). The Lindqvist structure is an isopolyanion (no heteroatom), which is composed of an octahedral arrangement (O_h_ symmetry) of six {MO_6_} units, wherein each unit is sharing four edges with adjacent units. Today there are hundreds of POM or POM-based structures, the  five most common structures (Keggin, Anderson, Wells-Dawson, paratungstate and Lindqvist) have been discussed.

## Polyoxometalates in protein crystallography

The introduction of POMs into the field of protein crystallography was based on a mixture of rational reasoning and serendipitous discovery. As POMs represent clusters of heavy metals (Mo, W, V, Nb and Ta), they are ideal for SIR/MIR-based phasing to overcome the ‘phase problem’. Some POMs like polyoxotungstates (POTs) are particularly powerful in this regard as their addenda tungsten atoms do not only possess a large amount of electrons but do also act as anomalous scatterers. Since the *L*–*I* absorption edge of tungsten is positioned at 1.02 Å, it provides significant anomalous signals at the wavelength usually used for the X-ray diffraction experiment (~ 1 Å). Thus, the more commonly used SAD/MAD method can be applied to solve the ‘phase problem’. Due to this feature, POMs were most frequently used as phasing tools [58]. The analysis of all POM-containing protein crystal structures deposited in the PDB and the associated research papers revealed that the presence of the respective POM was rather a coincidence, despite the clusters having partially great impact on the crystallization process of the protein [41]. The POMs were largely not used in a targeted manner by the researchers as they were not added as preformed molecules to the crystallization approach with the purpose to induce a crystallization-enhancing effect. In most cases, the POMs were formed by accident in the course of the crystallization experiment as the crystallization conditions contained diverse transition metal oxoanions (e.g., ${\text{MO}}_{4}^{{n - }}$ with M = Mo^6+^ or V^5+^), which then self-assembled into POMs. There are different reasons to introduce transition metal oxoanions into protein crystallization set-ups, among others, they can act as inhibitors (e.g., ${\text{VO}}_{4}^{{3 - }}$ for bacterial Acid Phosphatase A [59]) or substrate analogs (e.g., VO_3_^−^ as phosphate analog in human cell cycle protein CksHs1 [60]) for various enzymes and are thus primarily used as additives. In this regard, vanadate is often used as a phosphate mimetic or inhibitor of phosphate depending or converting proteins like phosphatases due to its chemical similarity with phosphate and the ability to form penta-coordinated complexes, whose coordination geometry represents a good approximation to the transition state of phosphoryl transfer reactions [61, 62]. Nevertheless, POMs were also intentionally used as additives in protein crystallization exhibiting beneficial effects on the crystallization process. Tables 1 and 2 provide a brief overview of POM containing crystal structures that have been deposited in the PDB indicating which POMs were used intentionally and for what reason (Table 1) and which POMs were formed accidentally during the crystallization trial (Table 2). To induce crystallization promoting effects, it is (largely) indispensable that the POM is able to directly interact with the biomacromolecules, therefore, POM–protein interactions are briefly discussed in the following section.

**Table 1:** Overview of POM containing PDB entries with the POM being intentionally used in the respective study (as of May 2018)

PDB entry	Protein (organism)	POM	Purpose of POM use	Effect on crystallization	References
1DV4	Small ribosomal subunit (*T. thermophilus*)	[P_2_W_18_O_62_]^6−^	Phasing	Structure stabilization by rigidification	[63]
1FKA	Small ribosomal subunit (*T. thermophilus*)	[P_2_W_18_O_62_]^6−^	Phasing	Structure stabilization by rigidification	[64]
1I94^a^	Small ribosomal subunit (*T. thermophilus*)	[P_2_W_18_O_62_]^6−^	Phasing	Structure stabilization by rigidification	[65]
1N7D	LDL receptor (human)	[PW_12_O_40_]^3−^	Phasing	Stabilization of domain packing and increase in crystal quality	[66]
2G8H	DNA repair protein Rad51 (*M. voltae*)	[H_2_W_12_O_40_]^6−^	Inhibitor	Stabilization of the inactive form	[67]
3ZX0	NTPDase1 (*R. norvegicus*)	[Mo_7_O_24_]^6−^	Inhibitor	Structure stabilization	[68]
3XZ2	NTPDase1 (*R. norvegicus*)	[V_10_O_28_]^6−^	Inhibitor	Structure stabilization	[68]
4BVO	NTPDase 1 (*L. pneumophila*)	[H_2_W_12_O_40_]^6−^	Inhibitor	Mediating crystal contacts and structure stabilization by rigidification	[69]
4BVP	NTPDase 1 (*L. pneumophila*)	[Mo_8_O_28_]^8−^	Inhibitor	Structure stabilization by rigidification	[69]
4BVP	NTPDase 1 (*L. pneumophila*)	[Mo_7_O_24_]^6−^	Inhibitor	Structure stabilization by rigidification	[69]
4PE5	NMDA receptor ion channel (*R. norvegicus*)	[H_2_W_12_O_40_]^6−^	Phasing	Increase in crystal quality	[70]
4OUA	Tyrosinase (*A. bisporus*)	[TeW_6_O_24_]^6−^	Additive	Mediating crystal contacts	[71]
4PHI	Lysozyme (*G. gallus*)	[TeW_6_O_24_]^6−^	Additive	Mediating crystal contacts (new crystal form)	[72]
4Z12	Aurone synthase (*C. grandiflora*)	[TeW_6_O_24_]^6−^	Additive	Mediating crystal contacts and increase in crystal quality	[73]
4XYY	Lysozyme (*G. gallus*)	[Zr(PW_11_O_39_)]^3−^	Artificial protease^b^	None	[74]
5FHW	Lysozyme (*G. gallus*)	[Hf(P_2_W_17_ O_61_)]^6−^	Artificial protease^b^	None	- [c]
5WP6	TRPM4 channel (human)	[V_10_O_28_]^6−^	Channel modulator	Structure was solved by electron microscopy	[75]
5SUQ	Sub2 ATPase–THO complex (*S. cerevisiae*)	[PW_12_O_40_]^3−^	Phasing	No effect described	[76]
6G3S	HSP70 Nucleotide binding domain (human)	[TeW_6_O_24_]^6−^	Additive	Introduction of uncommon crystal packing	[77]

^a^PDB entries 1I95, 1I96 and 1I97 contain the same POM exhibiting the same effect and are thus omitted in this table

^b^The POM exhibits proteolytic activity on the protein and the crystal structure was used to prove the POM’s binding site

^c^There is yet no research paper associated with this PDB entry

**Table 2:** Overview of POM containing PDB entries with the POM being accidently formed during the respective study (as of May 2018)

PDB entry	Protein (organism)	POM	Origin of POM	POM effect on crystallization/structure	References
1DKT	Cell cycle protein CksHs1(human)	[V_7_O_19_]^3−*^	Self-assembly in presence of ${\text{VO}}_{4}^{{3 - }}$ (phosphate analog)	Stabilization of the protein’s dimer	[60]
1E59	Cofactor-dependent phosphoglycerate mutase (*E. coli*)	[V_4_O_13_]^6−^	Self-assembly in presence of ${\text{VO}}_{4}^{{3 - }}$ (inhibitor)	Stabilization of the inactive form	[78]
1L7V	ABC transporter (*E. coli*)	[V_4_O_12_]^4−^	Self-assembly in presence of ${\text{VO}}_{4}^{{3 - }}$ (inhibitor)	POM was used for phasing	[79]
1UZI	C3 exoenzyme (*C. botulinum*)	[V_4_O_12_]^4−^	Self-assembly in presence of ${\text{VO}}_{4}^{{3 - }}$	Mediating crystal contacts	[80]
1RXS	Uridine phosphorylase (*E. coli*)	[V_7_O_19_]^3−*^	Self-assembly in presence of ${\text{VO}}_{4}^{{3 - }}$	None	[81]
2D1G	Acid Phosphatase A (*F. tularensis*)	[V_10_O_28_]^6−^	Self-assembly in presence of ${\text{VO}}_{4}^{{3 - }}$ (inhibitor)	Structure stabilization by rigidification	[59]
2G8H	RNase H (*B. halodurans*)	[V_6_O_19_]^8−*^	Self-assembly in presence of ${\text{VO}}_{4}^{{3 - }}$ (substrate mimic)	Stabilization of an intermediate conformation	[82]
2HHL	CTD small phosphatase-like protein (human)	[PW_12_O_40_]^3−^	Not described	Mediating crystal contacts	[83]
1P0Z	Sensor kinase CitA (*K. pneumonia*)	[Mo_7_O_24_]^6−^	Self-assembly in the presence of ${\text{MoO}}_{4}^{{2 - }}$ (inhibitor)	Mediating crystal contacts	[84]
2OGX	Molybdenum storage protein (*A. vinelandii*)	[W_3_O_13_]^8−*^	Protein induced assembly in the presence of ${\text{WO}}_{4}^{{2 - }}$	None^a^	[85]
4F6T	Molybdenum storage protein (*A. vinelandii*)	[Mo_8_O_26_H_n_]^n−5*^	Protein induced assembly in the presence of ${\text{MoO}}_{4}^{{2 - }}$	None^a^	[86]
4F6T	Molybdenum storage protein (*A. vinelandii*)	[Mo_8_O_28_]^8−*^	Protein induced assembly in the presence of ${\text{MoO}}_{4}^{{2 - }}$	None^a^	[86]
4F6T	Molybdenum storage protein (*A. vinelandii*)	[Mo_6_O_27_H_n_]^n−18*^	Protein induced assembly in the presence of ${\text{MoO}}_{4}^{{2 - }}$	None^a^	[86]
4BRH	NTPDase 1 (*L. pneumophila*)	[V_10_O_28_]^6−^	Self-assembly in presence of ${\text{VO}}_{4}^{{3 - }}$ (phosphate mimic)	None^a^	[87]
4NDO^[b]^	Molybdenum storage protein (*A. vinelandii*)	[Mo_3_O_13_]^8−*^	Protein induced assembly in the presence of ${\text{MoO}}_{4}^{{{{2-}}}}$	None^a^	[88]
4NDO^[b]^	Molybdenum storage protein (*A. vinelandii*)	[Mo_8_O_28_]^8−^	Protein induced assembly in the presence of ${\text{MoO}}_{4}^{{2 - }}$	None^a^	[88]
5O5W	Molybdenum storage protein (*A. vinelandii*)	[Mo_8_O_28_]^8−^	Protein induced assembly in the presence of ${\text{MoO}}_{4}^{{2 - }}$	None^a^	[89]
3GQI	Receptor tyrosine kinase (human)	[V_10_O_28_]^6−^	Self-assembly in presence of ${\text{VO}}_{4}^{{3 - }}$ (inhibitor)	Structure stabilization	[90]
4B1A	Lysozyme (*G. gallus*)	[PMo_12_O_40_]^3−^	Self-assembly upon decomposition of a Mo-complex drug	Structure stabilization	[91]
5XLS	Uracil:protein symporter (*E. coli*)	[PW_12_O_40_]^3−^	Self-assembly in presence of (NH_4_)_2_WS_4_	POM was used for phasing	[92]

*This POM cluster was never isolated from solution

^a^The molybdenum storage protein exhibits a quaternary structure that is reminiscent of a barrel and within this barrel the protein stores both Mo and W atoms in form of POMs, therefore, the POM assembly is protein induced due to physiological reasons

^b^PDB entries 4NDP, 4NDQ and 4NDR contain the same POM exhibiting the same effect and are thus omitted in this table

## POM–protein interactions

**Fig. 13:**
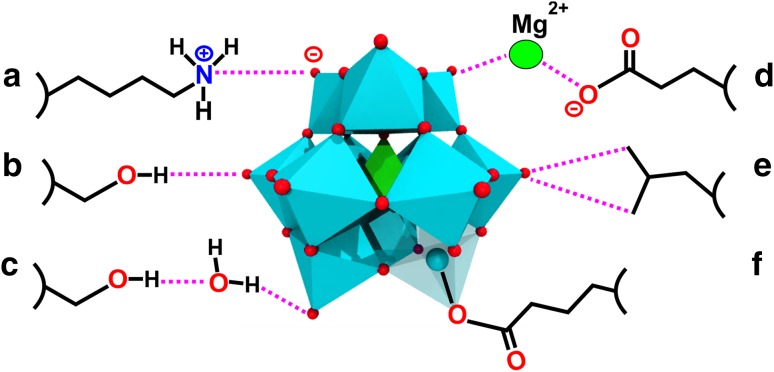
Schematic overview of POM–protein interactions. **a** Electrostatic charge–charge interaction between the POM and a lysine, **b** hydrogen bonding between the POM and a serine, **c** solvent (water) mediated H-bonding between the POM and a serine, **d** cation (Mg^2+^) mediated electrostatic interaction connecting two negatively charged groups (POM and aspartate), **e** van der Waals or hydrophobic interactions between the POM and a leucine and **f** covalent bond between the POM and a glutamic acid. The {MO_6_} octahedron covalently binding the glutamic acid is displayed transparently with the addenda atom M being depicted as a sphere to illustrate the covalent bond in more detail. Color code: cyan, M; green octahedron, X; green sphere, magnesium; red, oxygen

Due to their negative charge, POMs are predestined to interact with positively charged protein regions via charge–charge or, in more general terms, electrostatic interactions (Fig. 13a). Electrostatic interactions as the main driving force for POM–protein interactions were experimentally proven by several studies investigating the interaction of several POMs with human serum albumin (HSA) [92–97] and of course by X-ray crystallography revealing that mainly positively charged amino acids (lysine, arginine and histidine) are involved in these interactions [41, 98]. Furthermore, POMs can also interact with proton-donating amino acids (serine, threonine, cysteine, tyrosine, asparagine and glutamine) via hydrogen bonding (Fig. 13b). Based on this, POMs are able to interact with polar solvent molecules (e.g., water) and/or mono- and multivalent cations (e.g., Mg^2+^). In this way, POMs can ‘indirectly’ interact with the protein via solvent or cation-mediated interactions. This means that a solvent molecule or cation bridges both the POM and the protein by interacting simultaneously with both groups via hydrogen bonds or electrostatic interactions, respectively (Fig. 13c, d). The cation-mediated protein interactions enable the POM to interact also with negatively charged amino acids (glutamic acid and aspartic acid), which usually is impeded by electrostatic repulsion forces (Fig. 13d) [85, 86]. POM–protein interactions were also observed at hydrophobic regions of proteins, for example, different polyoxomolybdates (POMos) were found to predominantly interact with hydrophobic amino acids (e.g., valine, proline and glycine) of the bacterial molybdenum storage protein via van der Waals interactions (Fig. 13e) [88]. This could be explained by the high polarizability of POMs, which seems to enhance the permanent dipole–induced dipole interactions with more or less hydrophobic protein regions. The ability of POMs to exhibit hydrophobic interactions was further confirmed by the finding that POMs have a strong affinity towards neutral and hydrophobic surfaces (interaction with nonionic surfactants and membrane lipids) [95–102]. POMs can, depending on their charge density, environment (e.g., counter-cation) and reaction partner, switch between electrostatic and hydrophobic-like interactions. For example, POMs exhibiting a lower charge density show an increased tendency for hydrophobic interactions, which enhances their affinity towards membrane mimetics. Some POMs are even able to interact with membrane mimetics bearing negatively charged headgroups [102]. In these cases the negative charge of the headgroups is largely neutralized by the POM’s counter-cations enabling the adsorption of the POM to the alkyl region of the membrane. Interestingly, also covalent bonds between POMs and protein amino acid side chains were observed (Fig. 13f) [73, 85, 86]. Some octamolybdates [Mo_8_O_26_]^4−^ were covalently bound to the N_ε2_ nitrogen atom of histidine and the O_*ε*1_ oxygen atom of glutamic acid within the structure of the bacterial molybdenum storage protein. It has to be noted that the formation of the observed POMos was induced by the protein since its primary function is the storage of Mo and W atoms in the form of POMs. Thus, the covalent bonds might arise from the protein-driven assembly process. Similarly, during the crystallization of the bacterial nucleoside triphosphate diphosphohydrolase 1 (NTPDase1), octamolybdate was covalently bound by the hydroxyl oxygen of a serine [69]. The POMo was not added as an intact molecule to the experiment but was formed during the crystallization process as the crystallizing solution contained ${\text{MoO}}_{4}^{{{\text{2-}}}}$ ions. The only case, where a preformed POM was used as a crystallization additive and formed a covalent bond with the protein was during the crystallization of the Anderson–Evans anion [TeW_6_O_24_]^6−^ with the plant aurone synthase from *Coreopsis grandiflora* (*Cg*AUS1) [73, 103]. The POM did covalently bind to a glutamic acid confirming the capability of POMs to form covalent bonds with proteins by a yet unknown mechanism. Covalent binding of POMs can have a great impact on the crystallization process as we will see later.

## POMs as phasing tools

As already indicated, POMs were primarily used for solving the ‘phase problem’. Especially POTs are a particular good choice for phasing as their numerous tungsten atoms are electron-dense anomalous scatterers rendering them extra suitable for SAD/MAD phasing. A certain number of heavy atoms/anomalous scatterers per amino acid residue are needed in the protein crystal to generate isomorphous/anomalous signals that are measurable against the background (noise). This is especially important for the phasing of large proteins or macromolecular assemblies as with increasing size (residue number) more heavy atoms/anomalous scatterers are required [104]. In this regard, POMs as large dense metal clusters have a decisive advantage over commonly used single heavy atoms (e.g., Hg^2+^, Au^3+^ or Pt^2+/4+^) as a significantly lower number of POMs has to be incorporated in the protein structure, in comparison to the number of single heavy atoms, to provide usable phasing signals [105]. The binding of one single POT introduces already a multiple number of heavy atoms. To directly calculate experimental phases at high resolutions, the position of each individual heavy atom/anomalous scatterer within the POT has to be localized and the POT has to be correctly orientated. This can become quite challenging, if the quality of the POT-derivatized crystals is low. However, metal clusters like POTs provide also useful phasing signals at low resolutions, where their individual metal atom sites cannot be resolved. In this case, the individual metal atoms (within the POT) scatter in phase and act as a ‘superatom’ [58]. The total scattering contribution of this ‘superatom’ is composed of the sum of all (anomalous) electrons within the POT providing significantly higher phasing signals than randomly distributed single heavy atoms/anomalous scatterers [58]. A POT consisting of N metal atoms and *Z* electrons will provide, at low resolution, a scattering contribution of (N*Z*)^2^ as the intensity of scattered X-rays is proportional to the square of the number of scattering electrons. In contrast, individual heavy atoms/anomalous scatterers exhibit a signal contribution of N(*Z*)^2^, which is lower by a factor of N than that contribution of the ‘superatom’ [58]. In this way, very strong isomorphous or anomalous signals are obtained from POT-derivatized crystals and the centers of mass of the POTs are accurately detected for phasing. Another aspect that makes some POMs especially suitable for phasing, in comparison to most of the commonly used phasing tools (e.g., single metal ions, small metal compounds, halides), is that they are stable in aqueous solution. Crystallization solutions often contain various inorganic and organic molecules such as additives, buffer, stabilizers and/or precipitants that could interfere chemically with the phasing agent and thus limit its functionality. For example, some buffers like TRIS (tris(hydroxymethyl)aminomethane), phosphate or citrate are known to interfere with the binding of some heavy metals by the formation of partially insoluble complexes (e.g., lanthanides and phosphate) [14]. In contrast, some POMs, especially the Anderson–Evans anion, are stable at a wide range of conditions and inert towards most of the usual constituents of crystallization solutions [42].

Early on, various POTs like the Keggin anion [PW_12_O_40_]^3−^ and the Wells–Dawson structure [P_2_W_18_O_62_]^6−^ were successfully used for the phasing of some large proteins and molecular assemblies, for example, bacterial riboflavin synthase [106], bacterial fumarase C [107], the archaeon 20S proteasome core complex [108], bacterial ribosomal 50S subunit [109], yeast RNA polymerase II [110] and the human low-density lipoprotein receptor [111]. Therefore, Jena Bioscience (http://www.jenabioscience.com) offers ‘Phasing Kits’ that include the Keggin anion [PW_12_O_40_]^3−^, metatungstate [H_2_W_12_O_40_]^6−^ and paratungstate [H_2_W_12_O_42_]^10−^ as derivatization agents.

### Polyoxomoletalates as crystallization agents

**Fig. 14:**
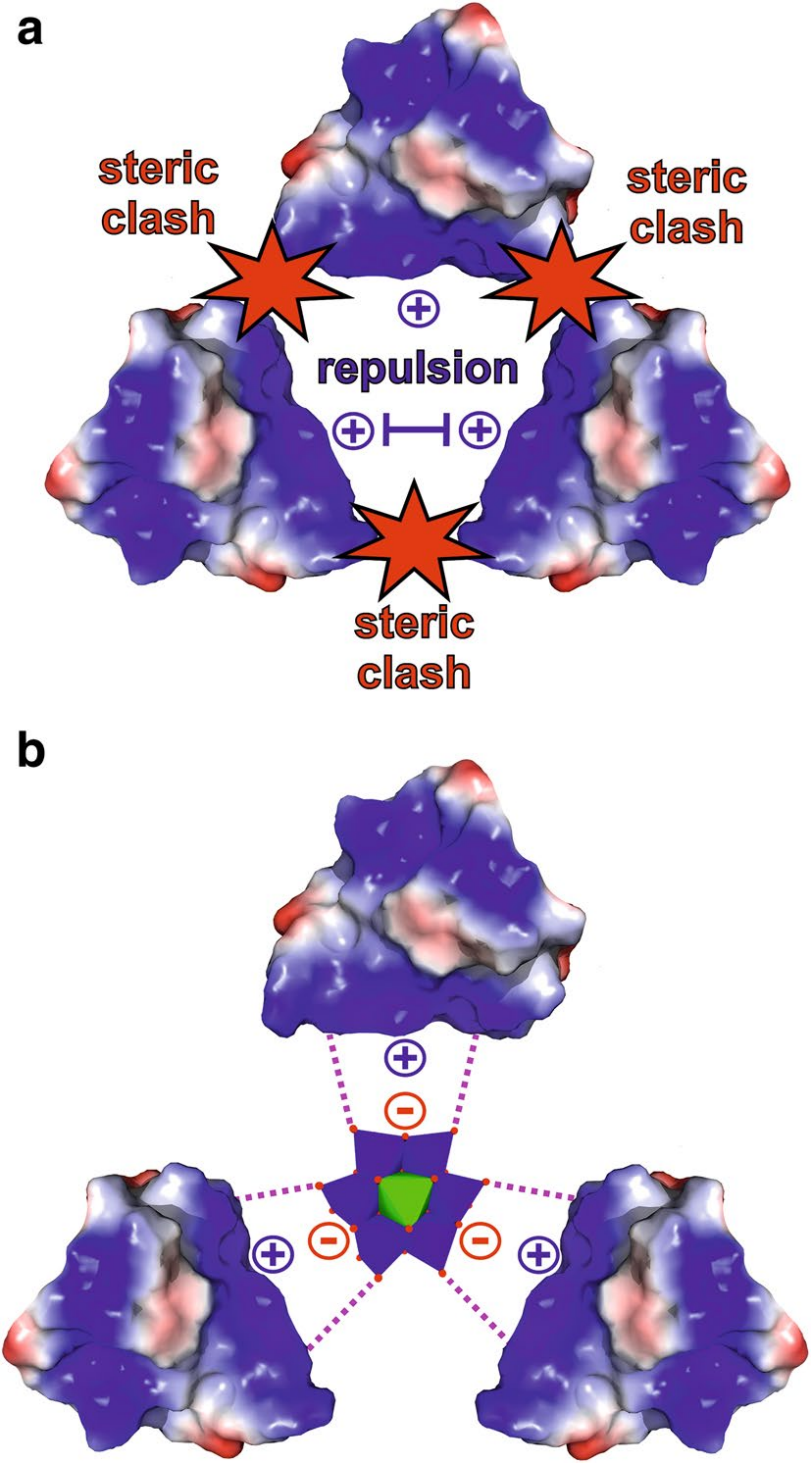
Schematic illustration of the ‘spacer effect’ of POMs exemplified by the Anderson–Evans POM. **a** Scenario, where three positively charged protein patches (shown as electrostatic Coulombic surfaces with blue area = positive potential, red area = negative potential and white area = neutral potential) are approaching each other but (crystal) contact formation is prevented by steric clashes (red stars) and/or electrostatic repulsion. **b** Anderson–Evans POM-mediated crystal contact formation. In the presence of the POM, the equally charged molecules are electrostatically ‘crosslinked’, while still exhibiting appropriate distances between each other preventing steric interference. Color code: dark blue, M; green, X; red, oxygen

Besides their successful use as phasing tools, POMs have also been shown to exhibit beneficial effects on the crystallization process of a series of proteins [41]. The main driving force of POM-mediated crystallization enhancement is their ability to induce and stabilize crystal contacts. Due to the mainly electrostatic interactions (including hydrogen bonding) with positively charged and/or polar patches on the protein’s surface, POMs are able to ‘crosslink’ different protein molecules leading to new protein–protein contacts. Especially the ‘crosslinking’ of positively charged protein surfaces via charge–charge interaction between the POM and the protein molecules is of great benefit as these surfaces would otherwise repel each other due to electrostatic repulsion. ‘Gluing’ these (equally charged) protein patches together via POMs enhances the number of possible crystal contacts, which in turn increases the protein’s crystallization probability. Furthermore, POMs are also able to stabilize the oligomeric state of both biological and crystallographic oligomers (e.g., dimer) and known crystal contacts of proteins, which are formed independently of the POM. The ability of POMs to induce and stabilize the connection between protein molecules in a crystallization enhancing manner depends on several factors like charge, charge density, size, shape and symmetry. Charge/charge density and size/shape mainly determine the POM’s binding affinity to proteins. As the charge density determines, among others, the hydrophilicity of the POM, it has to be chosen appropriately to ensure sufficient electrostatic interactions with the protein. POMs bearing a too high charge density are generally more hydrated and tend to stay in the bulk solvent due to the high solvation/desolvation energy, which is necessary to remove the solvation shell of the POM for protein interaction. POMs exhibiting a very low charge density exhibit weak and promiscuous electrostatic interactions [95, 112, 113]. The size and shape have also a decisive role in the POM’s binding power and ‘crosslinking’ degree. Larger POMs provide more potential interaction sites enabling the interaction with a higher number of amino acids and protein molecules, whereas small POMs are rather limited in this regard. The generally large size of POMs allows them to function as a spacer between ‘crosslinked’ protein molecules (Fig. 14). This spacing can be very important as potential long-range repulsion forces or steric interferences between the bridged proteins are reduced. As a result, the spatial approach of the protein molecules is facilitated and the short-range attraction between the otherwise electrostatically repelling surfaces is stabilized by the POM, which is of particular importance for the nucleation process. Thus, a balance between the POM’s size/shape and charge is decisive to obtain optimal POM–protein interactions, which not only affects electrostatic interactions like charge–charge and hydrogen bonding but also hydrophobic interactions as discussed in the “POM–protein interactions” section.

**Fig. 15:**
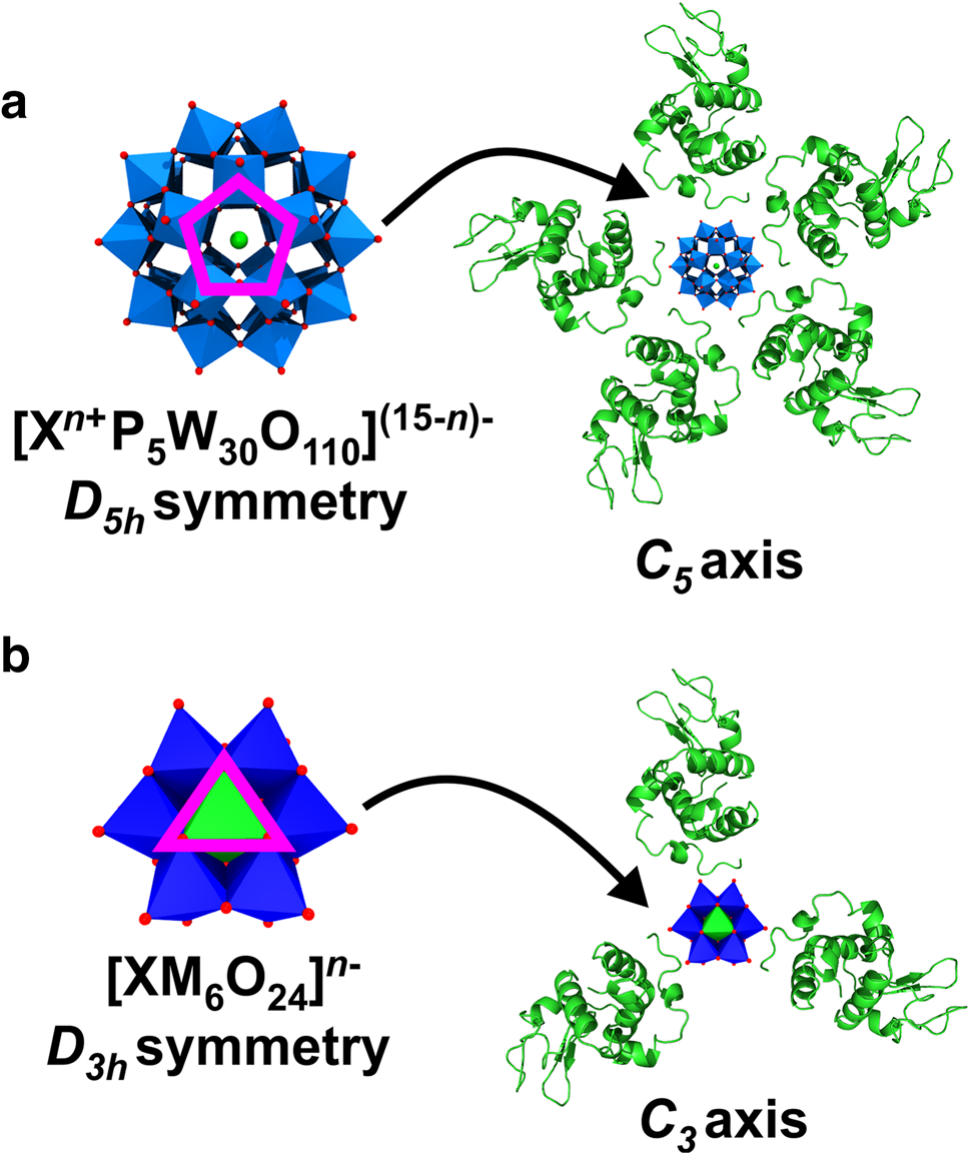
Schematic illustration of symmetry influencing the binding behavior of POMs.** a** Symmetry dictates the position of [X^*n*+^P_5_W_30_O_110_]^(15−*n*)−^ to a fivefold axis due to the anion’s internal *D*_5h_ symmetry. Being located on a fivefold axis the POM interacts with in total five (surrounding) protein molecules. The *C*_*5*_ symmetry of the Preyssler anion is indicated by a pink pentagon within its structure. The phosphorous atoms are not seen from this perspective as they are located behind tungsten octahedra. It has to be noted that crystals do not show fivefold symmetry axis and that this example here refers to an internal symmetry of an e.g. multi-domain protein (whereby the single domains are represented by hen egg white lysozyme molecules, green molecules). **b** Symmetry dictating the position of [XM_6_O_24_]^*n*−^ to a crystallographic threefold axis due to the anion’s internal *D*_3h_ symmetry. Being located on a threefold axis the POM interacts with in total three (surrounding) protein molecules. The *C*_3_ symmetry of the Anderson–Evans anion is indicated by a triangle within its structure. Hen egg white lysozyme is used as protein example and depicted as green cartoons. Color code: dark blue, M; light blue, tungsten; green, X; red, oxygen. Point groups and symmetry symbols are explained in [115]

Symmetry can also play an important role in POM-mediated protein crystallization as it is able to selectively dictate the POM’s binding site within the crystal lattice [106]. If the internal symmetry of the POM correlates with the crystallographic and/or non-crystallographic symmetry of the protein crystal (or with the internal symmetry of macromolecular assemblies), there is a certain probability that the POM will be found on the respective symmetry element. For example, the trigonal cluster [W_3_O_2_(O_2_CCH_3_)_6_]^2+^ exhibiting *D*_3_ symmetry and the pentagonal heteropolytungstate [NaP_5_W_30_O_110_]^14−^ (the so-called Preyssler anion) with *D*_5_ symmetry bind at the three- and fivefold rotation axis of riboflavin synthase, respectively [114]. Symmetry can also affect the ‘crosslinking’ degree of a POM since a polyanion located on an X-fold axis could interact with X symmetry related protein molecules (Fig. 15). Please note that a symmetry match between the POM and the crystal lattice (or the internal symmetry of the biomolecular assembly) is not a prerequisite for POM binding or its ability to facilitate protein crystallization as in most cases the POM’s binding site appears to be just random.

Two major issues of protein crystallization are the flexibility and dynamic behavior of proteins. Proteins having a high degree of flexibility in their structure are more resistant to crystallization as flexible regions are not easily stacked into a periodic lattice (crystal). The flexible nature of proteins is also reflected in the final protein crystal structure, which often suffers from incompleteness as very flexible domains, especially unstructured surface loops, are not modelled due to their increased mobility and vibrations. The time scale for these motions and vibrations is significantly shorter than the duration of the X-ray diffraction experiment, and therefore, the positions of these atoms are registered over a larger volume of space, which makes it difficult or even impossible to find a reliable position for very flexible fragments. Thus, these parts of the proteins are omitted during structure building and modelling leading to incomplete protein models. POMs are able to rigidly fix flexible protein regions by binding to them [41]. In this way, the conformational stability of the protein structure is increased and the crystallization probability enhanced. As structural flexibility is often associated with the protein’s functionality, POMs were also able to inhibit the function of certain enzymes by blocking essential domain motions, leading to crystal structures of certain transition states [41]. For example, binding of [H_2_W_12_O_40_]^6−^ did not only inhibit the activity of NTPDase1 but led also to the stabilization of a half-open conformation that was crystallographically solved [69]. The resulting crystal structure provided valuable insights into the enzyme’s domain motions during the catalytic activity [69]. To sum up, POMs influenced the crystallization of some proteins mainly due to their ability to mediate crystal contacts by ‘crosslinking’ protein molecules via noncovalent interactions and rigidifying flexible protein regions.

## The potential of hexatungstotellurate as a powerful crystallization additive

**Fig. 16:**
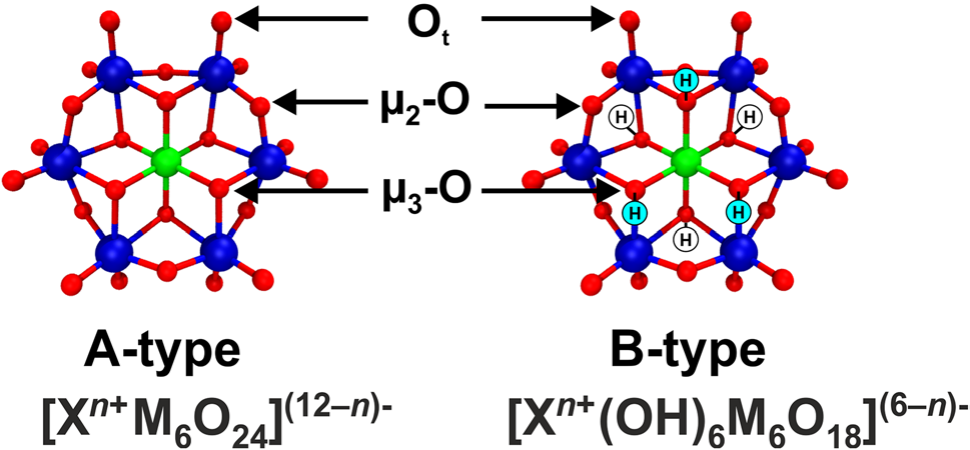
Ball and stick representation of the A- and B-type Anderson–Evans structure. The up to six protons in the B-type structures, which are attached to µ_3_-O atoms, are indicated by “H” (white circles indicate protons that point into the plain of drawing, whereas light blue circles indicate protons pointing out of the plain of drawing). The different coordination modes of the oxygen atoms are also assigned, where µ_3_-O are triple bridging oxygen atoms connecting the heteroatom atom with two addenda atoms, µ_2_-O are double bridging oxygen atoms connecting two addenda atoms and O_t_ are terminal oxygen atoms that are pairwise bound to each addenda atom. Color code: dark blue, M; green, X; red, oxygen

In the search for a suitable crystallization additive, several POM archetypes were tested with respect to their effect on the crystallization of some proteins [42, 70–73]. The results revealed that the Anderson–Evans type POM, hexatungstotellurate [TeW_6_O_24_]^6−^ (TEW), was the most suitable crystallization agent so far. It led to the crystallization of two hitherto structurally unknown proteins, namely mushroom tyrosinase from *Agaricus bisporus* (*Ab*PPO4) [71, 116] and aurone synthase from *Coreopsis grandiflora* (*Cg*AUS1) [73, 103]. In addition, TEW mediated the crystallization of the model protein hen egg white lysozyme (HEWL) into a previously unknown crystal form [72] and led to a different crystal packing during the crystallization of the nucleotide binding domain of the heat shock protein 70 [77]. The structure of TEW, an Anderson–Evans POM, was already described before (Fig. 1b). There are two types of the Anderson–Evans structure, the unprotonated A-type with the heteroatom *X* being in its highest oxidation state, [X^*n*+^M_6_O_24_]^(12−*n*)−^ (M = Mo^VI^ or W^VI^; X = Te^VI^, I^VII^), and the protonated B-type, which contains up to six protons on the µ_3_-O atoms and a heteroatom exhibiting a lower oxidation state, [X^*n*+^(OH)_6_M_6_O_18_]^(6−*n*)−^ (M = Mo^VI^ or W^VI^; X = Cr^III^, Fe^III^) (Fig. 16).

TEW is an A-type Anderson–Evans polyoxotungstate and fulfills the most important prerequisites of a crystallization additive, namely high solubility and stability under most crystallization conditions and the ability to interact with proteins while preserving their integrity [41]. The synthesis of TEW is straightforward and involves the acidification of an aqueous solution (pH ~ 5) containing Na_2_WO_4_ and Te(OH)_6_ at a molar ratio of 6:1 [116]. The solution is then heated at 110 °C until three-quarters of the volume remained, followed by the crystallization of the final product (slow evaporation at room temperature). TEW is most commonly characterized by X-ray diffraction and/or infrared (IR) spectroscopy. TEW crystallizes in the triclinic space group P1 exhibiting the unit cell parameters *a* ~ 10.3 Å, *b* ~ 10.6 Å, *c* ~ 11.1 Å, α ~ 91°, β ~ 115° and γ ~ 105° [117]. The IR spectrum of TEW exhibits a fingerprint region that is characteristic for the tungstate framework of the Anderson–Evans structure. Stretching vibrations of the terminal W=O units appear at ~ 952 and ~ 940 cm^− 1^. The spectrum shows also peaks at ~ 884 cm^− 1^ and in the region of ~ 470–750 cm^− 1^, which correspond to the asymmetric and symmetric deformation of the W–O–W and W–O–Te bridging fragments [118]. Other techniques that can be applied to characterize TEW are elemental analysis, ^183^W nuclear magnetic resonance (^183^W NMR) spectroscopy and mass spectrometry.

Besides the recently discovered application of TEW in protein crystallography, the Anderson–Evans POM is only extensively used as an inorganic building block for the synthesis of organic–inorganic hybrid POMs [118–123]. For this purpose, three to six protons of the B-type structure are replaced by one or two organic triol-ligands (RC(CH_2_OH)_3_ with R being any organic group).

There are several reasons that make TEW a powerful crystallization additive, which in some respects is superior to commonly used crystallization agents:

### TEW as phasing tool

TEW as other POMs and larger metal clusters can be utilized as a phasing tool, which has significant advantages over commonly used derivatization agents as discussed before. The six anomalously scattering tungsten atoms render TEW especially suitable for anomalous phasing. Due to the *L*–*I* absorption edge of tungsten at 1.02 Å, TEW can conveniently be used for SAD phasing even when the data is collected on a beamline that does not provide the ability to change the wavelength of the X-ray beam (data is collected only at *λ* ~ 1.0 Å). This represents a great advantage over most other anomalous scatterers as their absorption edges are located at distant wavelengths, and therefore, they (always) depend on wavelength-tunable X-ray sources.

### Eligible chemical properties of TEW for its use during protein crystallization

TEW is highly soluble in aqueous solutions, which is very important for crystallization reasons as the additive has to be used in large excess relative to the protein in most of the cases. The water solubility of the sodium salt of TEW, Na_6_[TeW_6_O_24_], is ~ 100 mM. Other well-known POM archetypes like the Wells–Dawson structure are in general significantly less water soluble exhibiting solubilities mainly in the range of 2–10 mM. Information about the solubility of most POMs can be obtained from [124]. In general, the solubility of POMs in aqueous solutions can be tuned by the choice of the counter-cation (e.g., H^+^, Na^+^, K^+^, etc.) [125].

TEW is stable at a pH range of 4.5–7.5 for at least several weeks at 4 to 20 °C (higher temperatures were not tested) [71, 72, 103, 116]. The POM structure is preserved in the crystallization solution as evidenced by crystal structures, determined from crystals that grew at different pH values. This represents a clear advantage over other POMs, especially the Keggin and the Wells–Dawson anion, which are only stable at acidic pH and undergo at least partial decomposition at neutral pH [32]. According to the PDB, most proteins have been crystallized in the range of pH 4–9, which is largely covered by TEW further demonstrating its suitability as a crystallization additive.

**Fig. 17:**
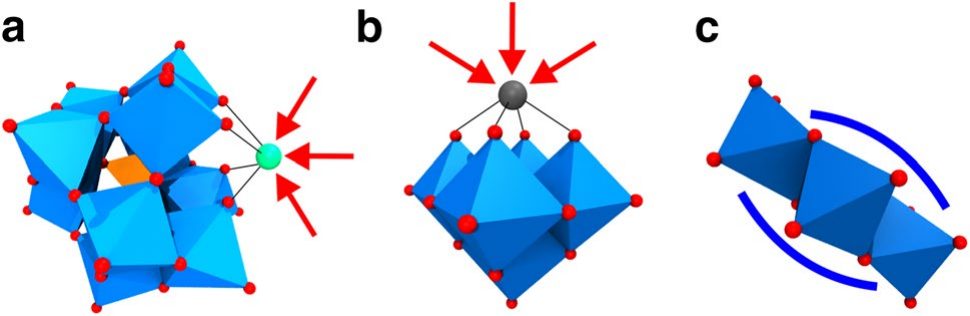
Structural comparison between hydrolytically active POMs and TEW. **a** Structures of the proteolytic Keggin [Ce(PW_11_O_39_)]^3−^ and **b** Lindqvist [Zr(W_5_O_18_)]^2−^ is depicted. In both cases the hydrolytically active metal ion (Ce^IV^ and Zr^IV^, shown as light cyan and grey sphere, respectively) is highly accessible (indicated by red arrows) allowing it to directly interact with the protein and eventually causing its cleavage. Please note that for both Ce^IV^ and Zr^IV^ the coordinated solvent molecules are omitted for clarity (coordination number = 7–8). **c** The TEW structure [TeW_6_O_24_]^6−^ are depicted for comparison, where a hydrolytically active metal can only be incorporated into the center of the structure. The central atom (not seen from this perspective) is shielded by the POM scaffold (indicated by blue bows) impeding its direct interaction with the protein. Color code: blue, tungsten; orange, phosphorus; light cyan, cerium; grey, zirconium; red, oxygen

TEW as most of the POMs is highly negatively charged and thus able to electrostatically interact with proteins as discussed above. Since the relatively high charge of TEW, and POMs in general, is distributed over a large size, TEW can interact with large protein patches. This enables TEW to interact with numerous amino acids simultaneously, which is an advantage over commonly used (protein bridging) additives like small molecules or ions, which depending on their relatively small charge and size provide a more tightly limited number of interactions sites. Crystallization additives must not interfere with the protein leading to its precipitation or denaturation during the crystallization. X-ray structure analysis and SDS–PAGE experiments of different TEW-protein complexes (hen egg white lysozyme, various polyphenol oxidases, aurone synthase, and human serum albumin) revealed that the integrity of the protein was preserved as no significant conformational changes were observed upon interaction with TEW [42, 71, 72, 103]. However, there are some POMs, especially those containing strong Lewis acids in the structure, which hydrolytically cleave proteins and are thus classified as artificial proteases. An example is provided by the dimeric Keggin-type POT [Ce(PW_11_O_39_)_2_]^10−^, which in solution decomposes into the monomeric and hydrolytically active form [Ce(PW_11_O_39_)]^3−^ [126]. This monomeric POM cleaves regioselectively hen egg white lysozyme due to the high Lewis acidity of the incorporated Ce^IV^ atom. Most of the hydrolytically active POMs are Keggin-, Wells–Dawson- or Lindqvist-type structures, where at least one addenda atom is substituted by a hydrolytically active metal, which in the POM’s monomeric form is highly accessible and thus active on proteins (Fig. 17a, b) [127]. In contrast, the addenda atoms of the Anderson–Evans POM cannot be substituted by metals other than molybdenum and tungsten. Thus, metals with high Lewis acidity can only be incorporated into the Anderson structure as the central heteroatom. Due to the planar disc-shaped structure of the Anderson–Evans POM, the heteroatom is surrounded and thus shielded by the six addenda atoms (Fig. 17c). Under these circumstances, the heteroatom of the Anderson–Evans structure is hardly able to directly interact with the protein, especially the protein’s backbone. This shielding effect was verified for different Anderson–Evans polyoxomolybdates containing partially strong Lewis acids as heteroatom (Fe^III^Mo_6_, Mn^III^Mo_6_, Ga^III^Mo_6_, Cr^III^Mo_6_) as none of the tested structures exhibited any hydrolytic activity towards proteins [119, 120, 128, 129]. Therefore, it seems that the Anderson–Evans archetype is in general one of  the safest POM with respect to the preservation of the protein’s integrity, which further strengthens the suitability of this POM-archetype as crystallization agent.

**Fig. 18:**
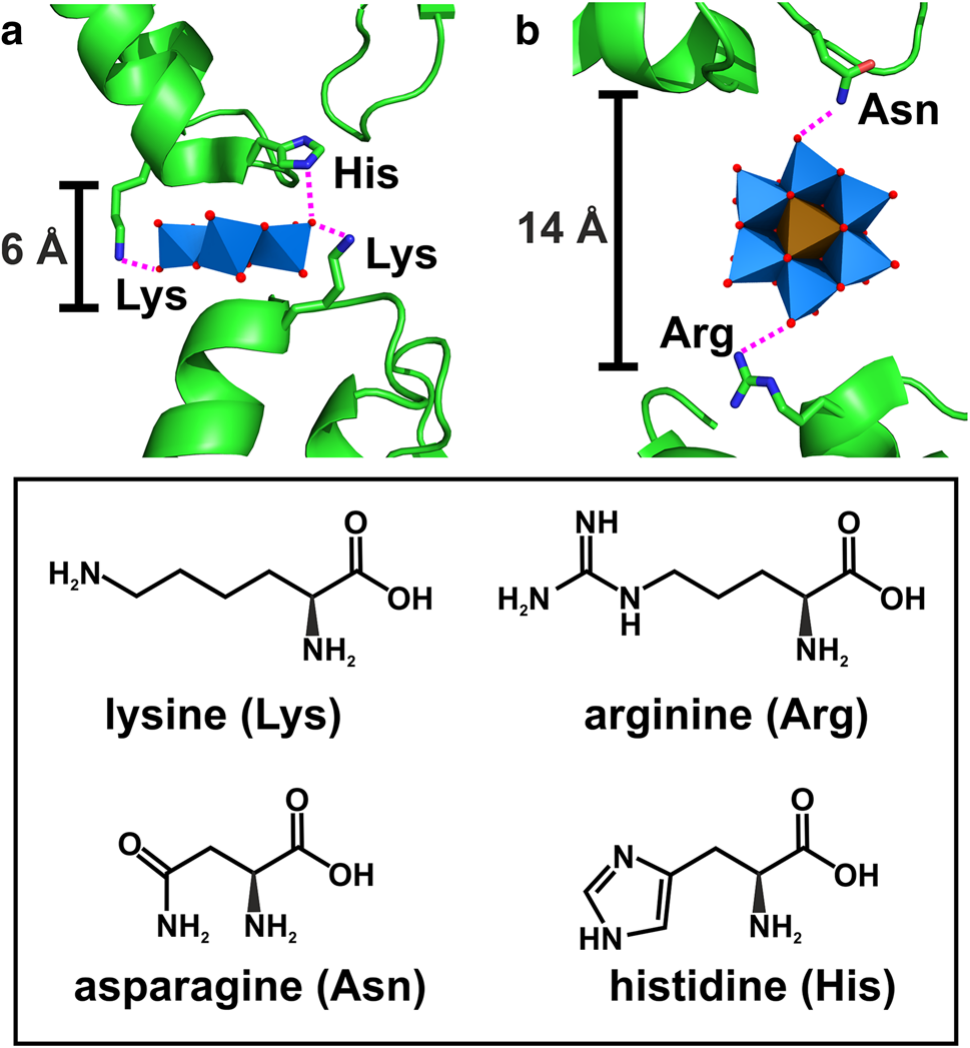
Protein–protein bridging by TEW at different orientations. **a** Two *Ab*PPO4 molecules (green cartoons, PDB entry 4OUA) are ‘crosslinked’ by one TEW molecule. TEW is horizontally positioned (flat side) between the protein molecules resulting in a small protein–protein distance of ~ 6 Å. **b** Two hen egg white lysozyme molecules (green cartoons, PDB entry 4PHI) are ‘crosslinked’ by one TEW molecule. TEW lies vertically between the molecules yielding a larger protein–protein distance of ~ 14 Å. Only a portion of the TEW–protein interactions are depicted for clarity reasons (dashed pink lines), whereby the interacting amino acids are illustrated in stick mode. In addition, the structural formulae of the involved amino acids are depicted. Color code: light blue, tungsten; brown, tellurium; blue, nitrogen; red, oxygen

### Beneficial size, shape and symmetry of TEW for protein crystallization

The special shape of the Anderson–Evans POM harbors even more advantages. As discussed before, POMs in general are able to provide a certain space between ‘crosslinked’ protein molecules (Fig. 14). The Anderson–Evans structure, due to its disc-like shape, offers a wide and a flat side and can thus provide a large and a small spacing between the protein molecules, respectively (Fig. 18). Depending on its orientation the spacing between the ‘crosslinked’ protein molecules can vary from ~ 6 to ~ 14 Å, which might have beneficial effects on the crystallization process due to the enhanced freedom in TEW-mediated protein–protein distances.

As discussed before, the symmetry can also play an important role in POM-mediated protein crystallization by influencing the POM’s binding site and interaction behavior. In Fig. 15 the hypothetical location of the Anderson–Evans anion on a crystallographic threefold-axis was shown as an example but this was not observed experimentally so far. However, during the crystallization of mushroom tyrosinase *Ab*PPO4 with TEW, two TEW molecules were located on the same crystallographic twofold axis with each TEW molecule interacting with two protein molecules [68, 71]. The internal approximate *D*_3d_ symmetry of TEW containing three *C*_2_ axes was compatible with that of the protein crystal, which was crystallized in the monoclinic space group *C*_2_. In the remaining reported TEW-containing protein crystal structures the TEW molecules are located on random positions (from a symmetry point of view) but interact in most cases with multiple numbers of protein molecules.

### The ability of TEW to induce heterogeneous protein crystallization

**Fig. 19:**
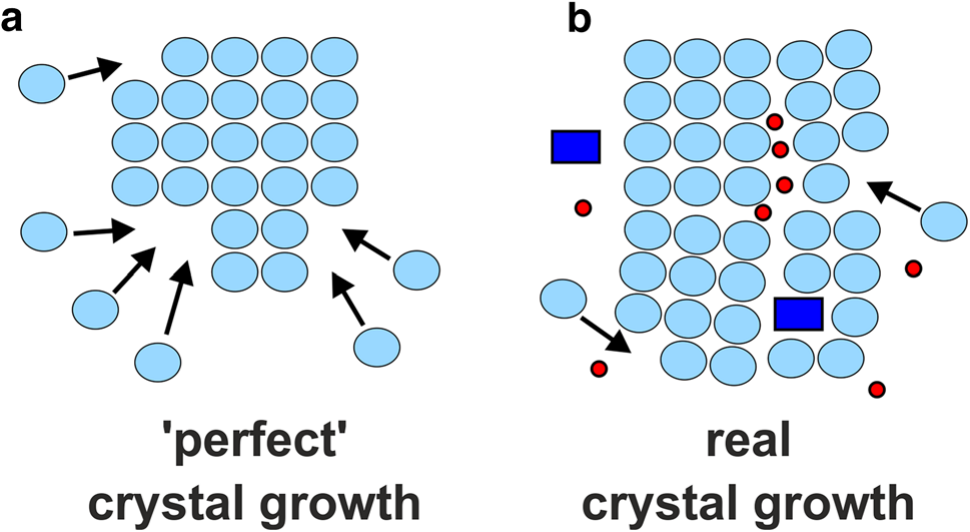
Crystal growth schemes. **a** A non-real ‘perfect’ crystal growth is depicted, which results from a solution that contains only one single specimen of protein molecules (light blue spheres) lacking any other disturbing entities. This scenario leads to a ‘perfect crystal’ as the protein molecules are perfectly aligned and arranged in the 3D space (2D in the figure). This figure is supposed to illustrate why crystallization seeks for homogeneity. **b** A real life mosaic crystal growth is illustrated, which results from a solution containing both small (red circles) and large (blue squares) impurities. It can be seen that even small impurities lead to growth defects and imperfections in the form of holes and misaligned ‘crystal blocks’ (= mosaicity, which describes the random misorientation of such blocks building up the mosaic crystal). As long as these imperfections are kept within limits, a high-quality crystal can still be formed. However, if such contaminant-mediated misalignment gets too large, the crystal will become highly mosaic making it unusable for the diffraction experiment. As the crystallization solution of *Ab*PPO4 was supposed to contain only the latent form, the presence of the active form represented some kind of large protein-based detritus. Therefore, it was more likely that the active form would strongly interfere with the crystallization of the latent form than forming a crystallizable heterodimer

TEW induced a crystallographic peculiarity during the crystallization of mushroom tyrosinase *Ab*PPO4. Tyrosinases are enzymes that convert mono- and diphenols to the respective quinones, which then undergo further non enzymatic reactions to form melanin. Fungal tyrosinases like *Ab*PPO4 exist in both a premature inactive (latent) form and an active form [71]. In the latent form the enzyme’s C-terminal domain covers its catalytic active site, which harbors the dicopper center responsible for its activity, and thus prevents substrates (mono- and diphenols) from entering it leading to the enzyme’s latency. Therefore, it is believed that a yet unknown protease cleaves off the C-terminal domain to activate the enzyme by making the active site accessible to its substrates. To study not only the catalytic but also the maturation (activation) process of this enzyme, there was a high interest in both the crystal structure of the latent (64 kDa) and active form (44 kDa) of this type of enzymes. Back then, no crystal structure of *Ab*PPO4 was obtained until TEW was used as crystallization additive, which unexpectedly induced the crystallization of both the latent and active form as a heterodimer within one single crystal [71, 116]. Thus, TEW has ‘killed two birds with one stone’. This is particularly noteworthy, not only because the latent and active form do not form biological dimers (with each other), but also due to the fact that crystallization trials usually strive for homogeneity to efficiently build up a highly ordered single crystal. This works best with one single building block (one single protein or biological oligomer) as other larger and/or protein contaminants (e.g., the active form) could interfere with the lattice formation, thereby disrupting and impeding the crystallization process (Fig. 19).

**Fig. 20:**
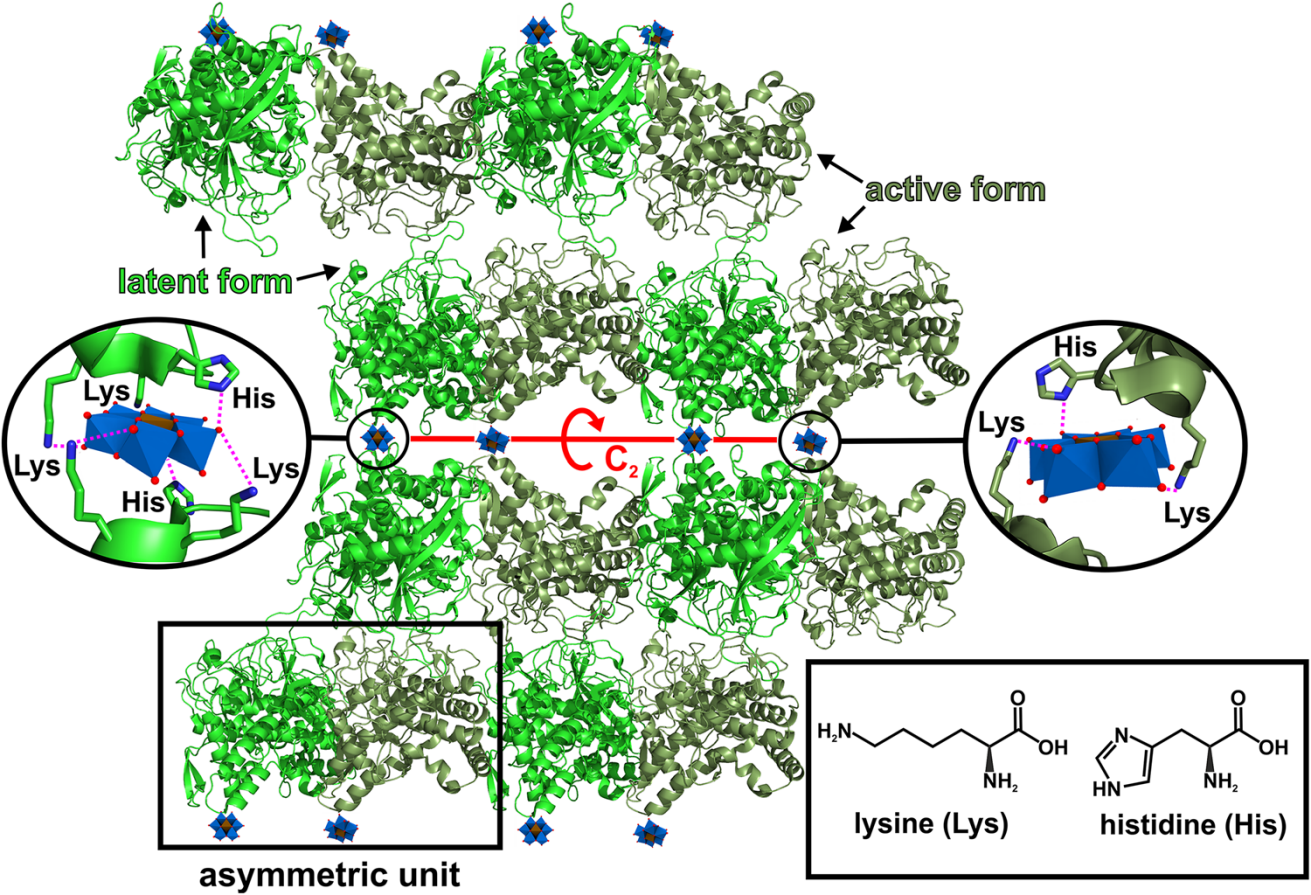
TEW-mediated heterogeneous crystallization of the latent and active form of *Ab*PPO4. An excerpt from the crystal packing of the *Ab*PPO4–TEW complex is depicted with both the latent (light green cartoon) and the active form (dark green cartoon) being present in the crystal structure. Each TEW molecule interacts with two latent or active *Ab*PPO4 molecules via electrostatic interactions with lysine (Lys) and histidine (His) residues as shown in the round insets. In total, one heterodimer is connected to another dimer by two TEW molecules from one side, whereas from the other side the heterodimer interacts with the next dimer via common protein–protein interactions. The TEW molecules lie on a crystallographic twofold-axis which is delineated in red in the middle of the figure. TEW molecules are shown as polyhedra, whereas the interacting lysine and histidine residues are illustrated in stick mode. In addition, the structural formulae of the involved amino acids are depicted. Color code: blue, tungsten; brown, tellurium; dark blue, nitrogen; green, carbon; red, oxygen

This special arrangement was only possible by the use of TEW as each heterodimer is on the one side connected to a symmetry related heterodimer by a TEW-mediated contact composed of two TEW molecules (one TEW binds the latent while the other one binds the active form in the heterodimer). On the opposed side the heterodimer is connected to another dimer by a common protein–protein contact (Fig. 20). Two molecules of the latent or active *Ab*PPO4 share one TEW molecule, which is located on a crystallographic twofold axis as mentioned before. This pattern represents the building block for the entire crystal. This special case demonstrates the ability of TEW to induce heterogeneous crystallization (crystallization of at least two proteins within one crystal), which might be of great importance with respect to the crystallization of multi-domain structures or large heterogeneous molecular assemblies.

### The ability of the geometric and functional flexibility of TEW to influence protein crystallization

**Fig. 21:**
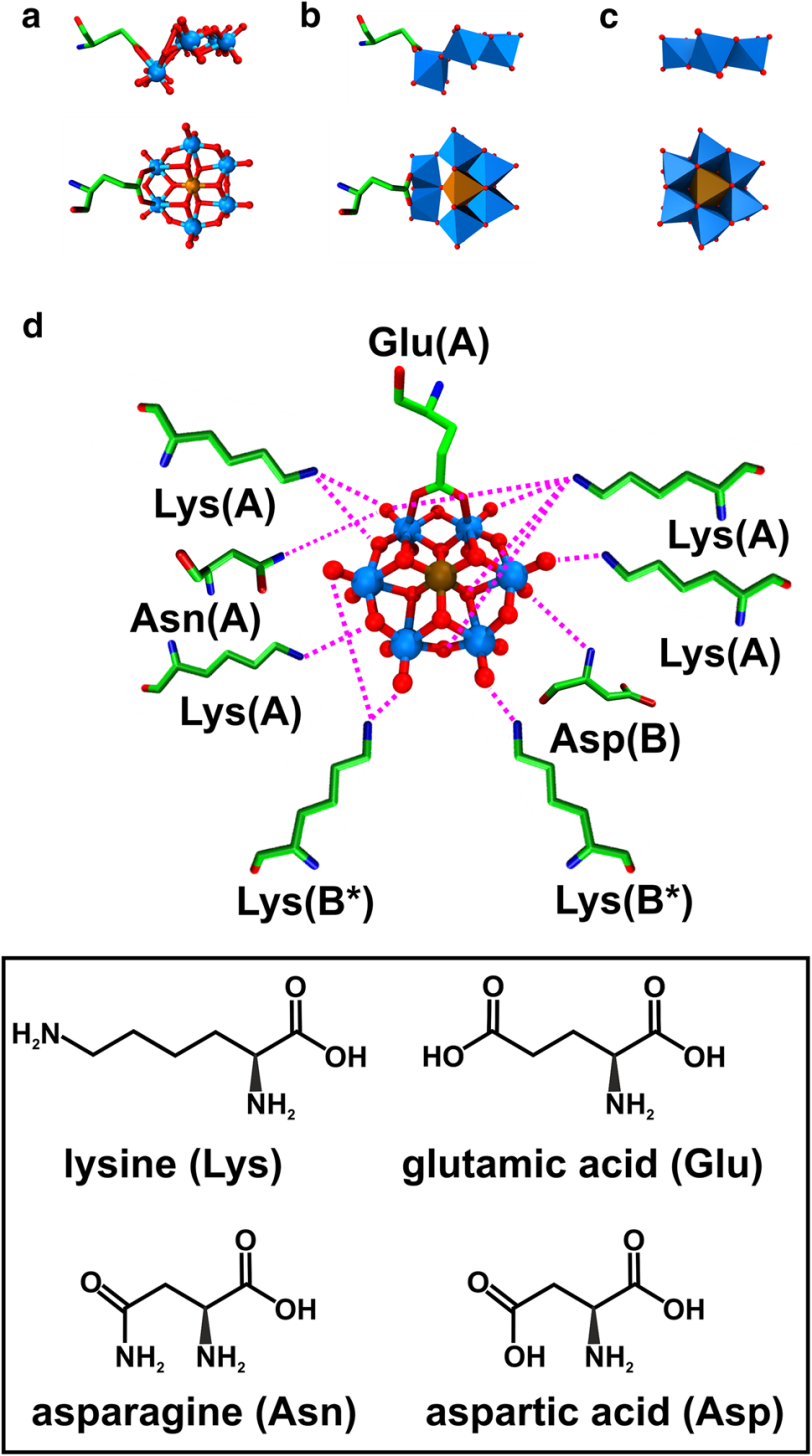
Covalent binding of TEW to *Cg*AUS1. **a** The covalent bond between the carboxylic oxygen atoms of glutamic acid (Glu) and two tungsten atoms of TEW is depicted. The binding leads to an unprecedented amino acid bound and bent Anderson–Evans structure (top: side view, bottom: view from above). **b** Same as figure **a** with TEW being illustrated in polyhedra mode. **c** The normal Anderson–Evans structure is shown for comparison. **d** Overview of all direct TEW–protein interactions within the binding cleft (solvent mediated interactions are omitted for clarity). Note that aspartic acid (Asp) is hydrogen bonding to TEW via its peptide amide group (primary/backbone amide), whereas the remaining amino acids interact via their side chain groups (electrostatic interactions and hydrogen bonds). The parentheses indicate which molecule provides the respective amino acid as the protein was crystallized as a dimer (two protein chains, A and B), whereas an asterisk indicates that the amino acid is coming from a protein chain that is located in an adjacent asymmetric unit. The structural formulae of the involved amino acids are also depicted. Color code: blue, tungsten; brown, tellurium; green, carbon; dark blue, nitrogen, red, oxygen

During the crystallization of *Cg*AUS1 with TEW, one TEW molecule was unexpectedly found to be covalently bound to the protein [73]. Two tungsten atoms of TEW are bound by the two carboxylic oxygen atoms of a glutamic acid leading to the formation of the [TeW_6_O_24_O_2_(Glu)]^7−^ moiety (O_2_ = carboxylic oxygens of Glu = glutamic acid). This was the first case of a covalently protein-bound POM, where the POM was applied as a preformed cluster and was not *in situ* formed during the crystallization process. Interestingly, the covalent binding of TEW to the protein is accompanied by a structural rearrangement of the Anderson–Evans structure leading to the transition of the planar POM into an unprecedented bent structure (Fig. 21a–c). The mechanism for this covalent bond formation is not known but it was suggested that the bond was sterically forced by the environment of TEW. The polyanion is located within a narrow and highly positively charged cleft, where it strongly interacts with the surrounding amino acids (Fig. 21d). The crystallographic results indicate that TEW was able to structurally adopt to the protein to fit into the binding cleft as the overall structure of the protein was not changed. Thus, the disc-shaped structure of the Anderson–Evans POM is able to undergo surprising conformational changes under certain circumstances, however, the driving force of this structural change is still not fully understood. The ability of TEW to form covalent bonds with amino acid side chains could also contribute to the crystallization promoting effects of this molecule, for example, by covalent fixation of flexible protein domains that otherwise would hamper the crystallization process. Furthermore, TEW might be able to provide occasional covalent crosslinks between protein molecules, leading to significantly more stable crystal contacts.

### The ability of TEW to improve the crystal quality

**Fig. 22:**
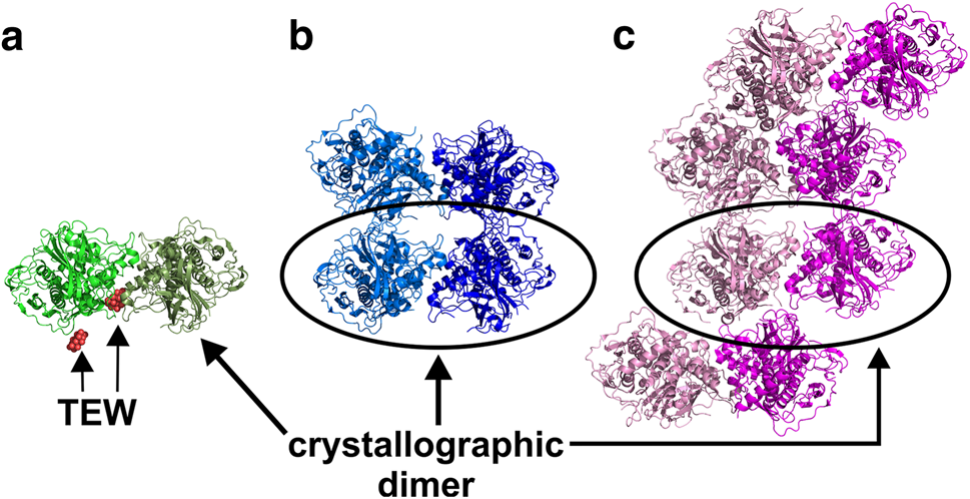
Comparison of the asymmetric unit contents of the TEW-mediated and TEW-less crystal forms of *Cg*AUS1. **a** Asymmetric unit of the TEW-mediated *Cg*AUS1 crystal. The positions of the POMs are indicated by clusters of red spheres, whereby one TEW molecule is stabilizing the crystallographic dimer. **b** Asymmetric unit of the TEW-less crystal containing four protein molecules (two crystallographic dimers). **c** Asymmetric unit of the TEW-less crystal containing eight protein molecules (four crystallographic dimers). Every crystal form shares the same crystallographic dimer, whereby different protein chains of the dimers are shown in different color shades (green/dark green, blue/dark blue, pink/dark pink)

TEW is able to improve the crystal quality in comparison to POM-free crystals. Aurone synthase *Cg*AUS1 was crystallized in three different crystal forms, two in absence and one in presence of TEW [130]. The crystallization conditions of all three crystal forms were essentially the same with the exception that the TEW-less approaches contained the additive MgCl_2_ instead of TEW. The resulting TEW containing crystals were of significantly higher quality leading to an improvement in resolution by up to ~ 1.0 Å in comparison to the TEW-less crystals [73]. Crystal structure analysis and comparison between the three different crystal forms revealed that the crystal contacts in the TEW containing crystals are more specific than those of the crystals lacking the POM [131]. All crystal forms are built up by the same crystallographic dimer, however, the asymmetric unit (ASU) of each crystal form differed in their respective dimer content. The ASU of the *Cg*AUS1-TEW crystal contained only one crystallographic dimer (two protein molecules), whereas the ASUs of the TEW-less crystals consisted of two and four dimers (four and eight protein molecules), respectively. This observation indicates that the *Cg*AUS1-TEW crystal has the highest symmetry due to the lowest ASU content, which means that in this case a smaller ensemble (smaller ASU with less molecules) is required to build up the whole crystal in comparison to the TEW-less crystals (Fig. 22). Two TEW molecules are located in the *Cg*AUS1-TEW crystal structure and provide new crystal contacts, whereby one anion strongly stabilizes the crystallographic dimer. The TEW-mediated crystal contacts, especially the TEW-stabilized dimer contact, seem to be the reason for the increased crystal quality as they represent by far the strongest crystal contacts (judged by the contact area and number of participating amino acids) within the crystal. Thus, the TEW-mediated crystal contacts dictate the crystal formation as they provide a dominating adhesion mode between the single protein molecules [132]. This means that the protein molecules bind to each other and assemble predominantly via TEW-mediated contacts. In contrast, the crystal contacts of the TEW-less crystals lack a preferred adhesion mode leading to several more unspecific protein–protein contacts. As a result, the protein molecules bind to each other more randomly as more (equivalent) interaction sites are provided (on the growing crystal surface) ultimately leading to a number of stacking faults and lower long-range periodicity, which is necessary for good diffracting crystals. The same could also be true for mushroom tyrosinase *Ab*PPO4 as very recently a crystal structure of its latent form was obtained without the use of TEW but at a significantly lower resolution (2.8 vs. 3.3 Å) [133]. Unfortunately, the TEW-containing and TEW-less structure could not be compared due to the different crystal content as the latter is composed of only the latent form [133]. Nevertheless, it seems that TEW-mediated contacts are crucial for the crystal quality in the above discussed cases.

### The ability of TEW to contribute a significant entropy gain during protein crystallization

TEW exhibits a significant impact on the solvent-driven crystallization entropy, which represents the driving force for the crystallization. Thus, TEW is able to energetically favor the crystallization process of some proteins [131]. As already mentioned growing crystals is restrained by the fact that proteins, due to their irregular distribution of hydrophilic and hydrophobic patches on their surface, offer only a small number of crystal contacts, which are held together by weak noncovalent interactions [6]. The free energy of crystallization $\Delta G_{{{\text{cryst}}}}^{o}$ depends on both enthalpic ($\Delta H_{{{\text{cryst}}}}^{o}$) and entropic ($- T\Delta S_{{{\text{cryst}}}}^{o}$) contributions, and can be formulated as [134]:

$$\Delta G_{{{\text{cryst}}}}^{o}=\Delta H_{{{\text{cryst}}}}^{o} - T{\left( {S_{{{\text{protein}}}}^{o}+S_{{{\text{solvent}}}}^{o}} \right)_{{\text{cryst}}}}$$

**Fig. 23:**
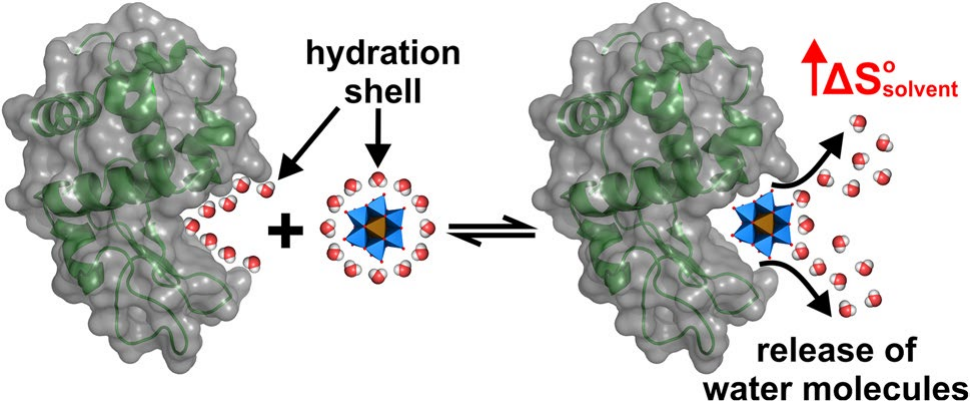
Schematic illustration of TEW’s contribution to the solvent-driven entropy of the crystallization system via water release. Before binding both the TEW and the protein molecule are surrounded by a hydration shell (illustrated as small water molecules). Upon TEW-binding, the release of water molecules from their hydration shells (partial dehydration) causes a gain in solvent entropy. The binding of a second protein molecule, which induces a further solvent release and completes the crystal contact, is omitted for clarity. The protein (hen egg white lysozyme as an example) is shown as green cartoon in combination with a surface representation (transparent grey surface), whereas TEW is illustrated as polyhedra. Color code: blue, tungsten; brown, tellurium; red, oxygen; white, hydrogen

To make crystallization happen, $\Delta G_{{{\text{cryst}}}}^{o}$ has to be negative, however, the term $\Delta H_{{{\text{cryst}}}}^{o}$ will be, at best, only moderately negative due to the low number and weak strength of the formed intermolecular crystal contact interactions. Aggravating this situation, the transition of protein molecules from an aqueous solution into a rigid and well-ordered crystal lattice reduces the entropy due to the loss of the protein molecule’s degree of freedom, leading to an unfavorably negative $S_{{{\text{protein}}}}^{o}$ term. Therefore, the entropy loss has to be compensated by the solvent entropy $S_{{{\text{solvent}}}}^{o}$, which increases with the number of released solvent molecules from the hydration shell of the proteins upon the formation of protein–protein crystal contacts. Only if the formed crystal contacts induce a sufficiently high $S_{{{\text{solvent}}}}^{o}$, which makes the total free energy of the crystallization term negative, crystal formation will occur. In the presence of TEW and upon the formation of TEW-mediated protein–protein contacts, this gain in solvent-driven entropy $S_{{{\text{solvent}}}}^{o}~$is significantly increased in comparison to the situation without TEW, which was shown on three proteins (*Cg*AUS1, *Ab*PPO4 and HEWL) [131]. The increased solvent release during TEW-mediated crystal packing is significantly higher than that induced by most of the commonly used additives (e.g., different small anions and cations like I^−^, ${\text{NO}}_{3}^{ - }$, Zn^2+^, Y^3+^, etc.). This effect is, among others, related to the large size of TEW and the associated large TEW-protein contact area as more solvent molecules can be released from both the protein’s and TEW’s hydration shell (Fig. 23). Other POMs could have the same effect as the entropy gain largely depends on the size and binding affinity to the protein, however, this was not investigated for other protein-POM systems.

Due to the above summarized features, TEW is in many respects predestined as a powerful crystallization additive bringing advantages over other POM archetypes and commonly used crystallization additives. Therefore, crystallographers will benefit from its future use. Based on its successful use in protein crystallography, TEW was introduced into the market and is now commercially available as part of a crystallization screen offered by Jena Bioscience (http://www.jenabioscience.com).

## Notes for the practical use of polyoxometalates in protein crystallization

As shown in this article, POMs represent a promising group of potential crystallization additives. Most of their properties and features can be tuned to the crystallographer’s favor, for example, their solubility by altering their counter-cations, their charge by combining appropriate addenda and heteroatoms and their shape and chemical properties by modifying their surface (e.g., substitution of an addenda atom by another metal, etc). Despite the promising features of POMs, care should be taken when applying them to a crystallization trial as most POMs display an unpredictable behavior in aqueous solutions. The chemistry of POMs in aqueous solution is very complex and depends highly on the used addenda atoms and pH. In general, POMs exhibit several polyoxo species in certain pH regions, which coexist in solution [135]. The pH dependent instability was also observed for some other POM archetypes at neutral or basic pH (e.g., Keggin and Wells–Dawson structure decompose into low-nuclear species) making it difficult to define the exact POM species in solution. This represents a particular issue regarding their use in protein crystallization as most of the proteins are crystallized at approximately neutral pH. Unfortunately, there is no reliable technique to quickly check the predominant POM species in solution without prior knowledge about the composition–decomposition equilibria of the given POM. Classical chemical approaches like elemental analysis can provide valuable information about the POM’s composition. The most reliable and commonly used method to determine the POM structure is single crystal X-ray crystallography, however, the crystal structure only provides the POM’s structure in the solid state and does not guarantee that the observed structure is the predominant one in solution. Furthermore, the crystallized POM might represent only the species most prone to crystallization within a solution containing more coexisting POM species. Other techniques, which are commonly used to determine the POM structure, are NMR, UV/Vis, IR spectroscopy, mass spectrometry and small angle X-ray scattering (SAXS). All of these methods provide valuable structural information about the POM, however, each technique alone is in the most cases not sufficient for structure verification. The best way is to use a combination of the above mentioned methods to reliably identify the correct POM structure.

Another aspect that should be taken into consideration is the size and shape of the applied POM. POMs, which are too large, will rather sterically disrupt favorable protein–protein contacts instead of mediating them, especially in the crystallization of small proteins, as they will not fit appropriately between the protein molecules within the crystal lattice. The largest POM that was applied in X-ray crystallography so far is the Preyssler anion [NaP_5_W_30_O_110_]^14−^ exhibiting approximate dimensions of ~ 15 × 18 × 10 Å, whereby it should be noted that it was solely used for the phasing of the very large riboflavin synthase (~ 1 MDa) [106, 114]. Moreover, the size of the POM is connected to its charge density, which in turn plays a decisive role in the POM’s interaction with proteins. Therefore, if a given charge is diluted over a too large surface, not only the POM’s charge density is reduced but maybe also its binding affinity towards proteins. As discussed before, there are hydrolytically active POMs that cleave proteins. Unfortunately, not all of them are obviously hydrolytically active like those containing an accessible strong Lewis acid, which should be avoided for protein crystallization. There are also inconspicuous and common POMs like the mentioned Preyssler anion that have been observed to induce partial denaturation of proteins (i.e., human serum albumin) [94]. Therefore, the POMs should also be tested in this regard prior to protein crystallization, for example, by SDS–PAGE or circular dichroism analysis to provide information on the stability of the protein in the presence of the POM. However, it has to be noted that some crystallization approaches include proteases to enable the crystallization of large (multi-domain) or highly flexible proteins. The strategy behind this is to excise highly flexible protein loops (in the case of flexible proteins) and/or to cut a large protein into smaller fragments (in the case of multi-domain proteins), which in both cases should make the protein more amenable to crystallization.

The crystallization buffer can also become a critical point as some POM-buffer combinations could lead to undesired results. The buffer components should not chemically interfere with the POM as they could distort the POM’s structure and functionality. Some polyoxovanadate species have been observed to form complexes with buffers like TRIS [135]. In addition, the buffer can also interfere with the POM’s function without directly interacting with it. For example, buffers containing volatile constituents change the pH of the solution over time, if not being renewed, which can cause POM decomposition as their stability is highly pH-sensitive. High concentrations of salt-based buffers, which are required for the crystallization of some proteins, are associated with a high ionic strength that at a certain extent can decrease the binding affinity of the POMs to the protein. This is due to the competition between the POMs and the anions of the salt for the (charged) binding sites of the proteins.

Thus, the use of POMs in protein crystallography requires planning and some experimental effort in advance to exclude negative effects of the POM on the protein and the crystallization process and to save time and resources (crystallography grade protein, crystallization solutions, consumables and above all, time and effort of the involved researchers).

## Concluding remarks

Due to their high versatility in both structure and physicochemical properties, which can be deliberately fine-tuned, POMs represent not only strong phasing tools but also a promising group of crystallization agents in protein crystallography. The targeted application of some POMs led to promising and desired results as the POMs were able to facilitate or, in some cases, were even necessary to enable the crystallization of some proteins (by promoting and strengthening crystal contacts and/or stabilizing the protein’s conformation). In this regard, the Anderson–Evans polyoxotungstate was shown to be especially suitable as it exhibited several advantages over other POM archetypes and commonly used additives. More experimental efforts are needed to make the usage of POMs more efficient. For example, a better understanding of the POM–protein or POM-mediated protein–protein interactions is required as there is no unambiguous correlation between the POM’s binding affinity to the protein and its structural and/or chemical features. It is true that most POMs were found at protein regions exhibiting a positive electrostatic potential. However, POMs were also found at sites with a more neutral electrostatic potential (polar or hydrophobic regions), despite the presence of more positively charged regions on the same protein. Therefore, also other factors seem to play an important role in POM–protein interactions, for example:

- The size compatibility between the POM and its binding site on the protein.
- The entropy effects due to the partial dehydration of the POM’s and protein’s hydration shells favoring their interaction.
- The charge density and chaotropic nature of some POMs that enable even hydrophobic interactions with the protein.

Furthermore, the behavior of POMs in aqueous solutions needs to be explored in greater detail. POMs show a pronounced tendency to undergo multiple condensation–hydrolysis (formation–degradation) equilibria leading to the co-existence of several POM species, which might interfere with protein crystallization due to the increased inhomogeneity of the crystallization solution upon addition of a labile POM. Nevertheless, the recent successful applications of POMs in protein crystallization could be the start of a bright future in the course of which several fields of chemistry, especially those depending on the input of 3D protein crystal structures (e.g., structural biology, biochemistry, medicine and pharmacy), could benefit from the use of POMs.

## Electronic supplementary material

Below is the link to the electronic supplementary material.

Supplementary material 1 (PDF 1530 KB)

## Abbreviations

Å: Ångström
*Ab*PPO4: Polyphenol oxidase (tyrosinase) from *Agaricus bisporus*
ASU: Asymmetric unit
*Cg*AUS1: Aurone synthase from *Coreopsis grandiflora*
HEWL: Hen egg white lysozyme
IR: Infrared
kDa: Kilodalton
MDa: Megadalton
MR: Molecular replacement
NMR: Nuclear magnetic resonance
POM: Polyoxometalate
POMo: Polyoxomolybdate
POT: Polyoxotungstate
PTM: Post-translational modification
SAD/MAD: Single-/ multiple-wavelength anomalous dispersion
SAXS: Small angle X-ray scattering
SIR/MIR: Single-/multiple isomorphous replacement
TEW: Hexatungstotellurate
