# Supplementary material for: Polyoxometalates: more than a phasing tool in protein crystallography
Source: ChemTexts. 2018 Aug 28;4(3):10. doi: 10.1007/s40828-018-0064-1 (PMC6294228; doi:10.1007/s40828-018-0064-1)
Supplement: Supplementary file 1 — Supplementary material 1 (PDF 1530 KB) [file 40828_2018_64_MOESM1_ESM.pdf]

Die Rolle von Polyoxometallaten in der Proteinkristallographie – Mehr als nur ein Werkzeug für die Lösung des Phasenproblems

Aleksandar Bijelic[a] und Annette Rompel[a]*

[a] Universität Wien, Fakultät für Chemie, Institut für Biophysikalische Chemie, Althanstraße 14, 1090 Wien Österreich.

* E-mail: annette.rompel@univie.ac.at

Homepage: http://www.bpc.univie.ac.at

Telefon: +43-1-4277-52502

ORCID: Aleksandar Bijelic: 0000-0002-8781-153X

Annette Rompel: 0000-0002-5919-0553

Dieser Artikel ist Michael Thor Pope anlässlich seines 85. Geburtstages gewidmet.

Zusammenfassung

Die Proteinkristallographie ist die am häufigsten verwendete Methode zur Aufklärung der molekularen Struktur von Proteinen und dem Informationserhalt über Protein-Ligand-Komplexe auf atomarer Ebene. Da die Struktur eines Proteins dessen Funktionen und Eigenschaften bestimmt, ist die Kristallographie von immenser Bedeutung für fast alle Forschungsgebiete im Bereich der Biochemie. Die Proteinkristallographie bringt jedoch einige Schwierigkeiten mit sich, wobei die Unberechenbarkeit des Kristallisationsprozesses selber den zentralen Engpass dieser Methode darstellt. Der Kristallisationsprozess ist weitestgehend immer noch ein „Trial-and-Error"-Verfahren und deshalb sehr zeit- und ressourcenaufwendig. Viele Strategien zur Verbesserung oder Ermöglichung der Kristallisation von Proteinen wurden in den letzten Jahrzehnten entwickelt. Dabei ist die Verwendung sogenannter Additive, die meistens kleine Moleküle sind und dafür sorgen, dass Proteine „kristallisierbarer" werden, die komfortabelste und erfolgreichste Methode. Die Verwendung der meisten gebräuchlichen Additive ist jedoch auf bestimmte Kristallisationsbedingungen oder Proteingruppen beschränkt. Deshalb würde ein universelleres Additiv, welches nicht nur eine deutlich größere Anzahl an Proteinen abdeckt, sondern auch über ein breiteres Spektrum an Kristallisationsbedingungen anwendbar ist, einen Durchbruch im Bereich der Proteinkristallographie darstellen. In den vergangenen Jahren haben sich Polyoxometallate (POMs) aufgrund ihrer einzigartigen Strukturen und Eigenschaften als eine Gruppe vielversprechender Kristallisationsadditive hervorgetan. In dieser Hinsicht bewies vor allem das Tellurium-zentrierte Anderson-Evans Polyoxowolframat $[TeW_6O_{24}]^{6-}$ (TEW) sein großes Potenzial als Kristallisationsadditiv. In diesem Lehrtext wird das Potenzial der POMs als Werkzeuge in der Proteinkristallographie herausgearbeitet, wobei ein besonderer Schwerpunkt auf dem bisher erfolgreichsten Cluster TEW liegen soll.

Keywords

Polyoxometallate - Hexawolframatotellurat - Anderson-Evans-Struktur - Proteinkristallisation - Kristallisationsadditive

Abkürzungsverzeichnis

Å	Ångström
*Ab*PPO4	Polyphenoloxidase (Tyrosinase) aus *Agaricus bisporus*
ASU	asymmetrische Einheit
*Cg*AUS1	Auronsynthase aus *Coreopsis grandiflora*
HEWL	Hühnereiweiß-Lysozym
IR	infrarot
kDa	Kilodalton
Krist	Kristall
MDa	Megadalton
MR	Methode des Molekularen Ersatzes (molecular replacement)
NMR	Kernspinresonanz (nuclear magnetic resonance)
POM	Polyoxometallat
POMo	Polyoxomolybdat
POW	Polyoxowolframat
PTM	Posttranslationale Modifikation
SAD/MAD	Methode der anomalen Dispersion bei einer oder mehreren Wellenlängen (single-/ multiple-wavelength anomalous dispersion)
SAXS	Röntgenkleinwinkelstreuung (small angle X-ray scattering)
SIR/MIR	Methode des Isomorphen Ersatzes mit einem oder mehreren Derivaten (single-/multiple isomorphous replacement)
TEW	Hexawolframatotellurat

Die Methode der Röntgenstrukturanalyse

Die Strukturbiologie beschäftigt sich mit der molekularen Struktur und Dynamik biologischer Makromoleküle, besonders derer von Proteinen und Nukleinsäuren. Die molekulare Struktur von Proteinen bestimmt deren Eigenschaften und Funktionen. Dies ist von großem Interesse für Wissenschaftler, die in den Biowissenschaften arbeiten, da Proteine an den grundlegendsten Prozessen des Lebens beteiligt sind. Des Weiteren sind Proteine die Ziele der meisten therapeutisch aktiven Verbindungen und deshalb ist strukturelles Wissen für die Untersuchung wichtiger Wechselwirkungen zwischen Medikamenten und bestimmten Proteinen unverzichtbar. Das gewonnene strukturelle Wissen wird dann verwendet, um entweder die Wirkung bereits bekannter Medikamente zu verbessern oder um neue Arzneistoffe zu entwickeln. Die Röntgenstrukturanalyse ist derzeit die am häufigsten verwendete Methode zur Bestimmung makromolekularer Strukturen, da sie relativ zuverlässig akkurate molekulare Strukturen von großen Proteinen oder sogar molekularen Komplexen (> 100 kDa) bei atomarer Auflösung liefert. Dies spiegelt sich auch in der Proteindatenbank (PDB, www.rcsb.org) wider, wo ~ 90% aller hinterlegten Proteinstrukturen mittels Röntgenkristallographie gelöst wurden. Die erste mittels Röntgenstrukturanalyse bestimmte 3D-Struktur eines Proteins war die des Myoglobins, die 1958 von John C. Kendrew (1917 - 1997) gelöst wurde [1]. Nur zwei Jahre später bestimmte Max F. Perutz (1914 - 2002) die Struktur des Hämoglobins [2]. Perutz und Kendrew erhielten 1962 den Nobelpreis für Chemie für ihre bahnbrechenden Arbeiten im Bereich der Röntgenstrukturanalyse. Seitdem wurden mittels der Röntgenstrukturanalyse Tausende von Proteinstrukturen bestimmt, sodass die PDB seit 1976 über 125,000 Einträge (Proteinstrukturen) zählt (Stand: Mai 2018). Die Röntgenstrukturanalyse eines Biomakromoleküls besteht im Grunde genommen aus fünf wesentlichen Schritten, (1) dem Erhalt ausreichender Mengen des Zielproteins, (2) der Proteinreinigung, (3) der Kristallisation, (4) der Datensammlung und (5) der Strukturbestimmung (Abb. 1).

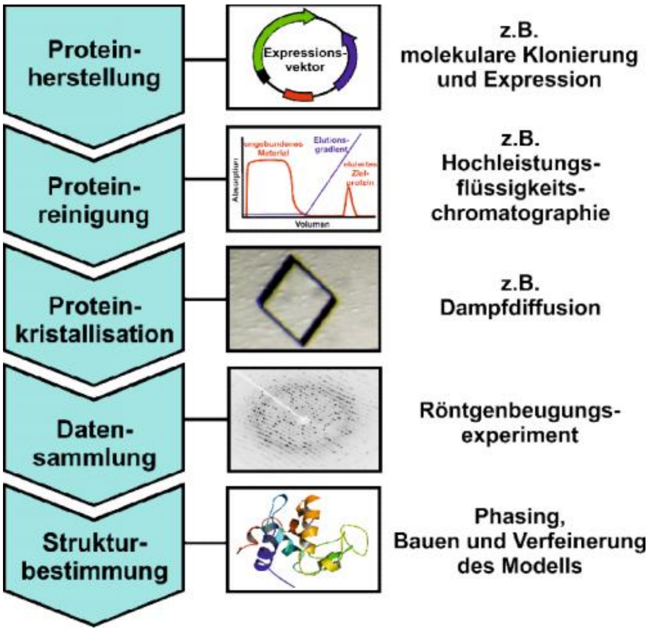

Abb. 1 Ablaufplan der Röntgenstrukturanalyse eines Proteins. Die Herstellung des Proteins ist durch einen einfachen Expressionsvektor (molekularbiologischer Ansatz) dargestellt. Der Schritt der Proteinreinigung ist durch das Chromatogramm einer Affinitätschromatographie symbolisiert, wo das Protein (roter scharfer Peak) mittels eines Elutionsgradienten (blaue Linie) eluiert wird. Auf diese Weise wird das Protein von jeglichen Verunreinigungen, die nicht an die Chromatographie-Säule binden und somit im Durchfluss (breiter roter Peak) eluieren, befreit. Die Proteinkristallisation ist grafisch als Einkristall des Hühnereiweiß-Lysozyms dargestellt. Das darauffolgende Röntgenbeugungsexperiment ist durch ein Diffraktionsmuster (Hühnereiweiß-Lysozym) dargestellt. Das Muster zeigt die Verteilung gebeugter Reflexe (schwarze Punkte) über den gesamten Detektorbereich (heller Hintergrund). Der Prozess der Strukturbestimmung wird durch die 3D-Struktur des Hühnereiweiß-Lysozyms repräsentiert.

Je nach Zielprotein ist die Beschaffung bzw. Herstellung der Kristallisationsprobe mehr oder weniger arbeitsintensiv. Im einfachsten Fall ist das Zielprotein kommerziell erhältlich, was häufig der Fall ist, wenn man die Wechselwirkungen eines (strukturell) bekannten Proteins mit verschiedenen Liganden untersucht. Falls jedoch das Forschungsziel die Strukturauflösung eines (strukturell) unbekannten oder kommerziell nicht erhältlichen Proteins ist, muss das Protein entweder direkt aus seiner natürlichen Quelle isoliert oder mittels molekularbiologischer Techniken hergestellt werden. Das Isolieren des Proteins aus seiner natürlichen Quelle geht im Hinblick auf die spätere Kristallisation mit vielen Schwierigkeiten einher [3]. Abhängig von der Natur des Proteins und dessen Ursprungsorganismus kann es sein, dass das Zielprotein aus physiologischen Gründen in nur geringen Mengen exprimiert wird (z.B. Stress-induzierte Proteine, die nur bei bestimmten Stimuli exprimiert werden). Dies hat zur Folge, dass große Mengen des Quellmaterials verarbeitet werden müssen, um eine ausreichende Menge an Protein für die Kristallisation zu erhalten (~ 2-10 mg). Des Weiteren führt die Anwesenheit von posttranslationalen Modifikationen (PTMs) und unterschiedlichen, aber dem Zielprotein strukturell sehr ähnlichen Isoformen zu einer inhomogenen Probenlösung, in der die Ausbildung von Einkristallen deutlich erschwert ist. Deshalb ist der molekularbiologische Ansatz die am häufigsten verwendete Technik zur Herstellung ausreichender Proteinmengen für die Kristallisation. Dabei wird das Gen des Zielproteins in ein Expressionssystem kloniert und anschließend in einer Wirtszelle, meistens ein konstruierter Stamm des Bakteriums *Escherichia coli* (*E. coli*), überexprimiert [4]. Bakterielle Expressionssysteme sind sehr robust und deshalb in der Lage, das Zielprotein in großen Mengen zu exprimieren. Außerdem modifizieren sie das Zielprotein nicht posttranslational, was Vor- und Nachteile hat. Einerseits verringert das Fehlen von PTMs die Inhomogenität der Probe, was die Kristallisation erleichtert, anderseits sind PTMs jedoch essentiell für die korrekte Faltung und Funktion vieler Proteine [5]. Sobald ausreichende Mengen an Protein produziert wurden, wird die Probe mittels verschiedener chromatographischer Verfahren, wie z.B. der hydrophoben Interaktions-, der Affinitäts-, der Ionenaustausch- und/oder der Größenausschlusschromatographie, bis zur Homogenität gereinigt (Entfernen von proteinogenen und nicht-proteinogenen Verunreinigungen). Anschließend wird die Proteinprobe für die folgende Kristallisation konzentriert. Das Ziel der Kristallisation ist das Wachstum von hochwertigen Einkristallen. Ein Proteinkristall ist eine dreidimensionale Anordnung von Proteinmolekülen, die aus sich in alle drei Raumrichtungen wiederholenden Einheiten, den sogenannten Einheitszellen, besteht (Abb. 2). Die Einheitszellen werden durch drei Vektoren bestimmter Längen ($\boldsymbol{a}$, $\boldsymbol{b}$, $\boldsymbol{c}$) und Winkel ($\boldsymbol{\alpha}$, $\boldsymbol{\beta}$, $\boldsymbol{\gamma}$) aufgespannt. Der gesamte Kristall kann durch die einfache

Translation (Verschiebung ohne Rotationen) der Einheitszelle in alle drei Raumrichtungen aufgebaut werden. Die Einheitszellen bestehen wiederum aus sogenannten asymmetrischen Einheiten. Die asymmetrische Einheit ist die kleinste Einheit einer Kristallstruktur, aus der mit Hilfe aller Symmetrieoperationen der Raumgruppe (= Translations- und Rotationssymmetrie) der gesamte Kristall erzeugt werden kann (Abb. 2). Der aus der Kristallisation resultierende Einkristall wird dann im Röntgenbeugungsexperiment gemessen. Die aus dem Experiment gesammelten Daten werden schließlich dazu verwendet, die Struktur des Zielproteins mittels komplexer mathematischer Methoden zu lösen. Das Endprodukt der Röntgenstrukturanalyse ist ein 3D-Modell des Zielproteins.

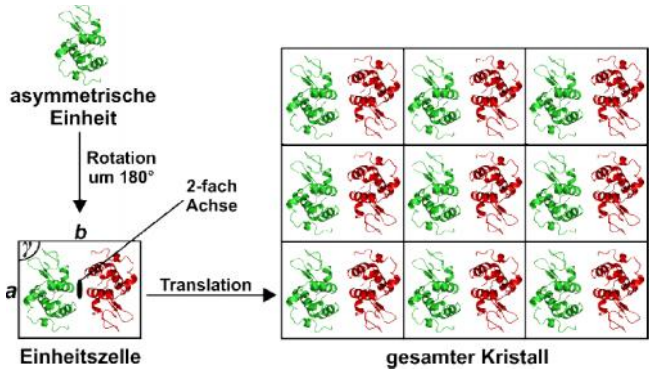

Abb. 2 Aufbau eines Kristalls. In diesem Beispiel besteht die Einheitszelle aus einem Proteinmolekül (Hühnereiweiß-Lysozym, grünes Molekül). Die Einheitszelle wird durch eine 180°-Rotation des Moleküls um eine 2-fache Rotationsachse erzeugt, wobei eine Kopie des Moleküls entsteht (Symmetrie-verwandtes Molekül, rotes Molekül). Die die Einheitszelle beschreibenden Parameter, nämlich die Zellkonstanten *a* und *b* und der dazugehörige eingeschlossene Winkel *γ*, sind eingezeichnet. Es wird darauf hingewiesen, dass es sich hier der Einfachheit halber nur um einen 2D-Kristall handelt. Der gesamte Kristall kann durch die Verschiebung der Einheitszelle im zweidimensionalen Raum erzeugt werden (2D in dieser Abbildung, aber 3D im echten Kristall).

Das Röntgenbeugungsexperiment

Für die Leser, die nicht mit der Röntgenstrukturanalyse vertraut sind, soll das Röntgenbeugungsexperiment in den nächsten Abschnitten kurz erklärt werden. Wozu brauchen wir Röntgenstrahlen? In jeder Form der Mikroskopie hängt die Auflösung oder, mit anderen Worten, die Detailtiefe des beobachteten Objekts von der Wellenlänge der verwendeten elektromagnetischen Strahlung ab. Röntgenstrahlen sind hochenergetische, elektromagnetische Wellen mit einem Wellenlängenbereich von 0.1 bis 100 Å. Aus diesem Grund eignen sich Röntgenstrahlen zur „Visualisierung“ von Proteinen auf atomarer Ebene, da die interatomaren Abstände ebenfalls in diesem Wellenbereich liegen, z.B. beträgt der atomare Abstand einer C-C-Bindung ~ 1.5 Å [6]. Während des Röntgenbeugungsexperiments werden die Röntgenstrahlen an den Elektronen des Proteinkristalls gestreut (Abb. 3a). Wenn sich Photonen durch einen Kristall bewegen, regen sie die Elektronen der Proteinatome zu einer erzwungenen Schwingung an, die dazu führt, dass die Elektronen selber Wellen emittieren. Während es zwischen den meisten dieser (gestreuten) Wellen zur destruktiven Interferenz kommt, interferieren nur einige wenige Wellen in bestimmten Richtungen (Winkeln) konstruktiv miteinander. Diese konstruktiven Interferenzen erzeugen sogenannte „Reflexe“, die mittels eines Detektors beobachtet werden. Die Wahrscheinlichkeit, eine Diffraktion (Röntgenbeugung) in einer bestimmten Richtung (bzw. bei einem bestimmten Winkel) zu beobachten, ist proportional zur Amplitude der resultierenden/gestreuten Welle (Strukturfaktor F). Dieses Phänomen der Röntgenbeugung an Kristallen wurde von Max T. von Laue (1879 - 1960) entdeckt, wofür er 1914 den Nobelpreis für Physik erhielt [7]. Von Laue bewies mit seinen Streuexperimenten nicht nur den Wellencharakter von Röntgenstrahlen, sondern auch, dass die Atome in einem Kristall in einem Raumgitter angeordnet sind. Er war auch der Erste, der die Bedingungen, die zu einer detektierbaren Streuung führen, mathematisch erklärte [8]. Im Jahre 1913, basierend auf der Arbeit von von Laue, war es William L. Bragg (1890 - 1971), der eine vereinfachte Gleichung zur Beschreibung der Bedingungen, die beim Röntgenbeugungsexperiment zu einer konstruktiven Interferenz von Wellen führt, formulierte. Diese berühmte und nicht wegzudenkende Gleichung ist als Bragg-Bedingung bekannt und lautet [9]:

$$n\lambda = 2d \sin\theta$$

Um die Streuung von Röntgenstrahlung zu beschreiben, führte Bragg hypothetische Gitterebenen (Bragg-Ebenen) ein, die den Kristall schneiden. Diese Ebenen kann man sich als imaginäre Spiegelebenen vorstellen, die die ankommenden Röntgenstrahlen reflektieren. Auf den Ebenen befinden sich die Atome (inklusive streuender

Elektronen) des Kristalls, wobei die einzelnen Gitterebenen durch den interplanaren Abstand *d* voneinander getrennt sind (Abb. 3b). Treffen also Röntgenstrahlen mit der Wellenlänge λ und einem Einfallswinkel von θ auf äquivalente Gitterebenen, dann treten die reflektierten Strahlen im selben Winkel zu den Ebenen aus, wobei die reflektierten Wellen nur dann konstruktiv miteinander interferieren (= Entstehung detektierbarer Reflexe), wenn der Gangunterschied zwischen ihnen ein ganzzahliges Vielfaches *n* der Wellenlänge ist. Alle einfallenden Röntgenstrahlen, die die Bragg-Bedingung nicht erfüllen, werden nicht in Phase gestreut (= destruktive Interferenz) und somit kommt es in der jeweiligen Richtung bzw. im jeweiligen Winkel (abhängig vom Einfallswinkel θ) zu keinem messbaren Reflex.

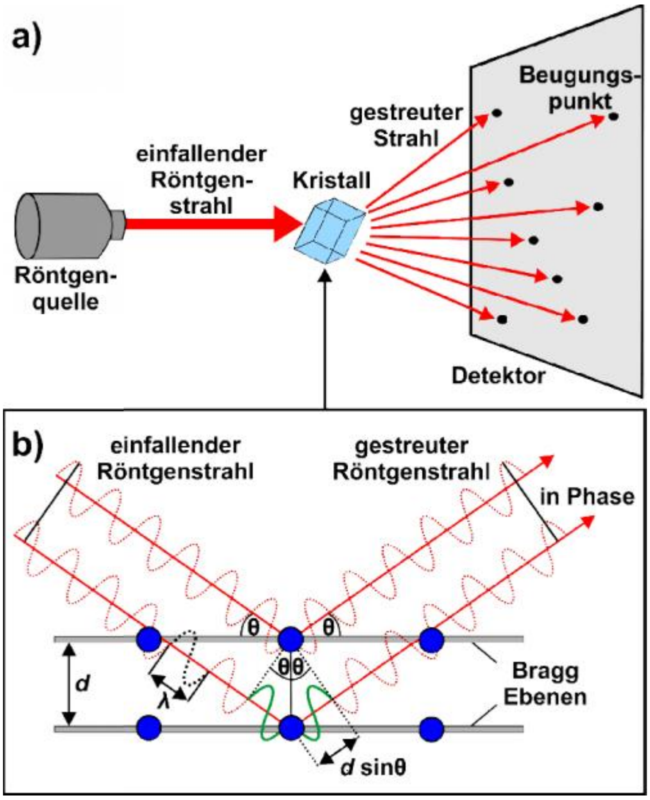

Abb. 3 Röntgenbeugungsexperiment. a) Grundschema eines Röntgenbeugungsexperiments. Ein einfallender Röntgenstrahl tritt in den Kristall ein, wobei die gestreuten Strahlen das Diffraktionsmuster (siehe Beugungspunkte) bilden, welches vom Detektor aufgenommen wird. b) Beugung laut Bragg-Bedingung. Der Fall der konstruktiven Interferenz (= in Phase) ist abgebildet. Konstruktive Interferenz kommt nur zustande, wenn der Gangunterschied (*d* sinθ) zwischen den gestreuten Wellen (rot gepunktete Wellen) ein ganzzahliges Vielfaches der Wellenlänge λ ist. Der Gangunterschied im gezeigten Bild beträgt genau 1 λ (*n* = 1, dargestellt als grüne durchgehende Welle). Dies führt dazu, dass sich die Amplituden der reflektierten Wellen addieren und somit ein messbares Signal auf dem Detektor erzeugen. Im Fall, dass der Gangunterschied kein ganzzahliges Vielfaches von λ ist, interferieren die gebeugten Wellen destruktiv miteinander, was zu keiner messbaren Reflexion führt. Die zwei abgebildeten grauen Balken stellen Bragg-Ebenen dar, die durch den interplanaren Abstand *d* voneinander getrennt sind. Auf beiden Ebenen liegen drei Gitterpunkte (blaue Kugeln), die im Fall der Proteinkristallisation Atome des Proteins darstellen.

Die Engpässe in der makromolekularen Röntgenstrukturanalyse

Es gibt eine Reihe von experimentellen Schwierigkeiten in der makromolekularen Röntgenstrukturanalyse (z.B. die Präparation ausreichender Mengen an Zielprotein in hochreiner Form), wobei jedoch die größten experimentellen Hürden der Kristallisationsprozess selbst (= Wachstum von Kristallen, die Röntgenstrahlen bei hoher Auflösung streuen) und das Lösen des sogenannten ‚Phasenproblems‘ sind.

Das ‚Phasenproblem‘ und dessen Lösung

Jeder gestreute Röntgenstrahl, der den Detektor während des Beugungsexperiments erreicht, besitzt eine bestimmte Amplitude (Stärke des gestreuten Röntgenstrahls, siehe Abb. 3b) und Phasenwinkel (ϕ, der Winkel, der die relative Verschiebung zwischen den Wellen beschreibt). Um die Streuung mathematisch zu beschreiben, verwenden Kristallographen eine Größe, die Strukturfaktor (F) genannt wird und sowohl die Amplitude als auch die Phase jedes

gemessenen Reflexes beinhaltet (Abb. 4) [6]. Der Strukturfaktor selbst ist eine komplexe Zahl, die aus einem Real- und Imaginärteil besteht, und eine Summierung der Beiträge aller Atome in der Einheitszelle zum gemessenen Reflex darstellt (Abb. 4b). Jedes Atom besitzt seinen eigenen atomaren Streufaktor (f_i, i = Atom), der ein Maß für die Streustärke eines einzelnen Atoms ist. Dieser individuelle Streufaktor (f_i) hängt von der Art des Atoms (Anzahl der Elektronen), dessen Bewegung relativ zum einfallenden Röntgenstrahl, dem Streuwinkel θ und zu einem gewissen Maße auch von der Wellenlänge λ des einfallenden Röntgenstrahls ab. Für die Lösung einer Proteinstruktur mittels mathematischer Methoden benötigt man sowohl die Amplitude als auch die Phase jedes einzelnen gestreuten Röntgenstrahls. Die Amplitude eines Streufaktors kann experimentell bestimmt werden, da die gemessene Intensität (I) des gestreuten Röntgenstrahls proportional zum Quadrat der Amplitude ist ($F^2 \sim I$). Leider ist der Detektor nicht in der Lage den Phasenwinkel (ϕ) eines Reflexes zu messen, der einen Großteil und vor allem auch die wichtigsten strukturellen Informationen (d.h. Informationen über die Atompositionen im Kristall) beinhaltet. Dieses Problem wird als ‚Phasenproblem' bezeichnet.

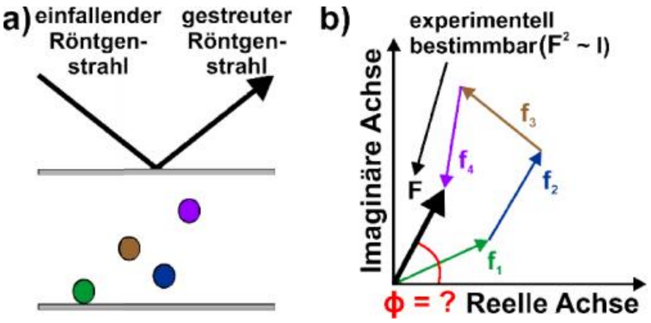

Abb. 4 Das ‚Phasenproblem'. a) Schema einer Röntgenbeugung. Zwei Bragg-Ebenen (graue Balken) sind zusammen mit vier Atomen (farbige Kugeln), die nicht auf den Ebenen liegen und somit phasenverschoben sind (nur Atome, die auf den Ebenen liegen streuen in Phase), abgebildet. Jedes Atom (auf oder in der Nähe der Bragg-Ebenen) trägt mit einem relativen Phasenwinkel ϕ, der durch den relativen Abstand der Atome zu den Ebenen bestimmt wird, zu jedem einzelnen, gestreuten Reflex bei. b) Der Beitrag jedes einzelnen Atoms zum beobachteten (gemessenen) Gesamtreflex (schwarzer Vektor) ist anhand von Vektoren (komplexe Zahlen) angezeigt, wobei die Farben der Vektoren mit den Atomfarben in Abbildung a) übereinstimmen. Die Abbildung verdeutlicht, dass der gemessene Reflex (F) eine bestimmte Amplitude (Länge des Vektors) und Phase (Winkel) besitzt, die durch die Summe der Streubeiträge aller individuellen Streufaktoren (in diesem Fall vier Atome, f_1-f_4) bestimmt wird, wobei jeder einzelne Streuer (die vier Atome) eine eigene Phase und Amplitude besitzen. Die Amplitude kann experimentell bestimmt werden, da sie proportional zur Quadratwurzel der gemessenen Intensität (I) ist. Leider verliert man während des Experiments jegliche Phaseninformation (angezeigt durch ein rotes Fragezeichen), da der Detektor nur Intensitäten messen kann. Deshalb können nur elektronische Eigenschaften der Atome (d.h. Streustärke) und nicht ihre strukturellen Eigenschaften (d.h. Phasenwinkel), die ihre Positionen im Kristallgitter beschreiben, experimentell bestimmt werden.

Es gibt einige Methoden, um die ‚verlorenen' Phaseninformationen wiederherzustellen, z.B. durch die Methode des Molekularen Ersatzes (molecular replacement, MR), des Isomorphen Ersatzes (single/multiple isomorphous replacement, SIR/MIR) und der anomalen Dispersion bei einer oder mehreren Wellenlängen (single/multiple wavelength anomalous dispersion, SAD/MAD) [10, 11, 12]. MR ist die einfachste Methode zur Lösung des ‚Phasenproblems', da anfängliche Phasen von einem strukturell verwandten Protein (laut Faustregel ist eine Sequenzidentität von ~ 20-30% erforderlich), dessen Struktur (und somit Phasen) bekannt sind, abgleitet werden [13]. Die Methode basiert auf mathematischen Algorithmen, die versuchen, das strukturelle Homolog (das sogenannte Suchmodell) in einem Kristallgitter so zu positionieren, dass es mit den experimentellen Daten so weit wie möglich übereinstimmt. Sobald das Homolog richtig positioniert wurde, werden dessen berechnete Phasen verwendet, um die Phasen der unbekannten Struktur zu bestimmen. Die anderen zwei Methoden funktionieren unabhängig von homologen Strukturen, jedoch ist die Phasenbestimmung dann mit einem zusätzlichen experimentellen Aufwand verbunden, weshalb diese Methoden als experimentelle Phasenbestimmung bezeichnet werden. Für die SIR/-MIR-Methode benötigt man einen Kristall des nativen Zielproteins und mindestens einen (= SIR, bei mehreren ist es MIR) Kristall eines isomorphen Derivats. Die Proteine der isomorphen Derivate besitzen schwere Atome (z.B. Metalle oder Schwermetalle) in ihrer Struktur. Wie der Name bereits verrät, müssen die Derivat-Kristalle ausreichend isomorph zum nativen Kristall sein, d.h. das Einfügen der schweren Atome darf zu keinen größeren Veränderungen in den Zelldimensionen des Derivat-Kristalls führen (im Vergleich zu denen des nativen Kristalls). Laut einer Faustregel sind Veränderungen der Zelldimensionen von $d_{min}/4$ noch akzeptabel (d_{min} = Auflösungsgrenze). Angenommen, man hat einen Datensatz mit einer Auflösung von 2.5 Å, dann kann die Methode des Isomorphen Ersatzes selbst bei Schwankungen zwischen den Einheitszelldimensionen des nativen und Derivat-Kristalls von bis zu 0.6 Å noch brauchbare Phasen liefern [14]. Prozentual gesehen kann jedoch ein Unterschied in

der Einheitszelle von bereits ~ 0.5 - 1.0 % die Methode des isomorphen Ersatzes zum Scheitern bringen. Die Schweratome werden mittels Co-Kristallisation (Proteinlösung und die Lösung mit den schweren Atomen wird vor der Kristallisation gemischt) oder ‚Soaking‘ (ein bereits gewachsener Proteinkristall wird in eine Lösung mit schweren Atomen getränkt, wodurch die Schweratome in die Proteinstruktur diffundieren können) in die Struktur eingeführt, wo sie an verschiedene Aminosäurereste binden. Die eingeführten Schweratome tragen auf Grund ihrer großen Elektronenzahl stark zur Beugung der Röntgenstrahlen bei, da der Streubeitrag eines Atoms proportional zum Quadrat der Anzahl seiner Elektronen ist. Der Streubeitrag der Schweratome ist immens im Vergleich zum Beitrag der in den Proteinen normalerweise auftretenden leichten Atome (Kohlenstoff, Stickstoff und Sauerstoff). Folglich kann der durch die Schweratome verursachte Unterschied in der Streuintensität einfach bestimmt werden. Diese Unterschiede in den Streuintensitäten (zwischen dem nativen Kristall und dem Derivat-Kristall) spiegeln hauptsächlich die Streubeiträge der schweren Atome wider, was ausgenutzt wird, um die Positionen der schweren Atome zu bestimmen. Die Positionen der Schweratome werden zusammen mit den experimentell bestimmten Strukturfaktoramplituden des nativen Proteins und dessen Derivat(e) verwendet, um z.B. geometrisch mittels der sogenannten Harker-Konstruktion die Phasen abzuleiten [12]. Da das Wachstum von Derivat-Kristallen mit einer für die SIR/MIR-Methode ausreichend hohen Isomorphie zu einem schwierigen Unterfangen werden kann, verwendet man heutzutage bevorzugt die SAD/MAD-Methode. Diese Methode erfordert die Einführung von anomalen Streuern in die Proteinstruktur. Anomale Streuer sind Atome, die eine Absorptionskante innerhalb des Wellenlängenbereiches besitzen, die normalerweise für die Röntgenstrukturanalyse verwendet wird (~ 0.7 - 2.5 Å) [15]. Ähnlich der SIR/MIR-Methode basiert diese Methode auch auf der Veränderung der Streuintensität. In diesem Fall wird die Veränderung der Streuintensität jedoch durch die Wellenlängen-abhängige Änderung der Streufaktoren der anomalen Streuer verursacht. Die Absorption von Röntgenstrahlung an (oder in der Nähe) der Absorptionskante eines anomalen Streuers führt zu einer Phasenverschiebung, die sich deutlich von der unterscheidet, die man bei einer üblichen elastischen Streuung beobachtet. Die MAD-Methode benötigt deshalb Daten von nur einem Kristall, jedoch bei unterschiedlichen Wellenlängen, um die anomale Dispersion der Röntgenstrahlen durch die anomalen Streuer ausnutzen zu können. Es werden üblicherweise Datensätze bei drei verschiedenen Wellenlängen aufgenommen. Ein Datensatz wird direkt an der Absorptionskante, d.h. an der Spitze der Absorptionskurve des anomalen Streuers gemessen (= größtes anomales Signal). Ein weiterer Datensatz wird am Wendepunkt der Absorptionskurve aufgenommen (= hohe Dispersion), wohingegen der letzte Datensatz bei einer Wellenlänge, die sich relativ weit entfernt von den ersten beiden Wellenlängen befindet, gemessen wird (niedrige Dispersion). Auf diese Weise erhält man Datensätze, die sich deutlich in ihrer Dispersion unterscheiden und somit einen maximalen Wellenlängen-abhängigen Unterschied in der Streukraft zeigen. Im Fall der SAD-Methode werden Daten bei nur einer Wellenlänge aufgenommen, nämlich bei der Wellenlänge der Absorptionskante (= Spitze der Absorptionskurve, maximale Absorption des anomalen Streuers). Die Phasen werden dann durch die (durch anomale Streuer verursachten) Unterschiede in der Streuintensität zwischen den Daten, die bei verschiedenen Wellenlängen aufgenommen wurden (= unterschiedliche Streukraft), abgeleitet. Bei der MAD-Methode muss sichergestellt werden, dass der eine verwendete Kristall während des Experiments nicht durch Strahlenschäden zerstört wird, da der Kristall die Strahlendosis mehrerer Messungen (bei verschiedenen Wellenlängen) überstehen muss. Die Verwendung von Schweratomen bzw. anomalen Streuern, die auch in POMs häufig vertreten sind, stellen eine etablierte Methode zur Lösung des ‚Phasenproblems‘ dar.

Die Proteinkristallisation

Aufgrund der raschen Entwicklung von Synchrotron-Einrichtungen (Röntgenstrahlquelle), die Röntgenstrahlen mit hohem Strahlenfluss (hochintensive Photonenstrahlen), hoher Brillanz (hochkollimierte Strahlenbündel) und einstellbarer Wellenlänge (wichtig für die MAD-Methode) zur Verfügung stellen, und der Weiterentwicklung von Phasing-Programmen, stellt das ‚Phasenproblem‘ heutzutage nur ein kleineres Problem dar. Deshalb ist das Wachstum von qualitativ hochwertigen Einkristallen der limitierende Schritt in der makromolekularen Kristallographie [16]. Bei der Proteinkristallisation handelt es sich hauptsächlich um ein ‚Trial-and-Error‘-Verfahren, da die der Methode zugrundeliegende Physik hoch komplex ist und vom Zusammenspiel vieler Faktoren (z.B. Protein- und Fällungsmittelkonzentration, pH-Wert, Temperatur, Ionenstärke, etc.) abhängt. All dies trägt zur generellen Unberechenbarkeit des Kristallisationsprozesses bei [17]. Der Grund dafür ist, dass die Entstehung eines Proteineinkristalls einen von Natur aus unwahrscheinlichen Prozess darstellt, da Proteine teilweise sehr flexibel und dynamisch sein müssen, um ihre Funktion auszuüben. Zudem ist die Anzahl der Wechselwirkungen, die die Proteine in einem Kristall zusammenhalten, sehr gering und von schwacher, nicht-kovalenter Natur (Ionen-, Dipol-, van der Waals-Wechselwirkungen und Wasserstoffbrücken) [6]. Hinzu kommt, dass die wenigen intermolekularen Kontakte

zwischen den Proteinmolekülen in einem Kristall sehr spezifisch sind und von den Oberflächeneigenschaften des Proteins abhängen. Dies bedeutet, dass die Verteilung geladener und polarer Aminosäuren auf der Proteinoberfläche, die dem Lösungsmittel exponiert ist, die Kristallisierbarkeit eines Proteins mitbestimmt. Dies ist auch der Grund, warum einige Proteine dem Anschein nach von Natur aus nicht kristallisierbar sind, da sich ihre Oberflächeneigenschaften nicht mit der Ausbildung eines Kristallgitters vereinbaren lassen. Wozu brauchen wir aber überhaupt Kristalle? Die Röntgenstrukturanalyse hängt von der Auswertung und Berechnung struktureller Informationen, die durch die Beugung von Röntgenstrahlen am Kristall des Zielproteins generiert und von einem Detektor aufgenommen werden, ab. Es werden jedoch nur ~ 1% der eingestrahlten Röntgenstrahlen durch die Elektronen des Proteins gestreut. Das Streusignal eines einzigen Proteinmoleküls wäre nicht messbar, deshalb benötigt man Kristalle (geordnete, dreidimensionale Anordnungen von Proteinmolekülen), um die Intensität (Signal) der gestreuten Röntgenstrahlen zu amplifizieren. Damit Einkristalle wachsen können, muss eine hochreine Proteinlösung zur Übersättigung gebracht werden, um die kinetische Energie-Barriere für die Bildung von Kristallkeimen zu überwinden (Phasentrennung) [18]. Die Keimbildung [19] ist der wichtigste Schritt in der Proteinkristallisation und stellt einen Phasenübergang erster Ordnung von einem ungeordneten in einen geordneten Zustand dar [20]. Ein kritischer Keim von bestimmter Größe (kritische Größe) bildet den hochenergetischen Übergangszustand des Keimbildungsprozesses. In diesem Stadium kann der Keim entweder wieder zerfallen oder im gewünschten Fall zu einem Kristall weiterwachsen (Abb. 5). Der letztere Vorgang benötigt einen Keim mit einem (noch) größeren Radius und somit einen höheren Grad an Übersättigung.

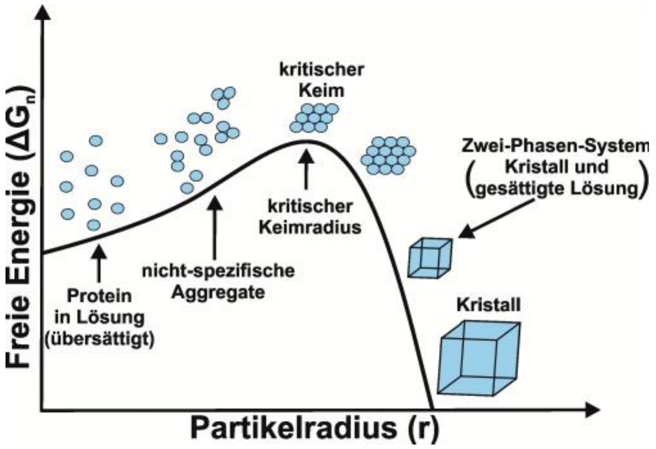

Abb. 5 Die Änderung der Gibbs-Energie (ΔG_n) während der Kristallisation in Abhängigkeit vom Keimradius r. Eine kinetische Energiebarriere (Kurvenspitze) muss überwunden werden, um einen Keim, dessen Radius über der kritischen Größe liegt, zu erhalten, damit die antizipierte Phasentrennung (gesättigte Lösung und fester Kristall) eingeleitet wird. Bei ausreichender Übersättigung kommt es in der Lösung zu Kollisionen zwischen den Proteinmolekülen, die zur Ausbildung von nicht-spezifischen Aggregaten führen. Einige dieser Aggregate erreichen mit der Zeit eine bestimmte Größe, wobei sich immer mehr Proteinmoleküle an die Oberfläche dieser Aggregate anlagern, was letztendlich zur Ausbildung kritischer Keime führt. Sobald ein Keim die kritische Größe erreicht hat, ist das weitere Wachstum auch energetisch bevorzugt, da der Energiegewinn durch die Enthalpie, die durch die Adsorption von Proteinmolekülen an die Oberfläche des Keims freigesetzt wird, die entropischen Verluste während des Kristallwachstums kompensiert. Ab diesem Punkt kann das System in Richtung Einkristall, der für das Röntgenbeugungsexperiment geeignet ist, fortschreiten. Die kleinen blauen Kugeln stellen Proteinmoleküle dar.

Eine zu hohe Proteinkonzentration fördert die Entstehung ungeordneter Makromolekül-Aggregate (Niederschlag), wohingegen eine zu niedrige Proteinkonzentration keine Übersättigung der Lösung herbeiführt. Deshalb ist es wichtig, ein passendes Maß an Übersättigung zu erzielen, um die Keimbildung zu ermöglichen. Da eine übersättigte Proteinlösung mit Proteinmolekülen überlaufen ist, kollidieren diese häufiger miteinander (als in einer weniger gesättigten Lösung), wobei es unter bestimmten Umständen zu Kollisionen in günstiger Orientierung der Proteinmoleküle kommen kann, die die Bildung von spezifischen Protein-Protein-Kontakten ermöglichen. Auf diese Weise entstehen die notwendigen Kristallisationskeime, die dementsprechend weiterwachsen, je mehr Proteinmoleküle an ihre Oberfläche binden. Dieser Prozess ist mit dem Übergang von Proteinmolekülen aus der flüssigen (Lösung) in die feste Phase (Keim) verbunden, weshalb sich mit fortschreitendem Keim- bzw. Kristallwachstum die Proteinkonzentration in der Kristallisationslösung verringert. Die Kristallisationslösung wird in einen thermodynamisch metastabilen Zustand gebracht, wo die geordnete Aggregation von Proteinmolekülen begünstigt wird [21]. Die notwendige Übersättigung der Kristallisationslösung wird durch die Zugabe eines Fällungsmittels erreicht. Generell senken Fällungsmittel die Löslichkeit von Proteinen, indem sie den Proteinen die Wassermoleküle aus ihrer Hydratationshülle entziehen, was dazu führt, dass die Wechselwirkungen zwischen den

Proteinen stärker als die Interaktionen mit dem Lösungsmittel werden [22]. Abb. 6 zeigt das Phasendiagramm einer üblichen Kristallisation, in dem unter anderem die Regionen der Keimbildung und des Kristallwachstums in Abhängigkeit von der Protein- und Fällungsmittelkonzentration dargestellt sind. Der Kristallisationsprozess hängt nicht nur von der passenden Menge an Protein und/oder Fällungsmittel, sondern auch von anderen Faktoren, wie z.B. der Proteinreinheit/-homogenität, pH-Wert, Temperatur, Ionenstärke, etc. ab. Das Kristallisationsdiagramm vereint die Informationen über thermodynamisch definierte Phasenbeziehungen und verschiedene kinetische Vorgänge (Keimbildung und Wachstum). Die Kristallisation ist thermodynamisch nur möglich, wenn eine metastabile Proteinlösung in ein Äquilibrium übergehen kann, indem sie sich in eine stabile proteinreiche Phase (z.B. Kristall) und eine gesättigte Lösung (Wachstumslösung) aufteilt [22]. Dabei bestimmen kinetische Prozesse, ob die thermodynamisch mögliche Ausbildung eines Kristalls auch tatsächlich in die Realität umgesetzt wird. Allgemein betrachtet sind die kinetischen Vorgänge deutlich schwieriger zu kontrollieren als die thermodynamischen Bedingungen, die relativ einfach eingestellt werden können (z.B. durch die passende Zusammensetzung der Kristallisationslösung, Temperatur, etc.). Deshalb ist es die Aufgabe des Proteinkristallographen, (thermodynamisch und kinetisch passende) Bedingungen zu erarbeiten, die das System in Regionen führt, die die Kristallisation ermöglichen und fördern [23].

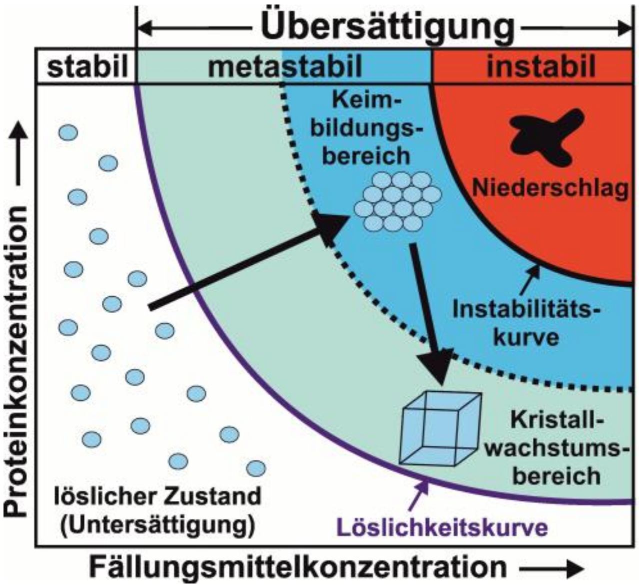

Abb. 6 Phasendiagramm der Proteinkristallisation. Die Löslichkeitskurve (dunkelblaue Linie) teilt das Diagramm in einen untersättigten (weißer Hintergrund) und übersättigten (hellblau, blauer und roter Hintergrund) Bereich. Der untersättigte Bereich ist eine einphasige und stabile Lösung, in der die Proteinmoleküle (dargestellt als hellblaue Kugeln) in Lösung bleiben. Mit zunehmender Sättigung wird die Löslichkeitskurve überschritten und die Lösung wird übersättigt. Die übersättigte Zone ist in einen metastabilen (hellblauer und blauer Hintergrund) und instabilen (roter Hintergrund) Bereich unterteilt. Der metastabile Bereich besteht aus dem Kristallwachstumsbereich (hellblauer Hintergrund) und dem Keimbildungsbereich (blauer Hintergrund). Im Kristallwachstumsbereich bilden sich nur transiente (flüchtige) Keime, die die kritische Größe nicht erreichen. Wenn der Grad der Übersättigung steigt (angedeutet durch den großen schwarzen Pfeil, der vom untersättigten Bereich ausgeht), erreicht man die Keimbildungszone, wo die Keime die notwendige kritische Größe erreichen und sich stabilisieren können (Keimbildung). Mit zunehmender Größe des Keims und der Bildung von Kristallen sinkt die Proteinkonzentration in der Lösung und das System kehrt in den Kristallwachstumsbereich zurück (angedeutet durch den großen schwarzen Pfeil, der nach unten in die Richtung des Kristallwachstumsbereiches zeigt). In diesem Bereich können die entstandenen Kristalle bei geringerer Übersättigung weiterwachsen. Bei zu hoher Übersättigung erreicht man den instabilen Bereich (roter Hintergrund), wo die Proteine amorph präzipitieren.

Es gibt einige Methoden, Kristalle wachsen zu lassen, wozu die Batch-Kristallisation [24], Mikrodialyse [25], Liquid-Liquid-Free-Interface-Diffusion [26] und die Dampfdiffusion zählen [27]. Da letztere Methode die am häufigsten verwendete ist, wollen wir diese im Folgenden kurz beschreiben. Die Dampfdiffusionsmethode kann man in zwei Konfigurationen durchführen, im sogenannten hanging-drop- oder sitting-drop-Format (Abb. 7). Der Mechanismus beider Konfigurationen ist identisch, da in beiden Fällen eine Proteinlösung mit einer Kristallisationslösung, die das Fällungsmittel (und andere Komponenten wie z.B. Additive) beinhaltet, zu einem Kristallisationstropfen gemischt wird. Beim hanging-drop-Ansatz werden wenige Mikroliter (üblicherweise 0.1 - 5 µL) der Protein- als auch der Kristallisationslösung auf ein silikonisiertes Abdeckplättchen platziert, das ein Näpfchen abdeckt, in der sich nur Kristallisationslösung (0.05 - 1 mL) befindet, das sogenannte Reservoir (Abb. 7a). Bei dem sitting-drop-Ansatz werden die Protein- und Kristallisationslösung in der Vertiefung einer kleinen

Plattform gemischt, die sich innerhalb des Reservoirs befindet (Abb. 7b). In beiden Fällen werden die Näpfchen mit Schmierfett abgedichtet, um eine Verdampfung zu verhindern und das System vor äußeren Einflüssen zu schützen. Da das Reservoir eine höhere Konzentration an Fällungsmittel enthält als der Kristallisationstropfen (der Kristallisationstropfen wird durch die Proteinlösung verdünnt), diffundiert Wasserdampf aus dem Kristallisationstropfen in das Reservoir, damit sich ein chemisches Gleichgewicht zwischen dem Tropfen und dem Reservoir einstellt. Die Dampfdiffusion führt dazu, dass der Kristallisationstropfen schrumpft und somit sowohl die Protein- als auch Fällungsmittelkonzentration im Tropfen langsam ansteigen, bis die Löslichkeitsgrenze des Proteins überschritten wird. Das Überschreiten der Löslichkeitsgrenze markiert den Startpunkt für den Kristallisationsvorgang, in dessen Verlauf es zur Keimbildung, Phasentrennung und im besten Fall zum Kristallwachstum kommt (siehe Abb. 6).

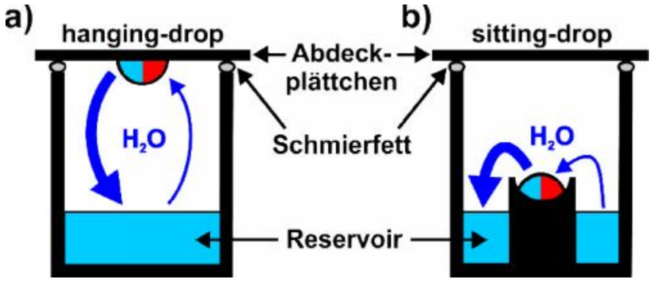

Abb. 7 Dampfdiffusionsmethode. a) Hanging-drop- und b) sitting-drop-Konfiguration. Die Näpfchen werden mit den Abdeckplättchen zugedeckt und mittels Schmierfett abgedichtet. Der Kristallisationstropfen besteht aus der Kristallisationslösung, die das Fällungsmittel enthält (hellblauer Teil des Tropfens), und der Proteinlösung (roter Teil des Tropfens). Die Gleichgewichtseinstellung über die Dampfdiffusion ist durch die blauen Pfeile dargestellt.

Die Verwendung von Additiven in der Proteinkristallisation

Die Kristallisation ist ein thermodynamisch und kinetisch äußerst komplexer Vorgang, der noch nicht vollständig geklärt ist. Häufig scheitert die Kristallisation und somit das Wachstum qualitativ hochwertiger Einkristalle, indem man nur die üblichen Parameter wie z.B. Protein- und/oder Fällungsmittelkonzentration, pH-Wert, Temperatur, etc. variiert. Deshalb wurden einfache Methoden entwickelt, um die Qualität von anfänglich gewachsenen Proteinkristallen zu verbessern oder allgemein die Kristallisierbarkeit von Proteinen zu verbessern. Die Verwendung von Additiven ist eine bewährte Methode in der Proteinkristallisation, um die Kristallisation eines Proteins zu verbessern oder gar erst zu ermöglichen. Das Additiv wird meistens der Kristallisationslösung hinzugefügt (Co-Kristallisation), kann aber auch Teil der Proteinlösung sein. Additive sind üblicherweise Verbindungen, meist kleine Moleküle oder Ionen, die die Fähigkeit besitzen, die Kristallisationswahrscheinlichkeit eines Proteins zu erhöhen, indem sie das Kristallgitter stabilisieren oder seinen Aufbau erst ermöglichen. Es gibt zwei Arten von Additiven, einerseits Verbindungen, die aufgrund rationaler Grundlagen verwendet werden, und andererseits Verbindungen, die Eigenschaften besitzen, mit welchen sie auf nicht immer erklärbare Art und Weise die Kristallisation des Proteins positiv beeinflussen könnten. Die erstgenannte Gruppe an Verbindungen sind Moleküle, die aus physiologisch relevanten Gründen die physikochemischen Eigenschaften und/oder die Konformation des Proteins beeinflussen, wie z.B. Substrate, Inhibitoren, Cofaktoren oder andere Effektor-Moleküle. Beispielsweise kann ein Apoprotein (z.B. ein Enzym in Abwesenheit seines Cofaktors) deutlich weniger zur Kristallisation neigen als das entsprechende Holoprotein (Enzym mit gebundenem Cofaktor) oder eine andere Liganden-gebundene Form des Proteins. Deshalb können Cofaktoren und/oder Liganden die Kristallisierbarkeit eines (Apo-)Proteins enorm erhöhen. Ein Grund dafür könnte die strukturelle Rigidität von Holoproteinen im Vergleich zu Apoproteinen sein, da generell die Bindung von Cofaktoren (oder anderen Effektoren) zu einer Verringerung der Beweglichkeit und Dynamik von Proteinen/Enzymen führt. Die andere Gruppe von Additiven umfasst Moleküle/Ionen, die das Kristallisationsverhalten von Proteinen auf unterschiedliche Art und Weise beeinflussen, beispielsweise Moleküle, die die Wechselwirkungen zwischen dem Makromolekül und dem Lösungsmittel beeinflussen, wie z.B. chaotrope und kosmotrope Verbindungen [28]. Moleküle, die die Löslichkeitseigenschaften von Makromolekülen beeinflussen, wie z.B. Detergentien, die besonders wichtig für die Kristallisation von Membranproteinen sind [29], oder Ionen, die bestimmte Proteinkonformationen, die möglicherweise besser kristallisierbar sind, stabilisieren, können auch positive Auswirkungen auf das Kristallisationsergebnis haben [30]. Die vielversprechendsten Moleküle dieser Gruppe sind diejenigen, die in der Lage sind, reversible ‚Crosslinks' (= ‚Verbrückungen') innerhalb des Proteins (intramolekular) oder zwischen verschiedenen Proteinmolekülen (intermolekular) zu schaffen [31]. Diese

‚Crosslinks' basieren auf Wasserstoffbrückenbindungen, elektrostatischen oder hydrophoben Wechselwirkungen, weshalb das Wort ‚Crosslinks' in Anführungszeichen steht, da es sich hierbei nicht um kovalente Bindungen handelt. Diese Art von Wechselwirkungen stabilisiert nicht nur das Kristallgitter, sondern führt auch zu neuen Kristallkontakten (Protein-Protein-Kontakten). Multivalente und geladene Ionen oder Moleküle werden häufig als ‚verbrückende' (‚crosslinking') Additive verwendet. Die Verwendung der meisten Additive ist jedoch auf bestimmte Proteine und/oder Kristallisationsbedingungen beschränkt, was sie während der Kristallisation vieler anderer Proteine wirkungslos macht. Deshalb besteht noch immer der Bedarf an neuen Additiven, die in der Lage sind, die Kristallisation einer größeren Anzahl an Proteinen zu fördern, insbesondere die Kristallisation von speziellen ‚nicht-kristallisierbaren' Proteinen, und gleichzeitig in einem breiten Spektrum an Kristallisationsbedingungen anwendbar sind. In dieser Hinsicht stellen Polyoxometallate (POMs) [32] vielversprechende Kandidaten als Additive in der Proteinkristallisation dar. Aufgrund ihrer einzigartigen Strukturen und Eigenschaften wie z.B. der hohen Löslichkeit, der thermischen Stabilität und ihrer hohen Ladung scheinen POMs wie geschaffen dafür zu sein mit Proteinen (auf eine ihre Kristallisation fördernde Art und Weise) zu wechselwirken.

Die Stoffklasse der Polyoxometallate

Polyoxometallate (POMs) sind eine Klasse anorganischer Cluster, die aus Sauerstoff- und Übergangsmetallatomen (Mo, W, V, Nb, Ta) bestehen, wobei sich die Metallionen meistens in ihrem höchsten Oxidationszustand befinden. POMs bieten eine überwältigende Vielfalt hinsichtlich ihrer Größe und Struktur und besitzen herausragende Eigenschaften und Funktionen [32]. POMs waren Gegenstand zahlreicher Studien und sind daher eine gut charakterisierte Verbindungsklasse mit einem breiten Anwendungsspektrum, das die Katalyse [33], Nanowissenschaften [34], Medizin [35, 36, 37, 38, 39] und seit kurzem die makromolekulare Kristallographie einschließt [40, 41]. POMs werden in Isopolyanionen $[M_mO_y]^{n-}$ und Heteropolyanionen $[X_xM_mO_y]^{n-}$ unterteilt. M ist das sogenannte Addenda- oder Polyatom (frühe Übergangsmetallionen), meistens Mo^{6+}, W^{6+} oder V^{5+}. X ist das Heteroatom, welches entweder ein Hauptgruppen- oder Übergangsmetall ist. Als Heteroatom können ca. 50 Elemente (Metalle und Nichtmetalle) des Periodensystems dienen. Die Wahl des Addenda-Atoms ist dagegen deutlich eingeschränkter. Das Addenda-Metall muss ein günstiges Ladung-Radius-Verhältnis und ein leeres d-Orbital besitzen, wobei Letzteres für die Ausbildung der M-O-Bindungen mit den Sauerstoffatomen mittels dπ-pπ-Überlappung (Elektronentransfer aus einem gefüllten p-Orbitals des Sauerstoffatoms in das leere d-Orbital des Addenda-Metalls) benötigt wird. POMs sind aus $\{MO_y\}$-Einheiten (y = 4-7) aufgebaut, wobei y die Koordinationszahl des Addenda-Atoms M angibt. Diese $\{MO_y\}$-Einheiten können auf verschiedene Arten miteinander gepackt werden (Selbstassemblierung), um POMs mit unterschiedlichen Formen und Größen zu bilden. Die verzerrt oktaedrische $\{MO_6\}$-Einheit ist der geläufigste Grundbaustein von POMs. Die Selbstassemblierung erfolgt hauptsächlich dadurch, dass sich $\{MO_y\}$-Einheiten über ihre Kanten und/oder Ecken miteinander verbinden. Nur in den seltensten Fällen erfolgt die Bindung über ganze Flächen. Der Grund dafür ist, dass in den erstgenannten Koordinationsmodi die Coulomb-Abstoßung zwischen den Addenda-Metallen aufgrund der größeren Metall-Metall-Abstände (im Vergleich zur der Verbindung über Flächen) deutlich verringert ist (Abb. 8).

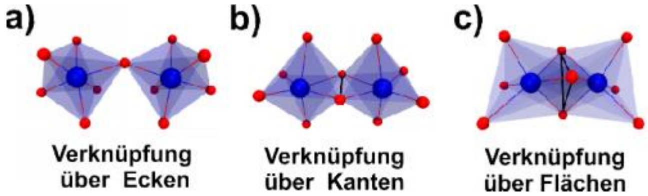

Abb. 8 Die Koordinationsarten von oktaedrischen $\{MO_6\}$-Einheiten. Die Koordination über die a) Ecken, b) Kanten und c) Flächen der Oktaeder sind dargestellt. Die Oktaeder sind deutlichkeitshalber transparent dargestellt. Die Kanten und Flächen der Koordinationsmodi b) und c) sind in schwarz hervorgehoben. Farbschema: dunkelblau, M; rot, Sauerstoff.

Isopolyanionen werden im Allgemeinen durch Ansäuern einer Lösung, die das Oxoanion des Addenda-Metalls enthält, synthetisiert. Dabei kommt es zu Kondensations-Additions-Reaktionen von Brønsted-Säuren und -Basen. Heteropolyanionen werden auf dieselbe Weise synthetisiert, jedoch in Anwesenheit eines Heteroatoms in Form eines Oxo- oder Hydroxoanions [42, 43]. Einfache Beispiele zur Synthese beider POM-Arten werden im Folgenden gezeigt, wobei Phosphor das Heteroatom im Synthesebeispiel des Heteropolyanions darstellt.

Isopolyanionen: $7\ WO_4^{2-} + 8\ H^+ \rightarrow [W_7O_{24}]^{6-} + 4\ H_2O$

Heteropolyanionen: $12\ MoO_4^{2-} + PO_4^{3-} + 24\ H^+ \rightarrow [PMo_{12}O_{40}]^{3-} + 12\ H_2O$

Es ist zu beachten, dass die oben gezeigten Reaktionsgleichungen stark vereinfacht sind, da der genaue Mechanismus der POM-Bildung, insbesondere die Triebkraft der Selbstassemblierung, nicht vollständig geklärt sind. Die Selbstassemblierung von POMs hängt von einer Reihe von Faktoren, wie z.B. dem pH-Wert der Reaktionslösung, der Konzentration der Konstituenten, der Temperatur, dem Reaktionsmedium, der Gegenionen, etc, ab [32]. Vor bereits mehr als 200 Jahren wurde die Stoffklasse der POMs postuliert und von einigen der berühmtesten Wissenschaftler wie W. Scheele (1742 - 1786), Jöns J. Berzelius (1779 - 1848) und Linus C. Pauling (1901 - 1994) untersucht. Im Jahre 1826 publizierte Berzelius die allererste Veröffentlichung, die die Synthese eines POMs beschreibt [44]. Dabei handelte es sich um das heute als $(NH_4)_3[PMo_{12}O_{40}]$ bekannte POM. Es dauerte jedoch weitere 85 Jahre bis James F. Keggin (1905 - 1993), ein Student des (bereits erwähnten) berühmten Nobelpreisträgers (1915 in Physik) und Gründers der molekularen Kristallographie William L. Bragg, die Struktur dieses ersten POMs kristallographisch bestimmte [45]. Keggin leistete auch in den darauffolgenden Jahren einen großen Beitrag zum Verständnis von POM-Strukturen und ihrer Selbstassemblierung, weshalb heutzutage ihm zu Ehren Strukturen der Form $[XM_{12}O_{40}]^{n-}$ (= $[XO_4\{(MO_{6/2})_3\}_4]^{n-}$) als Keggin-Strukturen bezeichnet werden (Abb. 9a).

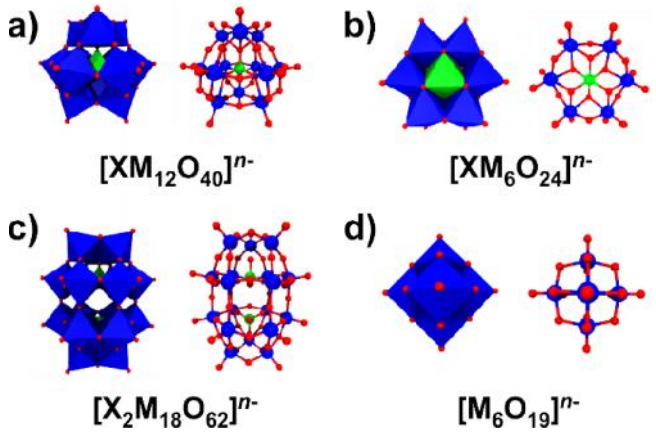

Abb. 9 Überblick über die bekanntesten POM-Archetypen. a) Keggin-, b) Anderson-Evans-, c) Wells-Dawson- und d) Lindqvist-Struktur. Die Strukturen sind sowohl in der Oktaeder- (links) als auch in ihrer Kugel-und-Stab-Darstellung (rechts) angezeigt. Farbschema: Dunkelblau, M; grün, X; rot, Sauerstoff.

Die Keggin-Struktur (α-isomer) besitzt eine tetraedrische Symmetrie (T_d) und besteht aus 12 oktaedrischen $\{MO_6\}$-Einheiten, die so angeordnet sind, dass sich jeweils drei $\{MO_6\}$-Einheiten über ihre Kanten verknüpfen und die größere $\{M_3O_{13}\}$-Einheit bilden. Vier dieser $\{M_3O_{13}\}$-Einheiten koordinieren über ihre Ecken miteinander und bilden die finale Keggin-Struktur (Abb. 10). Die Rotationswinkel der $\{M_3O_{13}\}$-Einheiten bestimmen die fünf Isomere der Keggin-Struktur, die als α-, β-, γ-, δ- und ε-Isomer bezeichnet werden (Abb. 11).

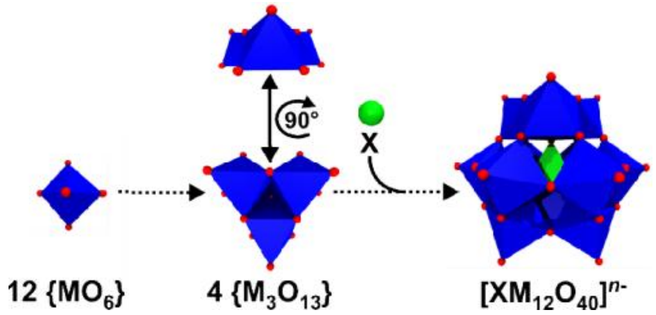

Abb. 10 Aufbau der Keggin-Struktur. In der Mitte der Abbildung ist die $\{M_3O_{13}\}$-Einheit von zwei verschiedenen Perspektiven gezeigt (unten und oben), um besser zu verdeutlichen, dass die ‚Kappe' (oberer Teil der Keggin-Struktur in der hier präsentierten Darstellung) und der ‚Gürtel' (unterer Teil der Keggin-Struktur in der hier präsentierten Darstellung) der Keggin-Struktur aus demselben Baustein (= $\{M_3O_{13}\}$-Einheit) aufgebaut sind. Farbschema: dunkelblau, M; grün, X; rot, Sauerstoff.

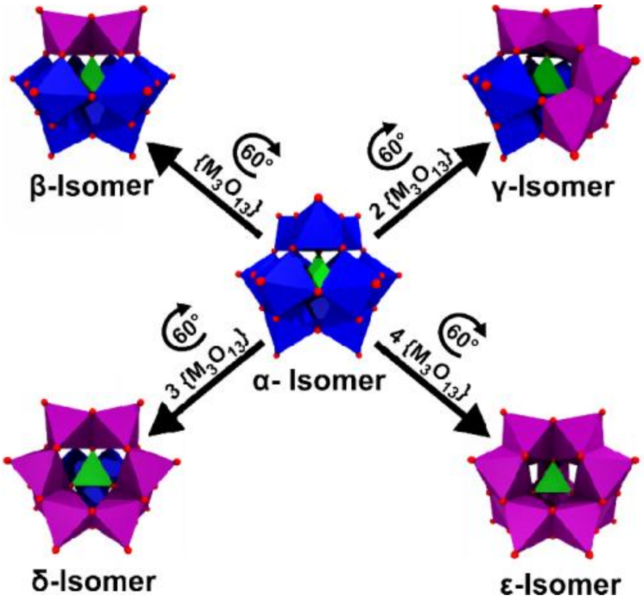

Abb. 11 Polyederdarstellung aller Keggin-Isomere. Die violett gefärbten $\{M_3O_{13}\}$-Einheiten stellen die Triaden dar, die das α-Isomer durch Drehung um 60° in ein anderes Isomer überführen. Farbschema: dunkelblau/violett, M; grün, X; rot, Sauerstoff.

Im Jahre 1937 postulierte John S. Anderson (1908 - 1990) die Struktur einiger 6-Heteropolysäuren wie die des Hexamolybdoperiodats $[I(Mo_6O_{24})]^{5-}$ [46, 47]. Ein Jahrzehnt später wurde Andersons Postulat durch die Kristallstrukturen der Hexamolybdotellurate $Z_6[TeMo_6O_{24}]$ ($Z = NH_4^+$ oder K^+), die durch Howard T. Evans Jr. (1920 - 2000) bestimmt wurden, strukturell bestätigt [48]. Deshalb werden heutzutage POMs der Form $[XM_6O_{24}]^{n-}$ (= $[XO_6(WO_{6/2})_6]^{n-}$) Anderson-Evans-Strukturen genannt (Abb. 9b). Die Anderson-Evans-Struktur, die von trigonaler Symmetrie (D_{3h}) ist, besteht aus einem zentralen, oktaedrisch angeordneten Heteroatom $\{XO_6\}$, welches von sechs über ihre Ecken miteinander verknüpften $\{MO_6\}$-Einheiten umschlossen ist, was zu einer planaren Anordnung der Struktur führt. Etwa zur gleichen Zeit (1939 - 1953) beschäftigten sich Alexander F. Wells (1912 - 1994) und Barrie Dawson (1925 - 1974) mit der Strukturaufklärung anderer POMs wie die der 18-Heteropolyoxowolframate. Während dieser Studien kam Dawson die Idee der Struktur des trigonalen Anions $[P_2W_{18}O_{62}]^{6-}$ [49]. 14 Jahre später wurde seine vorgeschlagene Struktur kristallographisch von seinem Kollegen Dawson bestätigt. Deshalb nennt man heute POMs, die die allgemeine Formel $[X_2M_{18}O_{62}]^{n-}$ (= $[(XO_4)_2(MO_{6/2})_{18}]^{n-}$) besitzen, Wells-Dawson-Strukturen (Abb. 9c) [50]. Das Wells-Dawson Polyanion (α-Isomer) mit seiner trigonalen Symmetrie (D_{3h}) entsteht durch die Zusammenführung von zwei $[XM_9O_{34}]^{n-}$-Bausteinen, die über ihre Ecken miteinander verbunden werden (Abb. 12).

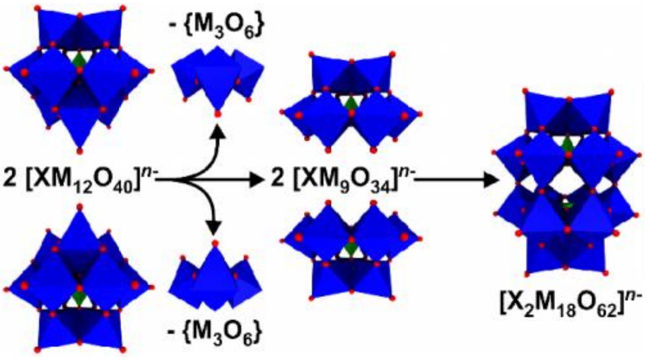

Abb. 12 Aufbau der Wells-Dawson-Struktur. Es ist zu beachten, dass das Addenda-Atom M der $\{M_3O_6\}$-Einheit nicht oktaedrisch koordiniert, da Sauerstoff-Atome zur Vervollständigung der oktaedrischen Anordnung fehlen. Die abgehende $\{M_3O_6\}$-Einheit ist in dieser Abbildung nur oktaedrisch aufgebaut, um grafisch deutlicher zu machen welcher Teil die Keggin-Struktur verlässt, um den trilakunären Baustein zu bilden. Für weitere Information, siehe Haupttext. Farbschema: dunkelblau, M; grün, X; rot, Sauerstoff.

Die $[XM_9O_{34}]^{n-}$-Einheit ist ein Keggin-Anion, dem eine $\{M_3O_6\}$-Triade fehlt. Solche Strukturen werden deshalb als trilakunäre Keggin-Strukturen bezeichnet. 1952 veröffentlichte Ingvar Lindqvist (1921 - 1991) am Beispiel des Natriumsalzes die erste Röntgenstrukturanalyse eines Parawolframats. Die von ihm vorgeschlagene Struktur

$[W_{12}O_{46}]^{20-}$ aus 12 $\{WO_6\}$-Oktaedern basierte allein auf der Bestimmung der Wolframlagen [51]. Im Laufe der folgenden Jahre wurde wiederholt erfolglos versucht, diese als korrekt angesehene Struktur mit anderen physikochemischen Analysenmethoden zu bestätigen. William N. Lipscomb Jr. (1919 - 2011) fiel als Erstem auf, dass keines der von ihm zum Vergleich herangezogenen POMs mehr als zwei terminale Sauerstoffatome per $\{WO_6\}$-Oktaeder enthielten, wie das Lindqvist für $[W_{12}O_{46}]^{20-}$ postuliert hatte. 1965 schlug der spätere Nobelpreisträger für Chemie (1976) unter Beibehalten der Wolframpositionen $[W_{12}O_{42}]^{12-}$ und für die protonierte Form $[H_2W_{12}O_{42}]^{10-}$ (= $[H_2(WO_{6/1.895})_6(WO_{6/1.565})_6]^{10-}$) vor [52]. Mittels ^{1}H-NMR-Breitlinienspektroskopie von Li-, Na- und K-Parawolframat konnte nachgewiesen werden, dass sich die beiden nichtaciden Protonen im zentralen tetraedrischen Hohlraum befinden [53]. Ein Jahr später wurde dieser Strukturvorschlag mittels Röntgenstrukturanalyse des Ammoniumparawolframat $(NH_4)_{10}[H_2W_{12}O_{42}]$ bestätigt [54]. Durch Neutronenbeugung am Ammoniumparawolframat konnten die beiden Protonen später exakt lokalisiert werden [55]. Neben der Postulierung der ersten (fehlerhaften) Metawolframatstruktur, löste Lindqvist kristallographisch die Strukturen weiterer POMs, unter anderem die des Hexaniobats $[Nb_6O_{19}]^{8-}$ (=$[(NbO_{6/1.89})_6]^{8-}$) [56]. Deshalb werden POMs der Form $[M_6O_{19}]^{n-}$ (=$[(MO_{6/1.89})_6]^{n-}$) Lindqvist-Strukturen genannt (Abb. 9d). Die Lindqvist-Struktur ist ein Isopolyanion (d.h. sie enthält kein Heteroatom), das aus sechs oktaedrisch angeordneten$\{MO_6\}$-Einheiten besteht (O_h-Symmetrie), wobei jede $\{MO_6\}$-Einheit über vier Kanten mit der benachbarten Einheit verbunden ist. Gegenwärtig sind Hunderte von POM-Verbindungen beschrieben, die fünf am häufigsten auftretenden Strukturen sind hier diskutiert worden.

Polyoxometallate in der Proteinkristallographie

Die Einführung der Klasse der POMs in den Bereich der Proteinkristallographie beruht einerseits auf wissenschaftlich rationalen Überlegungen und anderseits auf zufälligen Entdeckungen. Da POMs Schwermetall-Cluster (Mo, W, V, Nb und Ta) sind, sind sie ideale Werkzeuge für die Lösung des ‚Phasenproblems‘ mittels der SIR-/MIR-Methode. Einige POMs wie die Polyoxowolframate (POWs) eignen sich besonders gut für die Lösung des ‚Phasenproblems‘, da ihre Addenda-Atome nicht nur eine große Anzahl an Elektronen besitzen, sondern auch anomale Streuer sind. Da die L-I Absorptionskante des Wolframs bei 1.02 Å liegt, erzeugt das Übergangsmetall starke anomale Signale bei der Wellenlänge, die üblicherweise für das Röntgenbeugungsexperiment verwendet wird (~ 1 Å). Deshalb kann mit den POWs die häufiger angewendete SAD-/MAD-Methode zur Lösung des ‚Phasenproblems‘ verwendet werden. Aufgrund dieser Eigenschaften wurden POWs häufig als Werkzeuge zur Lösung des ‚Phasenproblems‘ verwendet [57]. Die Untersuchung aller Proteinkristallstrukturen, die in der PDB hinterlegt sind und POMs enthalten, und der dazugehörigen Literatur zeigte, dass die Anwesenheit der POMs häufig Zufall war, obwohl die Cluster einen großen Einfluss auf den Kristallisationsvorgang des Proteins hatten [40]. Die POMs wurden meistens nicht gezielt verwendet, da sie nicht als vorgefertigte Moleküle mit dem Ziel, die Kristallisation zu verbessern, zum Kristallisationsansatz gegeben wurden. Stattdessen bildeten sich die POMs während des Kristallisationsprozesses meistens spontan, da sich in den Kristallisationsbedingungen diverse Übergangsmetall-Oxoanionen (z.B. MO_4^{n-}; M = Mo^{6+} oder V^{5+}) befanden, die sich zu Polyanionen assemblierten. Es gibt mehrere rationale Gründe, Übergangsmetall-Oxoanionen in Proteinkristallisationsansätzen zu verwenden. Unter anderem fungieren sie als Inhibitoren (z.B. VO_4^{3-} für die bakterielle saure Phosphatase A [58]) oder als Substratanaloga (z.B. VO_3^- als Phosphat-Analog für das humane Zellzyklus regulierende Protein CksHs1 [59]) für verschiedene Enzyme, weswegen sie hauptsächlich als Additive verwendet werden. In dieser Hinsicht werden Vanadate aufgrund ihrer chemischen Ähnlichkeit zu Phosphat häufig als Phosphat-Mimetika oder Inhibitoren für Phosphat-abhängige oder Phosphat-umsetzende Proteine wie z.B. Phosphatasen verwendet. Des Weiteren können Vanadate fünffach-koordinierte Komplexe ausbilden, deren Koordinationsgeometrie eine gute Annäherung an den Übergangszustand von Phosphoryl-Transferreaktionen darstellt, was sie zusätzlich wertvoll macht als Phosphat-Mimetika oder Inhibitoren [60, 61]. Nichtsdestotrotz wurden POMs auch gezielt als Additive in der Proteinkristallisation eingesetzt, wobei sie sich positiv auf den Kristallisationsvorgang auswirkten. Tabelle 1 und 2 geben einen Überblick über die Proteinkristallstrukturen, die in der PDB hinterlegt sind und POMs enthalten. Die Tabellen fassen zusammen, welche POMs gezielt und mit welcher Ausrichtung eingesetzt wurden (Tabelle 1) und welche sich während des Kristallisationsverlaufs spontan ausbildeten (Tabelle 2). Damit ein POM kristallisationsfördernde Wirkungen ausüben kann, muss es in der Lage sein, mit den Biomakromolekülen zu wechselwirken, und deshalb werden im folgenden Abschnitt die POM-Protein-Wechselwirkungen kurz vorgestellt.

Tabelle 1 Überblick über die PDB-Einträge, die POMs enthalten, die gezielt für die Kristallisation verwendet wurden (Stand: Mai 2018).

PDB Eintrag	Protein (Organismus)	POM	Ziel des POM-Einsatzes	Auswirkungen auf die Kristallisation	Ref
1DV4	Kleine ribosomale Untereinheit (*T. thermophilus*)	$[P_2W_{18}O_{62}]^{6-}$	Phasing	Stabiliserung und Rigidifizierung der Struktur	[62]
1FKA	Kleine ribosomale Untereinheit (*T. thermophilus*)	$[P_2W_{18}O_{62}]^{6-}$	Phasing	Stabiliserung und Rigidifizierung der Struktur	[63]
1I94[a]	Kleine ribosomale Untereinheit (*T. thermophilus*)	$[P_2W_{18}O_{62}]^{6-}$	Phasing	Stabiliserung und Rigidifizierung der Struktur	[64]
1N7D	LDL Rezeptor (human)	$[PW_{12}O_{40}]^{3-}$	Phasing	Stabilisierung der Domänen-Organisation und Verbesserung der Kristallqualität	[65]
2G8H	DNA Reperatur Protein Rad51 (*M. voltae*)	$[H_2W_{12}O_{40}]^{6-}$	Inhibitor	Stabilisierung der inaktiven Form	[66]
3ZX0	NTPDase1 (*R. norvegicus*)	$[Mo_7O_{24}]^{6-}$	Inhibitor	Stabilisierung der Struktur	[67]
3XZ2	NTPDase1 (*R. norvegicus*)	$[V_{10}O_{28}]^{6-}$	Inhibitor	Stabilisierung der Struktur	[67]
4BVO	NTPDase 1 (*L. pneumophila*)	$[H_2W_{12}O_{40}]^{6-}$	Inhibitor	Vermittlung von Kristallkontakten und Stabilisierung der Struktur über Rigidifizierung	[68]
4BVP	NTPDase 1 (*L. pneumophila*)	$[Mo_8O_{28}]^{8-}$	Inhibitor	Stabilisierung der Struktur über Rigidifizierung	[68]
4BVP	NTPDase 1 (*L. pneumophila*)	$[Mo_7O_{24}]^{6-}$	Inhibitor	Stabilisierung der Struktur über Rigidifizierung	[68]
4PE5	NMDA Rezeptor-Ionenkanal (*R. norvegicus*)	$[H_2W_{12}O_{40}]^{6-}$	Phasing	Verbesserung der Kristallqualität	[69]
4OUA	Tyrosinase (*A. bisporus*)	$[TeW_6O_{24}]^{6-}$	Additiv	Vermittlung von Kristallkontakten	[70]
4PHI	Lysozym (*G. gallus*)	$[TeW_6O_{24}]^{6-}$	Additiv	Vermittlung von Kristallkontakten (neue Kristallform)	[71]
4Z12	Auronsynthase (*C. grandiflora*)	$[TeW_6O_{24}]^{6-}$	Additiv	Vermittlung von Kristallkontakten und Verbesserung der Kristallqualität	[72]
4XYY	Lysozym (*G. gallus*)	$[Zr(PW_{11}O_{39})]^{3-}$	Künstliche Protease[b]	Keine	[73]
5FHW	Lysozym (*G. gallus*)	$[Hf(P_2W_{17}O_{61})]^{6-}$	Künstliche Protease[b]	Keine	- [c]
5WP6	TRPM4 Kanal (human)	$[V_{10}O_{28}]^{6-}$	Kanal-Modulator	Struktur wurde mittels Elektronenmikroskopie gelöst	[74]
5SUQ	Sub2 ATPase-THO Komplex (*S. cerevisiae*)	$[PW_{12}O_{40}]^{3-}$	Phasing	Keine Angabe in der Publikation	[75]
6G3S	HSP70 Nukleotid-Bindedomäne (human)	$[TeW_6O_{24}]^{6-}$	Additiv	Induktion einer ungewöhnlichen Kristallpackung	[76]

[a] Die PDB Einträge 1I95, 1I96 und 1I97 enthalten dasselbe POM (mit gleicher Auswirkung auf die Kristallisation), weshalb diese Einträge in dieser Tabelle ausgelassen wurden. [b] Das POM ist proteolytisch aktiv (schneidet das Protein) und die angegebene Struktur wurde zur Verifizierung seiner Bindestelle am Protein verwendet. [c] Zu diesem Eintrag gibt es bislang noch keine Publikation.

Tabelle 2 Überblick über die PDB-Einträge, die POMs enthalten, die sich spontan während der Kristallisation ausbildeten (Stand: Mai 2018).

PDB Eintrag	Protein (Organismus)	POM	Ursprung des POMs	Auswirkungen auf die Kristallisation	Ref.
1DKT	Zellzyklus regulierendes Protein CksHs1 (human)	$[V_7O_{19}]^{3-*}$	Selbstassemblierung in Anwesenheit von VO_3^- (Phosphat-Analog)	Stabilisierung des Proteindimeres	[59]
1E59	Cofactor-abhängige Phosphoglyceratmutase (*E. coli*)	$[V_4O_{13}]^{6-}$	Selbstassemblierung in Anwesenheit von VO_4^{3-} (Inhibitor)	Stabilisierung der inaktiven Form	[77]
1L7V	ABC Transporter (*E. coli*)	$[V_4O_{12}]^{4-}$	Selbstassemblierung in Anwesenheit von VO_4^{3-} (Inhibitor)	POM wurde für das Phasing verwendet	[78]
1UZI	C3 Exoenzym (*C. botulinum*)	$[V_4O_{12}]^{4-}$	Selbstassemblierung in Anwesenheit von VO_4^{3-}	Vermittlung von Kristallkontakten	[79]
1RXS	Uridin-Phosphorylase (*E. coli*)	$[V_7O_{19}]^{3-*}$	Selbstassemblierung in Anwesenheit von VO_3^-	Keine	[80]
2D1G	Saure Phosphatase A (*F. tularensis*)	$[V_{10}O_{28}]^{6-}$	Selbstassemblierung in Anwesenheit von VO_4^{3-} (Inhibitor)	Stabilisierung der Struktur über Rigidifizierung	[58]
2G8H	RNase H (*B. halodurans*)	$[V_6O_{19}]^{8-*}$	Selbstassemblierung in Anwesenheit von VO_3^- (Substrat-Mimetikum)	Stabilsierung einer Übergangsstruktur	[81]
2HHL	CTD kleines Phosphatase-ähnliches Protein (human)	$[PW_{12}O_{40}]^{3-}$	Nicht beschrieben in der Publikation	Vermittlung von Kristallkontakten	[82]
1P0Z	Sensorkinase CitA (*K. pneumonia*)	$[Mo_7O_{24}]^{6-}$	Selbstassemblierung in Anwesenheit von MoO_4^{2-} (Inhibitor)	Vermittlung von Kristallkontakten	[83]
2OGX	Molybdän-Speicherprotein (*A. vinelandii*)	$[W_3O_{13}]^{8-*}$	Selbstassemblierung in Anwesenheit von WO_4^{2-}	Keine [a]	[84]
4F6T	Molybdän-Speicherprotein (*A. vinelandii*)	$[Mo_8O_{26}H_n]^{n-5*}$	Protein-induzierte Assemblierung in Anwesenheit von MoO_4^{2-}	Keine [a]	[85]
4F6T	Molybdän-Speicherprotein (*A. vinelandii*)	$[Mo_8O_{28}]^{8-*}$	Protein-induzierte Assemblierung in Anwesenheit von MoO_4^{2-}	Keine [a]	[85]
4F6T	Molybdän-Speicherprotein (*A. vinelandii*)	$[Mo_6O_{27}H_n]^{n-18*}$	Protein-induzierte Assemblierung in Anwesenheit von MoO_4^{2-}	Keine [a]	[85]
4BRH	NTPDase 1 (*L. pneumophila*)	$[V_{10}O_{28}]^{6-}$	Selbstassemblierung in Anwesenheit von VO_4^{3-} (Phosphat-Mimetikum)	Keine [a]	[86]
4NDO[b]	Molybdän-Speicherprotein (*A. vinelandii*)	$[Mo_3O_{13}]^{8-*}$	Protein-induzierte Assemblierung in Anwesenheit von MoO_4^{2-}	Keine [a]	[87]
4NDO[b]	Molybdän-Speicherprotein (*A. vinelandii*)	$[Mo_8O_{28}]^{8-}$	Protein-induzierte Assemblierung in Anwesenheit von MoO_4^{2-}	Keine [a]	[87]
5O5W	Molybdän-Speicherprotein (*A. vinelandii*)	$[Mo_8O_{28}]^{8-}$	Protein-induzierte Assemblierung in Anwesenheit von MoO_4^{2-}	Keine [a]	[88]
3GQI	Rezeptor-Tyrosinkinase (human)	$[V_{10}O_{28}]^{6-}$	Selbstassemblierung in Anwesenheit von VO_4^{3-} (Inhibitor)	Strukturstabilisierung	[89]
4B1A	Lysozym (*G. gallus*)	$[PMo_{12}O_{40}]^{3-}$	Selbstassemblierung nach dem Zerfall eines Mo- Komplexes	Strukturstabilisierung	[90]
5XLS	Uracil:Protein-Symporter (*E. coli*)	$[PW_{12}O_{40}]^{3-}$	Selbstassemblierung in Anwesenheit von $(NH_4)_2WS_4$	POM wurde für das Phasing verwendet	[91]

[a] Das Molybdän-Speicherprotein besitzt eine Quartärstruktur, die an ein Fass erinnert und in dessen Innerem das Protein sowohl Mo- als auch W-Atome in Form von POMs speichert. Deshalb wird die Assemblierung der POMs in diesem Fall aus physiologischen Gründen durch das POM induziert. [b] Die PDB Einträge 4NDP, 4NDQ und 4NDR enthalten dasselbe POM (mit gleicher Auswirkungen auf die Kristallisation), weshalb sie in dieser Tabelle ausgelassen wurden. * Dieses POM wurde in dieser Zusammensetzung noch nie aus einer Lösung isoliert.

POM-Protein-Wechselwirkungen

Aufgrund ihrer negativen Ladung sind POMs geradezu prädestiniert, um mit positiv geladenen Proteinregionen über ihre Ladungen bzw. allgemein über elektrostatische Wechselwirkungen zu interagieren (Abb. 13a). Der Nachweis, dass elektrostatische Wechselwirkungen die Hauptantriebskraft für POM-Protein-Wechselwirkungen darstellen, wurde durch einige Studien, die die Wechselwirkung einiger POMs mit humanem Serumalbumin (HSA) untersucht haben [92, 93, 94, 95, 96], und natürlich durch die Röntgenstrukturanalyse mehrerer Protein-POM-Komplexe erbracht [40, 97]. Die Kristallstrukturen zeigten, dass hauptsächlich positive geladene Aminosäuren (Lysin, Arginin und Histidin) an den Wechselwirkungen mit den POMs beteiligt sind. Des Weiteren können POMs auch mit Protonen-Donatoren (Serin, Threonin, Cystein, Tyrosin, Asparagin und Glutamin) mittels Wasserstoffbrückenbindungen wechselwirken (Abb. 13b). Basierend auf dieser Art von Wechselwirkungen sind POMs in der Lage, mit polaren Lösungsmittelmolekülen (z.B. Wasser) und/oder mono- und multivalenten Kationen (z.B. Mg^{2+}) zu interagieren. Auf diese Weise können POMs über Lösungsmittel- oder Kationen-vermittelte Interaktionen ‚indirekt' mit Proteinen wechselwirken. Das bedeutet, dass ein Lösungsmittelmolekül oder Kation das POM mit dem Protein in Verbindung bringt, indem es gleichzeitig mit beiden Molekülen über Wasserstoffbrückenbindungen bzw. elektrostatische Wechselwirkungen interagiert (Abb. 13c und 13d). Die Kationen-vermittelten Wechselwirkungen ermöglichen dem POM sogar Wechselwirkungen mit negativ geladenen Aminosäuren (Glutamin- und Asparaginsäure) einzugehen, die normalerweise durch elektrostatische Abstoßung verhindert werden (Abb. 13d) [84, 85]. Es wurden jedoch auch POM-Protein-Wechselwirkungen an hydrophoben Bereichen von Proteinen beobachtet. Beispielsweise interagieren verschiedene Polyoxomolybdate (POMos) überwiegend mit hydrophoben Aminosäuren (z.B. Valin, Prolin und Glycin) des Molybdän-Speicherproteins *via* van der Waals-Wechselwirkungen (Abb. 13e) [87]. Dies kann durch die hohe Polarisierbarkeit der POMs erklärt werden, die anscheinend die dauerhaften Dipol-induzierten Dipol-Wechselwirkungen mit mehr oder weniger hydrophoben Proteinbereichen verstärkt. Die Fähigkeit von POMs, hydrophobe Wechselwirkungen einzugehen, wurde durch die Erkenntnis, dass POMs eine hohe Affinität gegenüber neutralen und hydrophoben Oberflächen (Wechselwirkungen mit nicht-ionischen Tensiden und Membranlipiden) besitzen, weiter bestätigt [98, 99, 100, 101]. POMs können abhängig von ihrer Ladungsdichte, Umgebung (z.B. Gegenionen) und Reaktionspartner zwischen elektrostatischen und hydrophob-ähnlichen Wechselwirkungen wechseln. Beispielsweise neigen POMs mit einer niedrigeren Ladungsdichte stärker dazu, hydrophobe Wechselwirkungen einzugehen, was dazu führt, dass diese POMs eine höhere Affinität zu Membranmimetika haben. Einige POMs sind sogar in der Lage, mit Membranmimetika, die negative geladene Kopfgruppen haben, zu interagieren [101]. In diesen Fällen wird die negative Ladung der Kopfgruppen im Wesentlichen durch die Gegenkationen der POMs neutralisiert, wodurch dem POM die Adsorption an die Alkylregion der Membran ermöglicht wird. Interessanterweise wurden auch kovalente Bindungen zwischen POMs und den Aminosäuren von Proteinen beobachtet (Abb. 13f) [72, 84, 85]. Die Kristallstruktur des Molybdän-Speicherproteins zeigte, dass einige Oktamolybdate $[Mo_8O_{26}]^{4-}$ kovalent an das $N_{\varepsilon 2}$ Stickstoff-Atom eines Histidins und an das $O_{\varepsilon 1}$ Sauerstoff-Atom einer Glutaminsäure gebunden waren. Die Bildung dieser POMs wurde durch das Protein induziert, da die Hauptfunktion des Proteins die Speicherung von Mo- und W-Atomen in Form von Polyanionen ist. Aus diesem Grund könnten die beobachteten kovalenten Bindungen durch den Protein-getriebenen Assemblierungsprozess entstanden sein. Eine ähnliche Beobachtung wurde auch bei der Strukturlösung der bakteriellen Nukleotidtriphosphatdiphosphohydrolase 1 (NTPDase1) festgestellt, wo ebenfalls ein Oktamolybdat vom Hydroxyl-Sauerstoff eines Serins kovalent gebunden wurde [68]. Das POMo wurde nicht als intaktes Molekül zum experimentellen Ansatz gegeben, sondern bildete sich spontan aus den in der Kristallisationslösung vorhandenen MoO_4^{2-}-Ionen. Die einzige Studie, bei der ein vorgefertigtes POM als Kristallisationsadditiv verwendet wurde und es zu einer kovalenten Bindung kam, war während der Kristallisation der pflanzlichen Auronsynthase von *Coreopsis grandiflora* (*Cg*AUS1) mit dem Anderson-Evans-Anion $[TeW_6O_{24}]^{6-}$ [72, 102]. Die kovalente Bindung wurde hierbei zwischen dem Hexawolframatotellurat und einer Glutaminsäure geschlossen, was die Fähigkeit von POMs, kovalente Bindungen mit Proteinen auszubilden, weiter bekräftigt. Der Mechanismus für die Entstehung dieser kovalenten Bindung ist nicht bekannt.

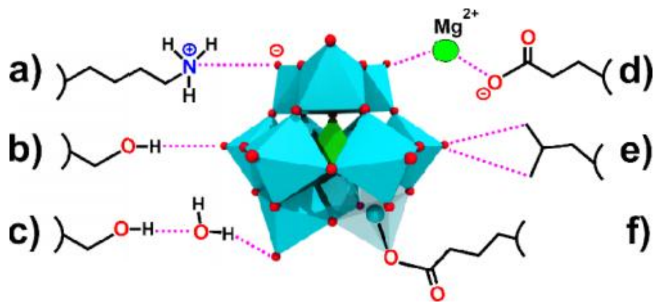

Abb. 13 Schematische Darstellung von POM-Protein-Wechselwirkungen. a) Elektrostatische Wechselwirkung zwischen den Ladungen des negativ geladenen POMs und des positiv geladenen Lysins. b) Wasserstoffbrückenbindung zwischen dem POM und einem Serin. c) Lösungsmittel (Wasser)-vermittelte Wasserstoffbrückenbindung zwischen dem POM und einem Serin. d) Kation (Mg^{2+})-vermittelte elektrostatische Wechselwirkung, die zwei negativ geladene Moleküle (POM und Aspartat) miteinander verbindet. e) van der Waals- oder hydrophobe Wechselwirkung zwischen dem POM und einem Leucin. f) Kovalente Bindung zwischen dem POM und einer Glutaminsäure. Die {MO_6}-Einheit, die die kovalente Bindung eingeht, ist transparent, wobei das Addenda-Atom M als Kugel angezeigt wird, um die kovalente Bindung genauer darzustellen. Farbschema: zyan, M; grüner Oktaeder, X; grüne Kugel, Magnesium; rot, Sauerstoff.

Polyoxometallate als Werkzeug zum Lösen des ‚Phasenproblems'

Wie bereits erwähnt wurden POMs hauptsächlich zum Lösen des ‚Phasenproblems' verwendet. POWs sind hierbei eine besonders gute Wahl, da ihre Wolfram-Atome elektronenreiche, anomale Streuer sind, weswegen sie wie geschaffen sind für das Phasing mittels der SAD-/MAD-Methode. Eine bestimmte Anzahl an Schweratomen bzw. anomalen Streuern pro Aminosäure muss im Proteinkristall vorhanden sein, damit ausreichend starke isomorphe bzw. anomale Signale entstehen, die sich vom Hintergrund (Signalrauschen) deutlich hervorheben. Dies ist besonders wichtig für die Lösung des ‚Phasenproblems' großer Proteine oder makromolekularer Komplexe, da mit steigender Größe des Proteins (Anzahl der Aminosäuren) eine größere Menge an schweren Atomen bzw. anomalen Streuern nötig ist [103]. In dieser Hinsicht haben POMs als große und dichte Metallkomplexe einen entscheidenden Vorteil gegenüber den für gewöhnlich verwendeten einzelnen Schweratomen (z.B. Hg^{2+}, Au^{3+} oder $Pt^{2+/4+}$), da eine geringere Anzahl an POMs in die Proteinstruktur eingebracht werden muss im Vergleich zu der Anzahl der einzelnen Schweratome, die nötig sind, um ein messbares Signal zu erzeugen [104]. Die Bindung eines einzelnen POMs führt bereits mehrere Schweratome bzw. anomale Streuer in die Struktur ein. Um die experimentellen Phasen bei hoher Auflösung direkt zu berechnen, müssen die Positionen der einzelnen Schweratome bzw. anomalen Streuer innerhalb des POMs bestimmt werden und das POM muss korrekt ausgerichtet sein. Dies kann ziemlich schwierig werden, wenn die Qualität der mit POM derivatisierten Kristalle gering ist. Metall-Cluster wie POMs liefern jedoch auch brauchbare Phasing-Signale bei niedrigen Auflösungen, wo die Positionen der einzelnen Metall-Atome nicht bestimmt werden kann. In diesem Fall streuen die einzelnen Metall-Atome des POMs in Phase und agieren dabei als eine Art ‚Superatom' [57]. Der Gesamtbeitrag zum Streusignal dieses ‚Superatoms' setzt sich aus der Summe aller (anomalen) Elektronen des POMs zusammen, was zu einem deutlich höheren Phasing-Signal führt im Vergleich zu dem Szenario mit zufällig verteilten, einzelnen schweren Atomen bzw. anomalen Streuern [57]. Ein POM, das aus N Metall-Atomen und Z Elektronen besteht, liefert bei niedriger Auflösung einen Beitrag zum Streusignal von $(NZ)^2$, da die Intensität der gestreuten Röntgenstrahlen proportional zum Quadrat der Anzahl der streuenden Elektronen ist. Im Gegensatz dazu beträgt der Beitrag der einzelnen Schweratome bzw. anomalen Streuer zum Streusignal $N(Z)^2$, welcher im Vergleich zum Streubeitrag des ‚Superatoms' (POM) um den Faktor N geringer ist [57]. Auf diese Weise erhält man durch POM-derivatisierte Kristalle starke isomorphe bzw. anomale Signale, wobei der Massenschwerpunkt des POMs für die Lösung des ‚Phasenproblems' ganz genau bestimmt werden kann. Ein weiterer Aspekt, weshalb einige POMs besonders gut geeignet sind für das Phasing im Vergleich zu den häufig verwendeten Phasing-Werkzeugen (z.B. einzelne Metallionen, kleine Metallkomplexe, Halide) ist, dass sie in wässrigen Lösungen stabil sind. In Kristallisationslösungen befinden sich häufig verschiedene anorganische und/oder organische Moleküle wie z.B. Additive, Pufferkomponenten, Stabilisatoren und/oder Fällungsmittel, die mit der Phasing-Verbindung chemisch interferieren und somit ihre Funktionalität hemmen könnten. Beispielsweise beeinträchtigen einige Puffer wie z.B. der TRIS- (Tris(hydroxymethyl)aminomethan), Phosphat- oder Citratpuffer die Bindung einiger Schwermetalle an das Protein, indem sie mit ihnen teilweise unlösliche Komplexe ausbilden (z.B. Lanthanide und Phosphat) [14]. Im Gegensatz dazu sind einige POMs, insbesondere das Anderson-Evans-Anion stabil, wobei sie gegenüber den häufig verwendeten Bestandteilen einer Kristallisationslösung weitestgehend inert sind [41].

Verschiedene POWs wie das Keggin-Anion $[PW_{12}O_{40}]^{3-}$ und die Wells-Dawson-Struktur $[P_2W_{18}O_{62}]^{6-}$ wurden bereits frühzeitig für das Lösen des ‚Phasenproblems‘ großer Proteine und molekularer Komplexe verwendet, wie z.B. bei der Strukturlösung der bakteriellen Riboflavinsynthase [105], der bakteriellen Fumarase C [106], des 20S-Hauptkomplexes des Proteasoms eines Archaeons [107], der bakteriellen 50S-Untereinheit des Ribosoms [108], der RNA-Polymerase II aus Hefe [109] und des humanen LDL-Rezeptors (low-density lipoprotein receptor) [110]. Deshalb bietet die Firma Jena Bioscience (www.jenabioscience.com) sogenannte ‘Phasing-Kits’ an, die das Keggin-Anion $[PW_{12}O_{40}]^{3-}$, das Metawolframat $[H_2W_{12}O_{40}]^{6-}$ und das Parawolframat $[H_2W_{12}O_{42}]^{10-}$ als Derivatisierungs-anionen beinhalten.

POMs als Kristallisationsadditive

Neben ihrer erfolgreichen Verwendung als Werkzeug zum Lösen des ‘Phasenproblems’ wirkten sich POMs auch positiv auf den Kristallisationsprozess einiger Proteine aus [40]. Die kristallisationsfördernde Wirkung der POMs beruht hauptsächlich auf ihrer Fähigkeit, Kristallkontakte zu schaffen und diese zu stabilisieren. Aufgrund der überwiegend elektrostatischen Wechselwirkungen (inklusive Wasserstoffbrückenbindungen) mit positiv geladenen und/oder polaren Bereichen der Proteinoberfläche sind POMs in der Lage, Proteinmoleküle miteinander zu ‚verbrücken‘, was zu neuen Protein-Protein-Kontakten führt. Insbesondere das ‚Crosslinking‘ positiv geladener Proteinoberflächen über ionische Wechselwirkungen mit dem POM ist von großem Vorteil, da sich diese Oberflächen ansonsten elektrostatisch abstoßen würden. Durch das ‚Zusammenkleben‘ dieser gleich geladenen Proteinbereiche mittels des POMs wird die Anzahl der möglichen Kristallkontakte erhöht und somit auch die Wahrscheinlichkeit einer Kristallisation des Proteins. Außerdem sind POMs in der Lage, sowohl biologische als auch kristallographische oligomere Zustände (z.B. ein Dimer), die sich unabhängig vom POM bilden, zu stabilisieren. Die Fähigkeit der POMs, Proteinmoleküle auf eine die Kristallisation fördernde Art und Weise miteinander zu ‚verbrücken‘, hängt von einigen Faktoren wie z.B. der Ladung, Ladungsdichte, Größe, Form und Symmetrie ab. Die Ladung bzw. Ladungsdichte und die Größe bzw. Form des POMs bestimmt hauptsächlich seine Bindungsaffinität zu Proteinen. Da, unter anderem, die Ladungsdichte für die Hydrophilie des POMs ausschlaggebend ist, muss dieser Parameter sinnvoll gewählt werden, um ausreichende elektrostatische Wechselwirkungen mit dem Protein zu gewährleisten. POMs, die eine zu hohe Ladungsdichte besitzen, sind generell stärker hydratisiert und neigen somit eher dazu, in Lösung zu bleiben. Der Grund dafür ist, dass die Solvatisierungs- bzw. Desolvatisierungsenergie, die nötig ist, um die Hydrathülle des POMs für eine Wechselwirkung mit dem Protein zu entfernen, zu groß ist. POMs, die wiederum eine zu geringe Ladungsdichte besitzen, gehen nur schwache und promiskuitive elektrostatische Wechselwirkungen ein [94, 111, 112]. Die Größe und Form des POMs können eine entscheidende Rolle in dessen Bindefähigkeit und ‚Verbrückungspotential‘ spielen. Größere POMs bieten potenziell mehr Raum für Interaktionen (bzw. mehr Bindestellen), der es ihnen ermöglicht, mit einer größeren Anzahl an Aminosäuren und Proteinmolekülen zu wechselwirken. Kleine POMs hingegen sind diesbezüglich eher limitiert. Die allgemein beachtliche Größe der POMs erlaubt es ihnen, als eine Art Distanzhalter zwischen den ‚verbrückten‘ Proteinmolekülen zu fungieren (Abb. 14). Dieser Abstand kann sehr wichtig sein, da mögliche weitreichende Abstoßungskräfte oder sterische Beeinträchtigungen zwischen den ‚verbrückten‘ Proteinmolekülen reduziert werden. Dadurch wird den Proteinmolekülen die räumliche Annäherung zueinander erleichtert und gleichzeitig stabilisiert das POM die (short-range) Anziehung zwischen sich ansonsten elektrostatisch abstoßenden Oberflächen, was von besonderer Bedeutung für den Prozess der Keimbildung ist. Deshalb ist eine Ausgewogenheit zwischen der Größe (bzw. Form) und der Ladung des POMs entscheidend für ideale POM-Protein-Wechselwirkungen, die sich nicht nur auf elektrostatische Wechselwirkungen und Wasserstoffbrückenbindungen, sondern auch auf hydrophobe Interaktionen bezieht.

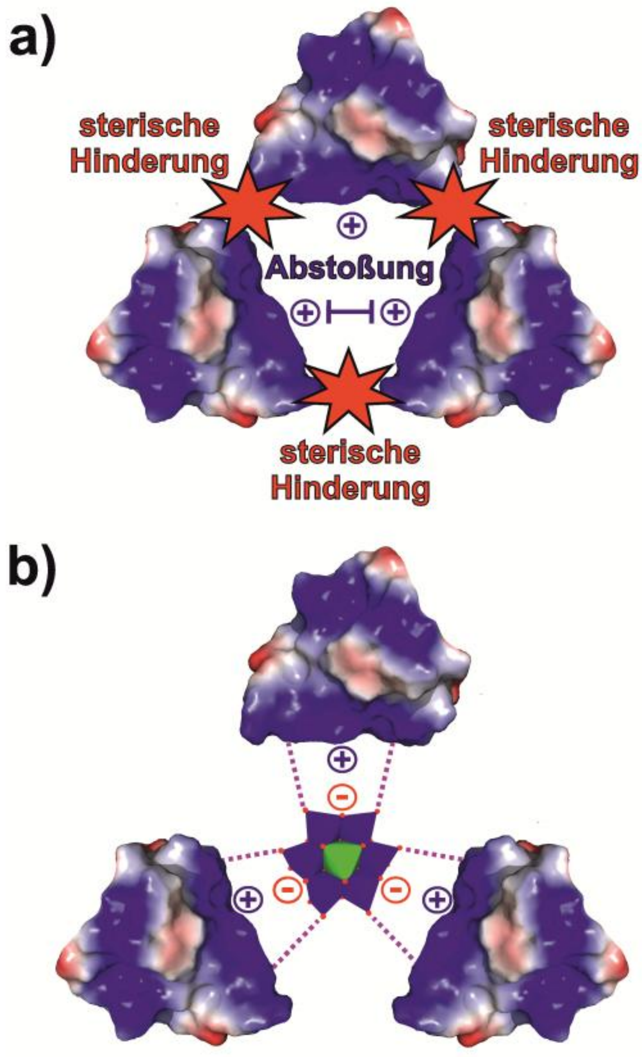

Abb. 14 Schematische Darstellung wie der POM als ‚Distanzhalter‘ zwischen ‘verbrückten’ Proteinmolekülen fungieren kann. Als Anschauungsbeispiel dient das Anderson-Evans POM. a) Eine Situation ist dargestellt, in der sich drei positiv geladene Proteinregionen (dargestellt als elektrostatische Coulomb-Oberflächen, blau = positives Potential, rot = negatives Potential und weiß = neutrales Potential) annähern, jedoch wird die Ausbildung eines (Kristall-)Kontakts sowohl durch sterische Kollisionen (rote Sterne) als auch elektrostatische Abstoßung verhindert. b) Anderson-Evans POM-vermittelte Ausbildung eines Kristallkontakts. In der Anwesenheit des POMs können die gleichförmig geladenen Moleküle elektrostatisch miteinander ‚verbrückt‘ werden, während sie trotzdem noch ausreichend voneinander entfernt sind, um sterische Kollisionen untereinander zu verhindern. Farbschema: dunkelblau, M; grün, X; rot, Sauerstoff.

Die Symmetrie kann ebenfalls eine wichtige Rolle bei der POM-vermittelten Proteinkristallisation spielen, da sie die Bindestelle des POMs innerhalb des Kristallgitters selektiv bestimmen kann [105]. Falls die innere Symmetrie eines POMs mit der kristallographischen und/oder nicht-kristallographischen Symmetrie eines Proteinkristalls korreliert, dann besteht eine gewisse Wahrscheinlichkeit, das POM auf dem entsprechenden (gemeinsamen) Symmetrieelement zu finden. Die Kristallisation der Riboflavinsynthase zeigte, dass beispielsweise der trigonale Cluster $[W_3O_2(O_2CCH_3)_6]^{2+}$ (D_3 Symmetrie) an die dreifache Rotationsachse des Proteins gebunden hatte, während das pentagonale Heteropolywolframat $[NaP_5W_{30}O_{110}]^{14-}$ (auch bekannt als Preyssler-Anion, D_5 Symmetrie) sich auf der internen fünffachen Rotationsachse des Proteins befand [113]. Die Symmetrie kann auch den ‚Verbrückungsgrad‘ eines POMs beeinflussen, da ein Polyanion, welches sich beispielsweise auf einer X-fachen Rotationsachse befindet, mit X Symmetrie-verwandten Proteinmolekülen wechselwirken kann (Abb. 15). Es ist jedoch zu beachten, dass eine Symmetrieübereinstimmung zwischen dem POM und dem Kristallgitter (oder der inneren Symmetrie der biomolekularen Einheit) keine Voraussetzung für die POM-Bindung oder dessen Fähigkeit, die Proteinkristallisation zu fördern, ist, da in den meisten Fällen die POMs abseits der Symmetrieelemente binden. Zwei weitere, größere Probleme in der Proteinkristallisation sind die Flexibilität und das dynamische Verhalten von Proteinen. Proteine, die strukturell hochflexibel und beweglich sind, zeigen eine deutlich geringere Tendenz zur Kristallisation als weniger bewegliche Proteine, da flexible Bereiche sich nicht einfach in ein periodisches Gitter (Kristall) einordnen lassen. Die flexible Natur eines Proteins spiegelt sich auch häufig in der Kristallstruktur wider,

die teilweise lückenhaft ist, da bewegliche Domänen, insbesondere unstrukturierte Loops (schleifenförmige Sekundärstrukturelemente innerhalb von Proteinstrukturen), aufgrund ihrer erhöhten Bewegung und Schwingungen nicht modelliert werden können. Die Zeitspanne dieser Schwingungen ist deutlich kürzer als die Dauer des Röntgenbeugungsexperimentes (bzw. als die Dauer der Röntgeneinstrahlung), weshalb man eine Schar an verschiedenen Positionen, die sich über ein größeres Volumen verteilt, detektiert. Damit ist die Bestimmung der genauen bzw. ungefähren Position solcher flexiblen Proteinfragmente sehr schwierig oder gar unmöglich (deshalb lässt man diese Teile eines Proteins beim Modellieren aus, was zu Lücken im Proteinmodell führt). POMs sind aber in der Lage solche flexiblen Proteinregionen zu fixieren und zu stabilisieren, indem sie an diese Bereiche binden [40]. Auf diese Weise werden die konformationelle Stabilität des Proteins und die Wahrscheinlichkeit einer Kristallisation erhöht. Da die strukturelle Mobilität eines Proteins meistens mit dessen Funktion zusammenhängt, können POMs auch als Inhibitoren bestimmter Enzyme wirken, indem sie die für die katalytische Funktion essentielle Domänenbewegungen stören oder blocken. Dank dieser Eigenschaft konnten bestimmte (katalytische) Übergangzustände von Enzymen stabilisiert und kristallisiert werden [40]. Beispielsweise sorgte die Bindung von $[H_2W_{12}O_{40}]^{6-}$ an NTPDase1 nicht nur für die Hemmung der Aktivität des Enzyms, sondern auch für die Stabilisierung einer halb-offenen Konformation, die kristallographisch bestimmt werden konnte [68]. Die dabei entstandene Kristallstruktur lieferte wertvolle Hinweise auf die Domänenbewegungen des Enzyms während der katalytischen Reaktion. Zusammenfassend wird festgestellt, dass sich POMs positiv auf die Kristallisation einiger Proteine ausgewirkt haben, indem sie vor allem Kristallkontakte durch das ‚Verbrücken' von Proteinmolekülen geschaffen haben und die Beweglichkeit flexibler Proteinbereiche reduziert haben.

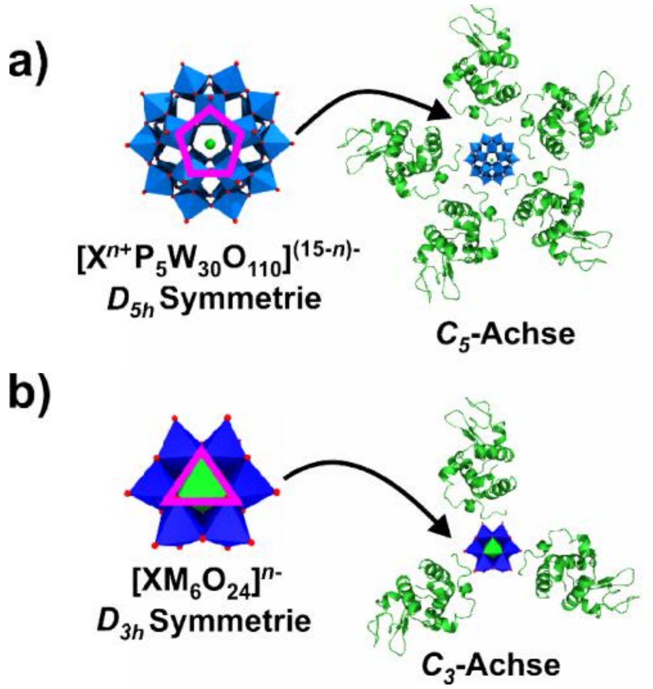

Abb. 15 Schematische Darstellung des Einflusses der Symmetrie auf das Bindeverhalten von POMs. a) Die Symmetrie bestimmt die Position des $[X^{n+}P_5W_{30}O_{110}]^{(15-n)-}$ Anions auf einer fünffachen Rotationsachse, da das Anion eine innere D_{5h} Symmetrie besitzt. Da sich das POM auf einer fünffachen Drehachse befindet, steht es mit fünf Proteinmolekülen in Wechselwirkung. Die C_5 Symmetrie des Preyssler-Anions ist durch das rosafarbene Fünfeck verdeutlicht. Die Phosphor-Atome sind in der abgebildeten Perspektive nicht zu sehen, da sie sich hinter den $\{WO_6\}^-$ Oktaedern befinden. b) Die Symmetrie bestimmt die Position des $[XM_6O_{24}]^{n-}$ Anions auf einer dreifachen Rotationsachse, da das Anion eine innere D_{3h} Symmetrie besitzt. Es ist zu beachten, dass Kristalle keine fünffachen Rotationsachsen besitzen und dieses Beispiel sich auf die interne Symmetrie eines z.B. Multi-Domänen Proteins bezieht (wobei die Domänen in diesen Beispiel durch Hühnereiweis-Lysozym dargestellt werden). Da sich das POM auf einer dreifachen Drehachse befindet, steht es mit drei Proteinmolekülen in Wechselwirkung. Die C_3 Symmetrie des Anderson-Evans-Anions ist durch das rosafarbene Dreieck verdeutlicht. Hühnereiweiß-Lysozym dient in dieser Abbildung als Anschauungsbeispiel für das Protein (grüner Cartoon). Farbschema: dunkelblau, M; hellblau; Wolfram; grün, X; rot, Sauerstoff. Punktgruppen und Symmetriesymbole sind in der Referenz [114] näher erklärt.

Das Potential des Hexawolframatotellurats als vielversprechendes Kristallisationsadditiv

Während der Suche nach einem passenden Kristallisationsadditiv wurden einige POM-Archetypen bezüglich ihrer Wirkung auf die Kristallisation von Proteinen hin getestet [41, 70, 71, 72]. Die Ergebnisse zeigten, dass das

Anderson-Evans-POM Hexawolframatotellurat $[TeW_6O_{24}]^{6-}$ (TEW) das vielversprechendste Kristallisationsadditiv unter den POMs war. Das POM ermöglichte die Kristallisation von zwei bis dato strukturell unbekannten Proteinen, der pilzlichen Tyrosinase aus *Agaricus bisporus* (*Ab*PPO4) [70, 115] und der Auronsynthase aus *Coreopsis grandiflora* (*Cg*AUS1) [72, 102]. Zudem führte die TEW-vermittelte Kristallisation des Modellproteins Hühnereiweiß-Lysozym (HEWL) zu einer vorher nicht bekannten Kristallform dieses Proteins [71]. Während der Kristallisation der Nukleotid-Bindedomäne des Hitzeschockproteins HSP70 mit TEW als Additiv induzierte das POM zwei unterschiedliche Kristallpackungen, wobei eine davon sehr ungewöhnlich war [76]. Die Struktur des TEWs bzw. des Anderson-Evans-POMs wurde bereits beschrieben (Abb. 1b). Es gibt jedoch zwei Arten der Anderson-Evans-Struktur, den nicht-protonierten A-Typen, wo das Heteroatom X sich in seiner höchsten Oxidationsstufe befindet, $[X^{n+}M_6O_{24}]^{(12-n)-}$ (M = Mo oder W; X = Te^{VI}, I^{VII}), und den protonierten B-Typen, der bis zu sechs Protonen an seine μ_3-O-Atome bindet, wobei das Heteroatom eine niedrige Oxidationsstufe besitzt, $[X^{n+}(OH)_6M_6O_{18}]^{(6-n)-}$ (M = Mo oder W; X = Cr^{III}, Fe^{III} (Abb. 16).

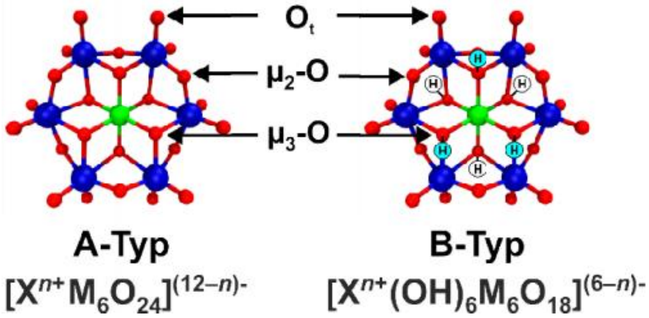

Abb. 16 Kugel-und-Stab-Modelle der A- und B-Form der Anderson-Evans-Struktur. Die bis zu sechs Protonen der B-Form, die an μ_3-O-Atomen gebunden sind, sind durch die Aufschrift „H“ gekennzeichnet (Protonen in weißen Kreisen zeigen in die Zeichenebene hinein, während die in türkisen Kreisen aus der Zeichenebene hinaus zeigen). Die unterschiedlichen Koordinationsmodi der Sauerstoff-Atome sind ebenfalls dargestellt. μ_3-O-Atome sind dreifach überbrückende Sauerstoff-Atome, die das Heteroatom und zwei Addenda-Atome miteinander verbinden. μ_2-O-Atome sind zweifach überbrückende Sauerstoff-Atome, die zwei Addenda-Atome miteinander verbinden. O_t-Atome sind terminale Sauerstoff-Atome, die paarweise an jedes Addenda-Atom gebunden sind. Farbschema: dunkelblau, M; grün, X; rot, Sauerstoff.

TEW ist ein A-Typ Anderson-Evans-Polyoxowolframat und erfüllt die wichtigsten Voraussetzungen eines Kristallisationsadditives, nämlich eine hohe Löslichkeit und Stabilität unter den üblichen Kristallisationsbedingungen und verfügt über die Fähigkeit, mit Proteinen zu wechselwirken, ohne dabei ihre strukturelle Integrität zu stören [40]. Die Synthese von TEW ist relativ unkompliziert und besteht im Grunde genommen aus dem Ansäuern einer wässrigen Lösung (pH ~ 5), die Na_2WO_4 und $Te(OH)_6$ in einem molaren Verhältnis von 6:1 enthält [115]. Die Lösung wird anschließend auf 110°C erhitzt, bis nur noch dreiviertel des Anfangsvolumens vorhanden sind, ehe das Endprodukt auskristallisiert wird (durch langsame Verdampfung bei Raumtemperatur). TEW wird normalerweise mittels Röntgenbeugung und/oder Infrarotspektroskopie (IR-Spektroskopie) sowie der Elementaranalyse identifiziert und charakterisiert. TEW kristallisiert triklin in der Raumgruppe P1 mit den Einheitszell-Parametern a ~ 10.3 Å, b ~ 10.6 Å, c ~ 11.1 Å, α ~ 91°, β ~ 115° und γ ~ 105° [116]. Das IR-Spektrum von TEW zeigt einen Fingerprint-Bereich, der charakteristisch für das Wolframgerüst der Anderson-Evans-Struktur ist. Streckschwingungen der terminalen W=O-Einheiten beobachtet man bei den Wellenzahlen ~ 952 und ~ 940 cm^{-1}. Das Spektrum zeigt Peaks bei ~ 884 cm^{-1} und im Wellenzahlbereich ~ 470 - 750 cm^{-1}, die den asymmetrischen bzw. symmetrischen Deformationsschwingungen der W-O-W- und der W-O-Te-Einheiten zugeschrieben werden [117]. Weitere Methoden, die zur Charakterisierung von TEW verwendet werden können, sind die Elementaranalyse, die ^{183}W-Kernspinresonanzspektroskopie (^{183}W-NMR-Spektroskopie) und die Massenspektrometrie. Abgesehen von dem jüngst entdeckten Einsatz des TEWs in der Proteinkristallographie wird das Anderson-Evans-POM nur als anorganischer Baustein für die Synthese organisch-anorganischer-Hybrid-POMs verwendet [118, 119, 120, 121, 122]. Zu diesem Zweck werden drei bis sechs Protonen der B-Typ-Struktur durch ein oder zwei Triol-Liganden ($RC(CH_2OH)_3$), R = organische Gruppe) ersetzt. Es gibt mehrere Argumente, die dafür sprechen, dass TEW ein potentes Kristallisationsadditiv ist, wobei es in mancher Hinsicht häufig verwendeten Kristallisationsadditiven überlegen ist:

TEW als Phasing-Werkzeug

TEW kann wie andere POWs und größere Metall-Cluster zur Lösung des ‚Phasenproblems‘ verwendet werden, wobei es, wie vorher bereits diskutiert, wesentliche Vorteile mit sich bringt. Die sechs anomal streuenden Wolfram-Atome sorgen dafür, dass TEW mehr als geeignet für das anomale Phasing ist. Da die L-I-Absorptionskante des Wolframs bei 1.02 Å liegt, kann das TEW ganz bequem zum Phasing mittels der SAD-Methode verwendet werden, selbst wenn die Daten an einer Röntgenquelle, deren Wellenlänge man nicht einstellen kann, gesammelt wurden. Dies ist ein wichtiger Vorteil gegenüber den meisten anomalen Streuern, da sich die Absorptionskanten der meisten anomal streuenden Atome bei entfernteren Wellenlängen befinden, weswegen man bei der Verwendung solcher Atome auf Röntgenquellen mit einstellbarer Wellenlänge angewiesen ist.

Chemische Eigenschaften des TEW, die für die Proteinkristallisation von Vorteil sind

TEW ist in wässrigen Lösungen gut löslich, was äußerst wichtig für dessen Verwendung in der Kristallisation ist, da Additive meistens in großem Überschuss (im Vergleich zur Proteinkonzentration) verwendet werden. Die Wasserlöslichkeit des Natriumsalzes von TEW, $Na_6[TeW_6O_{24}]$, beträgt ~ 100 mM. Andere bekannte POM-Archetypen wie die Wells-Dawson-Struktur sind generell deutlich weniger wasserlöslich mit Löslichkeitswerten, die sich hauptsächlich im Bereich von 2-10 mM bewegen. Informationen über die Löslichkeit von POMs findet man in [123]. Die Löslichkeit eines POMs kann durch die Wahl eines geeigneten Gegenkations (z.B. H^+, Na^+, K^+, etc.) verändert werden [124]. Die freien POM-Säuren sind mitunter instabil und sollten nicht ins Auge gefasst werden. TEW ist in einem pH-Wertbereich von 4.5 - 7.5 für mehrere Wochen bei 4 bis 20°C (weitere Temperaturen wurden nicht getestet) stabil [70, 71, 102, 115]. Die Struktur des TEWs bleibt in der Kristallisationslösung erhalten. Dies wurde anhand von Kristallstrukturen von Protein-TEW-Komplexen, die bei unterschiedlichen pH-Werten kristallisiert wurden, gezeigt. In dieser Hinsicht ist TEW anderen POMs gegenüber deutlich überlegen, da insbesondere Keggin- und Wells-Dawson-Anionen, die nur im sauren pH-Wertbereich stabil sind, bei neutralen oder höheren pH-Werten teilweise bis vollständig zerfallen [32]. Laut der PDB wurden die meisten Proteine in einem pH-Wertbereich von 4-9 kristallisiert. Die pH-Stabilität des TEWs deckt diesen pH-Bereich weitgehend ab, was die Eignung dieses POMs als Kristallisationsadditiv weiter bekräftigt.
TEW besitzt, wie die meisten POMs, eine relativ hohe negative Ladung, weshalb es in der Lage ist, elektrostatisch mit Proteinen zu wechselwirken. Da die Ladung von 6- über einen relativ großen Bereich (Größe des POMs) verteilt ist, kann das TEW mit großen Proteinbereichen interagieren. Dies bedeutet, dass das TEW mit mehreren Aminosäuren gleichzeitig interagieren kann, was ein Vorteil gegenüber anderen Additiven wie z.B. kleinen Molekülen oder Ionen darstellt, welche aufgrund ihrer kleineren Größe und Ladung deutlich stärker in der Anzahl der möglichen Wechselwirkungen eingeschränkt sind. Die Wechselwirkungen zwischen dem Protein und dem Kristallisationsadditiv darf nicht zur Präzipitation oder Denaturierung des Proteins während der Kristallisation führen. Röntgenstrukturanalysen und SDS-PAGE-Experimente verschiedener TEW-Protein-Komplexe (Hühnereiweiß-Lysozym, verschiedener Polyphenoloxidasen, Auronsynthase und humanes Serumalbumin) zeigten, dass die strukturelle Integrität der Proteine erhalten blieb, da keine signifikanten Konformationsänderungen des Proteins in Anwesenheit des TEWs beobachtet wurden [41, 70, 71, 102]. Es gibt jedoch POMs, insbesondere solche, die eine starke Lewis-Säure in ihrer Struktur besitzen, die Proteine hydrolytisch spalten, weshalb sie als künstliche Proteasen untersucht werden. Ein Beispiel für solch eine künstliche Protease ist das dimere POW $[Ce(PW_{11}O_{39})_2]^{10-}$, das in Lösung zum monomeren und hydrolytisch aktiven $[Ce(PW_{11}O_{39})]^{3-}$ dissoziiert [125]. Aufgrund der hohen Lewis-Acidität des Ce^{IV}-Atoms spaltet das Monomer das Hühnereiweiß-Lysozym regioselektiv an mehreren Stellen. Die meisten der hydrolytisch aktiven POMs sind Keggin-, Wells-Dawson- oder Lindqvist-Strukturen, die anstelle eines oder mehrerer Addenda-Atome ein hydrolytisch aktives Metall besitzen (Abb. 17a und 17b) [126]. Im Gegensatz zu diesen POMs können die Addenda-Atome des Anderson-Evans-POMs nicht durch andere Metalle als Molybdän oder Wolfram ersetzt werden. Deshalb können Metalle, die eine hohe Lewis-Acidität besitzen, nur als zentrales Heteroatom in die Struktur eingebaut werden. Aufgrund der planaren Form des scheibenförmigen Anderson-Evans-POMs wird das zentrale Heteroatom jedoch von den sechs Addenda-Atomen gut abgeschirmt (Abb. 17c). Unter diesen Umständen ist das Heteroatom der Anderson-Evans-Struktur kaum in der Lage, direkt mit dem Protein oder insbesondere mit dessen Rückgrat (Proteinhauptkette) zu wechselwirken. Diese abschirmende Wirkung wurde für unterschiedliche Anderson-Evans-Polyoxomolybdate, die teilweise starke Lewis-Säuren als Heteroatom besitzen ($FeMo_6$, $MnMo_6$, $GaMo_6$, $CrMo_6$), nachgewiesen, da keine der untersuchten Strukturen hydrolytisch aktiv war [118, 119, 127, 128]. Deshalb scheint der Anderson-Evans-Archetyp allgemein eines der

sichersten POMs im Hinblick auf die Aufrechterhaltung der strukturellen Integrität des Proteins zu sein. Diese Eigenschaft stärkt weiter die Eignung dieses POMs als Kristallisationsadditiv.

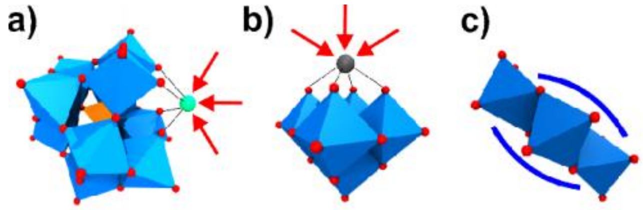

Abb. 17 Struktureller Vergleich zwischen hydrolytisch aktiven POMs und TEW. a) Struktur des proteolytisch-aktiven Keggin-Anions $[Ce(PW_{11}O_{39})]^{3-}$. b) Struktur des proteolytisch-aktiven Lindqvist-Anions $[Zr(W_5O_{18})]^{2-}$. In beiden Fällen ist das hydrolytisch-aktive Metallion (Ce^{IV} und Zr^{IV}, hellgrüne bzw. graue Kugel) gut zugänglich (durch rote Pfeile verdeutlicht), weshalb die jeweiligen POMs direkt mit einem Protein interagieren und es spalten können. Es sei darauf hingewiesen, dass die Lösungsmittelmoleküle, die sowohl an das Ce^{IV} als auch an das Zr^{IV} koordinieren, deutlichkeitshalber weggelassen wurden (Koordinationszahl = 7-8). c) Die Struktur von TEW $[TeW_6O_{24}]^{6-}$ ist zum Vergleich dargestellt. Ein hydrolytisch-aktives Metall kann nur als Heteroatom in die Anderson-Evans-Struktur eingebaut werden. Das Heteroatom (nicht sichtbar in der Perspektive der Abbildung) in der Anderson-Evans-Struktur ist jedoch durch das POM-Gerüst gut abgeschirmt (durch blaue Bögen verdeutlicht) und somit wird eine direkte Wechselwirkung des Heteroatoms mit einem Protein behindert. Farbschema: blau, Wolfram; orange, Phosphor; hellgrün, Cer; grau; Zirkonium; rot, Sauerstoff.

Vorteilhafte Größe, Form und Symmetrie des TEW für die Proteinkristallisation

Die besondere Form des Anderson-Evans-POMs birgt noch weitere Vorteile. Wie zuvor beschrieben können POMs als eine Art Abstandhalter zwischen den von ihnen ‚verbrückten‘ Proteinmolekülen wirken (Abb. 14). Aufgrund seiner Scheibenform besitzt die Anderson-Evans-Struktur eine flache und breite Seite und kann somit sowohl einen kleinen als auch großen Abstand zwischen ‘verbrückten’ Proteinmolekülen schaffen (Abb. 18). Je nach Ausrichtung variiert der Abstand zwischen den TEW-‚verbrückten‘ Proteinmolekülen zwischen ~ 6 und ~ 14 Å. Diese Flexibilität in den TEW-vermittelten Protein-Protein-Kontakt-Abständen könnte sich positiv auf den Kristallisationsprozess auswirken (erhöhte Freiheitsgrade bzgl. der ‚Verbrückung‘ von Proteinmolekülen). Wie bereits besprochen kann die Symmetrie eine wichtige Rolle in der POM-vermittelten Proteinkristallisation spielen, indem sie die Bindestelle und das Bindeverhalten der POMs dirigiert. Abb. 15 zeigte als Beispiel die hypothetische Positionierung des Anderson-Evans-Anions auf einer kristallographischen, dreifachen Drehachse. Dieses Szenario wurde jedoch noch nicht experimentell beobachtet. In der Kristallstruktur der pilzlichen Tyrosinase *Ab*PPO4 mit TEW befinden sich aber zwei TEW-Moleküle auf derselben kristallographischen Zweifachachse und wechselwirken jeweils mit zwei Proteinmolekülen [70]. Die approximative, innere D_{3d} Symmetrie von TEW, die drei C_2-Achsen als Symmetrieelemente besitzt, ist kompatibel mit der Symmetrie des Proteinkristalls, welcher monoklin in der Raumgruppe *C2* kristallisierte. In den restlichen Proteinkristallstrukturen, die TEW enthalten, befinden sich die TEW-Moleküle abseits von Symmetrieelementen, interagieren in den meisten Fällen jedoch trotzdem mit mehreren Proteinmolekülen.

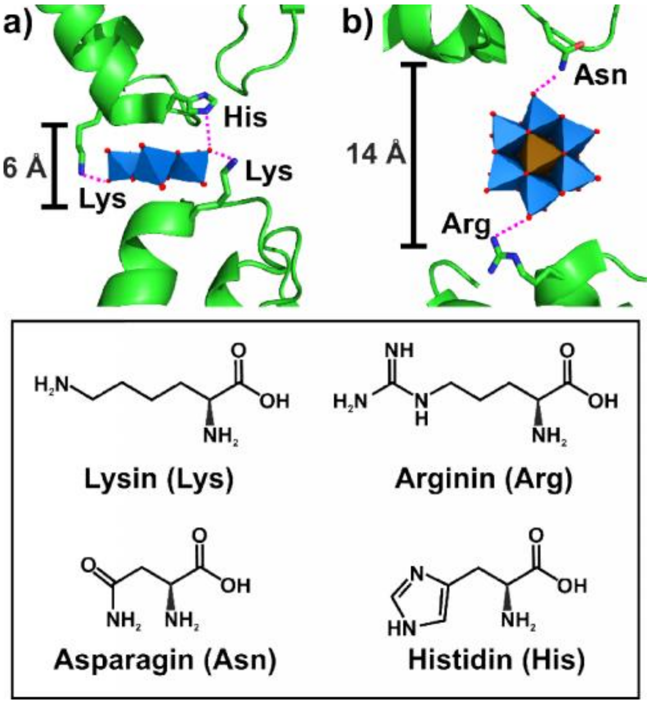

Abb. 18 ‚Verbrückung' von Proteinmolekülen durch unterschiedlich ausgerichtete TEW-Moleküle. a) Zwei Moleküle von *Ab*PPO4 (grüner Cartoon, PDB-Eintrag: 4OUA) sind durch ein TEW-Molekül miteinander 'verbrückt'. Das TEW ist horizontal (flache Seite) zwischen den Proteinmolekülen positioniert, was zu einem kleinen Protein-Protein-Abstand von ~ 6 Å führt. b) Zwei Moleküle des Hühnereiweiß-Lysozyms (grüner Cartoon, PDB-Eintrag: 4PHI) sind durch ein TEW-Molekül miteinander 'verbrückt'. Das TEW ist vertikal (breite Seite) zwischen den Proteinmolekülen positioniert, was zu einem relativ großen Protein-Protein-Abstand von ~ 14 Å führt. Aus Gründen der Übersicht ist jeweils nur ein Teil der TEW-Protein-Wechselwirkungen (rosa, gestrichelte Linien) angezeigt, wobei die wechselwirkenden Aminosäuren in der Stab-Darstellung abgebildet sind. Zusätzlich sind die Strukturformeln der Aminosäuren angegeben. Farbschema: hellblau, Wolfram; braun, Tellur; blau, Stickstoff; rot, Sauerstoff.

Die Fähigkeit des TEW, eine heterogene Proteinkristallisation zu induzieren

Während der Kristallisation der pilzlichen Tyrosinase *Ab*PPO4 verursachte TEW eine kristallographische Besonderheit. Tyrosinasen sind Enzyme, die Mono- und Diphenole zu den entsprechenden Chinonen umsetzen, welche anschließend über nicht-enzymatische Reaktionen zu Melanin weiterreagieren. Pilzliche Tyrosinasen, wie *Ab*PPO4, können in einer prämaturen, inaktiven (latenten) Form und einer aktiven Form existieren [70]. In der latenten Form des Enzyms wird das katalytisch aktive Zentrum, welches das binukleare Kupferzentrum enthält, von der enzymeigenen C-terminalen Domäne abgedeckt. Dadurch wird verhindert, dass Substrate (Mono- und Diphenole) in das aktive Zentrum gelangen, was zur Latenz des Enzyms führt. Deshalb geht man davon aus, dass eine noch unbekannte Protease den C-terminalen Teil des Enzyms abspaltet, wodurch das aktive Zentrum für die Substrate zugänglich und das Enzym aktiviert wird. Um nicht nur den katalytischen, sondern auch den Reifungsprozess (Aktivierung) dieses Enzyms zu studieren, bestand ein großes wissenschaftliches Interesse an den Kristallstrukturen sowohl der latenten (64 kDa) als auch der aktiven Form (44 kDa) dieser Enzymklasse. Die Kristallstruktur von *Ab*PPO4 konnte damals erst durch die Verwendung von TEW als Kristallisationsadditiv gewonnen werden, wobei das POM unerwarteterweise die Kristallisation sowohl des latenten als auch aktiven Enzyms in Form eines Heterodimers induziert hatte [70, 115]. Beide Enzymformen waren in einem einzigen Kristall vereint und somit hatte das TEW ‚zwei Fliegen mit einer Klappe geschlagen'. Dies ist nicht nur besonders bemerkenswert aufgrund der Tatsache, dass die latente und die aktive Form des Enzyms keine biologischen Dimere bilden, sondern weil die Kristallisation nach Homogenität strebt, um auf effiziente Weise einen hochgeordneten Einkristall zu bilden. Der Aufbau eines Kristalls funktioniert am besten, wenn man nur eine Art von Baustein (eine Art von Protein oder eines biologischen Oligomers) verwendet, da andere größere und/oder kleinere proteinogene Verunreinigungen (z.B. wie in diesem Fall die aktive Form) den Aufbau des Kristallgitters stören und somit den Kristallisationsvorgang behindern können (Abb. 19).

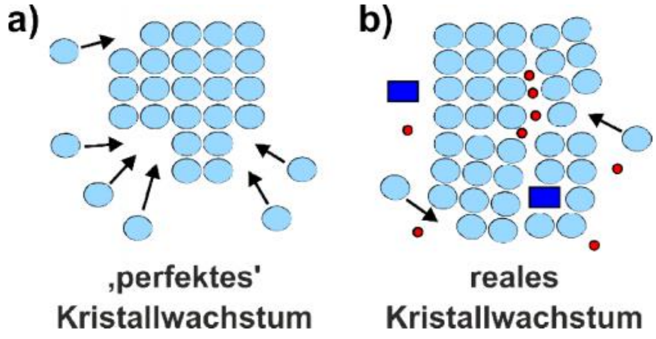

Abb. 19 Schematische Darstellung des Kristallwachstums. a) Ein nicht reales, ‚perfektes‘ Kristallwachstum. Dieses ‚perfekte‘ Kristallwachstum wäre nur möglich, wenn man eine hochreine Lösung, die nur eine einzige Sorte/Variante des Zielproteins (hellblaue Kugeln) und keine anderen das Kristallwachstum störenden Elemente oder Verunreinigungen enthält, hätte bzw. erzeugen könnte. In diesem Szenario könnten sich die Proteinmoleküle perfekt ausrichten und sich im dreidimensionalen Raum (in der Abbildung zweidimensional) zu einem ‚perfekten Kristall‘ anordnen. Diese Abbildung soll verdeutlichen, warum die Kristallisation nach Homogenität strebt. b) Ein reales Kristallwachstum mit Mosaikstruktur (Mosaizität). Dieses Kristallwachstum entsteht in Lösungen, die sowohl kleine (rote Kugeln) als auch große (dunkelblaue Rechtecke) Verunreinigungen enthalten. Selbst die Anwesenheit kleiner Verunreinigungen sorgt für Fehlstellen und Mängel im wachsenden Kristall, welche zu schief oder falsch ausgerichteten ‚Kristallblöcken‘ (= die Mosaizität beschreibt den Grad solcher Fehlausrichtungen ‚kristallographischer Blöcke‘) führt. Trotz dieser Mängel können sich qualitativ hochwertige Kristalle bilden, solange sie sich in Grenzen halten. Sobald die durch die Verunreinigungen entstehenden Mängel bzw. Fehlstellen zu groß werden, wird der Kristall aufgrund der zu hohen Mosaizität für das Röntgenbeugungsexperiment unbrauchbar. Da bei der Kristallisation von *Ab*PPO4 die Kristallisationslösung nur die latente Form des Enzyms enthalten sollte, stellte die Anwesenheit der aktiven Form eigentlich eine große, proteinogene Verunreinigung dar. Deshalb war die Wahrscheinlichkeit, dass die aktive Form die Kristallisation der latenten Form stark beeinträchtigt, größer als die, dass sich ein kristallisierbares Dimer bildet.

Diese besondere Anordnung war nur in der Anwesenheit von TEW möglich, da eine Seite jedes Heterodimers über einen TEW-vermittelten Kristallkontakt, der durch zwei TEW-Molekülen stabilisiert wird, mit einem Symmetrie-verwandten Heterodimer verbunden ist. Dabei bindet ein TEW-Molekül an die latenten Einheiten von zwei miteinander ‚verbrückten‘ Heterodimeren, während das zweite TEW-Molekül entsprechend die aktiven Einheiten miteinander ‚verknüpft‘. Die TEW-Moleküle befinden sich auf einer kristallographischen, zweifachen Drehachse. Die andere Seite jedes Heterodimers ist über einen üblichen Protein-Protein-Kontakt mit dem nächsten, Symmetrie-verwandten Heterodimer verbunden (Abb. 20). Dieses Muster wiederholt sich im gesamten Kristall und baut diesen somit auf. Dieser besondere Fall zeigt, dass das TEW in der Lage ist, heterogene Kristallisation (= die Kristallisation von mindestens zwei unterschiedlichen Proteinen in einer Kristallstruktur) zu induzieren. Dies könnte von großer Wichtigkeit in Hinblick auf die Kristallisation von Multi-Domänen Strukturen oder größeren heterogenen, molekularen Komplexen sein.

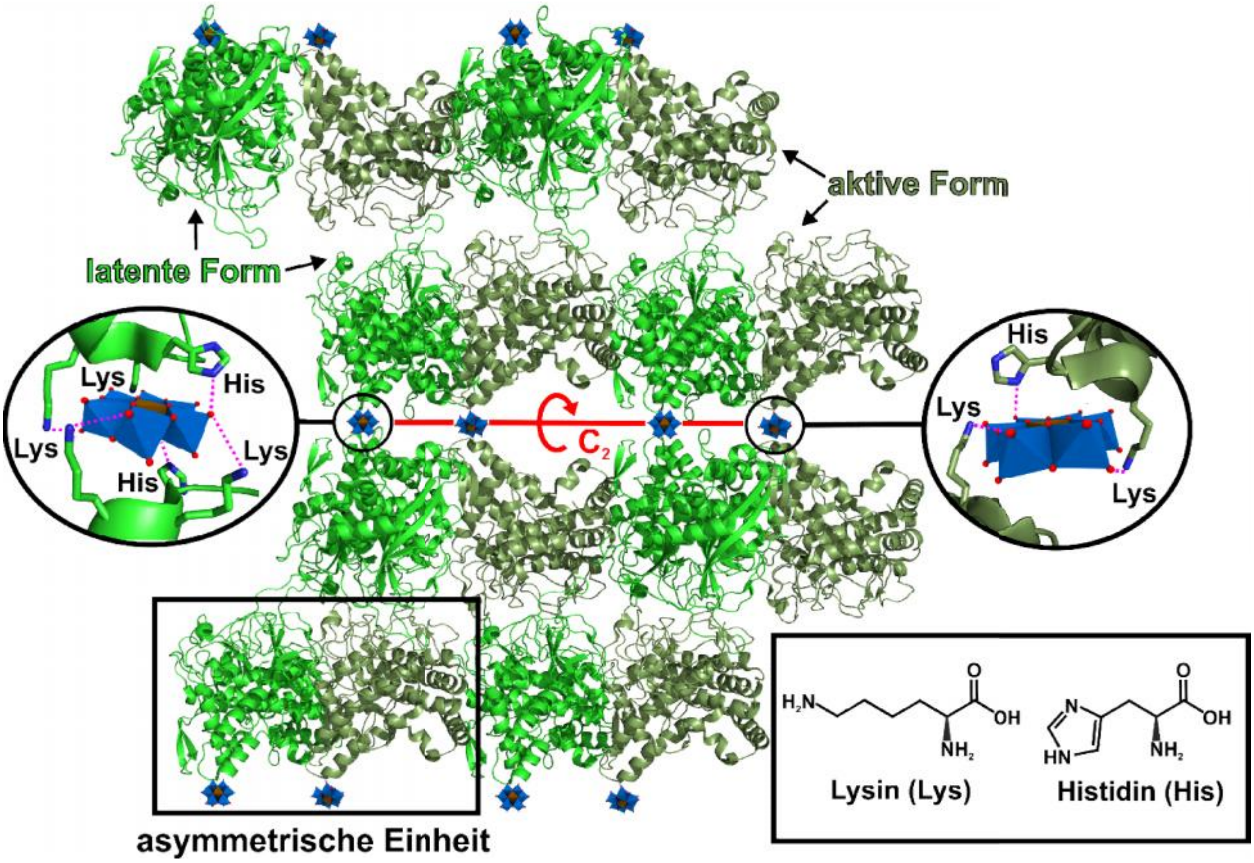

Abb. 20 TEW-vermittelte heterogene Kristallisation der latenten und aktiven Form von *Ab*PPO4. Ein Ausschnitt der Kristallpackung des *Ab*PPO4-TEW-Komplexes ist dargestellt, welcher zeigt, dass sowohl die latente (hellgrüner Cartoon) als auch die aktive Form (dunkelgrüner Cartoon) des Enzyms in ein und demselben Kristall enthalten sind. Jedes TEW-Molekül interagiert mittels elektrostatischer Wechselwirkungen mit Lysin- (Lys) und Histidinresten (His) von zwei latenten bzw. aktiven Einheiten (siehe runden Einschub). Eine Seite jedes Heterodimers wird über zwei TEW-Moleküle mit dem nächsten Dimer ‚verbrückt', während die andere Seite mittels üblicher Protein-Protein-Wechselwirkungen mit dem nächsten Dimer in Verbindung steht. Die TEW-Moleküle liegen auf einer kristallographischen, zweifachen Drehachse (rot eingezeichnet in der Mitte der Abbildung). Die TEW-Moleküle sind als Polyeder dargestellt, während die wechselwirkenden Lysin- und Histidinreste in der Stab-Darstellung angezeigt werden. Zudem sind die Strukturformeln der involvierten Aminosäuren angegeben. Farbschema: blau, Wolfram; braun, Tellur; dunkelblau, Stickstoff; grün, Kohlenstoff; rot, Sauerstoff.

Der Einfluss der geometrischen und funktionellen Flexibilität des TEW auf die Proteinkristallisation

Während der Kristallisation von *Cg*AUS1 mit TEW kam es zu einer unerwarteten kovalenten Bindung zwischen dem Protein und einem TEW-Molekül [72]. Zwei Wolfram-Atome des TEW-Moleküls bindeten kovalent an die Carboxyl-Sauerstoff-Atome einer Glutaminsäure, wodurch die $[TeW_6O_{24}O_2(Glu)]^{7-}$-Einheit ($O_2$ = Carboxyl-Sauerstoff-Atome der Glutaminsäure = Glu) entstand. Dies war der erste Fall einer kovalenten POM-Protein-Bindung, wo das fertige POM verwendet wurde und sich nicht erst *in situ* während der Kristallisation bildete. Interessanterweise hat die Ausbildung dieser kovalenten Bindung auch strukturelle Auswirkungen auf das TEW, welches sich strukturell umordnete, sodass die eigentlich planare Anderson-Evans-Struktur in eine außergewöhnliche, gekrümmte Struktur überging (Abb. 21a-c). Der Mechanismus, der zu der kovalenten Bindung führte, ist nicht bekannt, jedoch geht man davon aus, dass die proteinogene Umgebung des TEWs die Bindung sterisch forcierte. Das Polyanion befindet sich in einer schmalen, stark positiv geladenen Spalte, wo es mit den es umgebenden Aminosäuren stark wechselwirkt (Abb. 21d). Die kristallographischen Ergebnisse deuten an, dass das TEW in der Lage war, sich strukturell dem Protein anzupassen, da die Gesamtstruktur des Proteins unverändert geblieben ist. Somit ist die scheibenförmige Anderson-Evans-Struktur unter bestimmten Umständen in der Lage, erstaunliche konformationelle Veränderungen einzugehen, jedoch ist die Triebkraft, die dahinter steckt, unklar. Die Fähigkeit von TEW, kovalente Bindungen mit Aminosäuren auszubilden, könnte zur kristallisationsfördernden Wirkung dieses Moleküls beitragen, indem es beispielsweise flexible Proteindomänen, die die Kristallisation beeinträchtigen, kovalent fixiert. Des Weiteren könnte TEW vereinzelt Proteinmoleküle kovalent miteinander verbrücken, was zu noch stabileren Kristallkontakten führen würde.

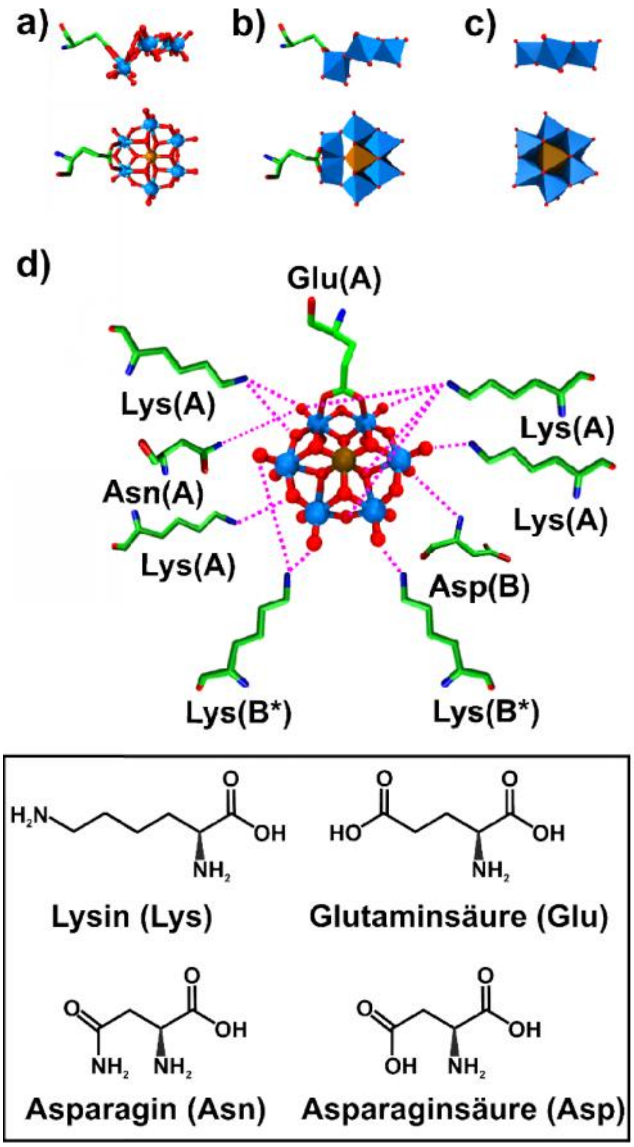

Abb. 21 Kovalente Bindung zwischen TEW und *Cg*AUS1. a) Die kovalente Bindung zwischen TEW und der Glutaminsäure (Glu) ist abgebildet. Die Bindung führt zu einer noch nie zuvor beobachteten, Aminosäure-gebundenen und gekrümmten Anderson-Evans-Struktur (oben: Seitenansicht, unten: Ansicht von oben). b) Dieselbe Abbildung wie in a), aber das TEW ist in der Polyeder- anstelle des Kugel-Stab-Modells angezeigt. c) Die normale Anderson-Evans-Struktur ist zum Vergleich abgebildet. d) Übersicht über alle direkten TEW-Protein-Wechselwirkungen innerhalb der TEW-Bindestelle (Lösungsmittel-vermittelte Wechselwirkungen sind der Deutlichkeit halber weggelassen). Es ist darauf hingewiesen, dass die Wasserstoffbrückenbindung zwischen dem POM und der Asparaginsäure (Asp) von der Aminogruppe des Peptidrückgrats (primäres Amin) ausgeht, wohingegen die restlichen Aminosäuren über ihre Seitengruppen mit dem TEW wechselwirken. Die Klammern geben an, von welchen Proteinmolekülen die wechselwirkenden Aminosäuren kommen, da das Protein als Dimer (zwei Proteinketten, A und B) kristallisiert wurde. Ein Sternchen deutet an, dass die Aminosäure von einem Proteinmolekül herstammt, welches sich in einer benachbarten asymmetrischen Einheit befindet. Die Strukturformeln der wechselwirkenden Aminosäuren sind zusätzlich abgebildet. Farbschema: blau, Wolfram; braun, Tellur; grün, Kohlenstoff; dunkelblau, Stickstoff; rot, Sauerstoff.

Die Fähigkeit von TEW, die Kristallqualität zu verbessern

TEW war in einigen Fällen in der Lage, die Kristallqualität von Proteinkristallen (im Vergleich zu den entsprechenden POM-freien Kristallen) zu verbessern. *Cg*AUS1 wurde in drei unterschiedlichen Kristallformen kristallisiert, zwei ohne und eine mit TEW [129]. Die Kristallisationsbedingungen aller Kristallformen waren im Wesentlichen dieselben mit der Ausnahme, dass die Ansätze ohne das Polyanion $MgCl_2$ anstatt TEW als Additiv beinhalteten. Die Kristalle, die TEW beinhalteten, waren von deutlich höherer Qualität, was zu einer Verbesserung der Auflösung von bis zu ~1.0 Å im Vergleich zu den TEW-freien Kristallen führte [72]. Die genaue Untersuchung und der Vergleich der Kristallstrukturen der drei Kristallformen zeigte, dass die Kristallkontakte in den TEW-Kristallen spezifischer als die in den Kristallen, die kein POM enthalten, sind [130]. Alle Kristallformen werden durch das gleiche kristallographische Dimer aufgebaut, jedoch unterscheidet sich die Anzahl dieser Dimere in den

asymmetrischen Einheiten (ASU) der jeweiligen Kristallform zum Teil deutlich. Die ASU des *Cg*AUS1-TEW-Kristalls besteht aus einem kristallographischen Dimer (zwei Proteinmoleküle), während die der TEW-freien Kristalle aus zwei bzw. vier Dimeren (vier bzw. acht Proteinmoleküle) bestehen. Aufgrund ihrer geringen Anzahl an Proteinmolekülen in der ASU besitzen die *Cg*AUS1-TEW-Kristalle eine höhere Symmetrie und somit ist ein kleineres Ensemble zum Aufbau dieser Kristalle im Vergleich zu den TEW-freien Kristallen notwendig (Abb. 22). Zwei TEW-Moleküle befinden sich in der Kristallstruktur von *Cg*AUS1-TEW und sorgen für neue Kristallkontakte, wobei ein Anion das kristallographische Dimer deutlich stabilisiert. Die TEW-vermittelten Kristallkontakte, insbesondere der Dimer-stabilisierende Kontakt, scheinen der Grund für die verbesserte Kristallqualität zu sein, da sie mit Abstand die stärksten Kristallkontakte innerhalb des Kristalls bilden (gemessen an der Kontaktfläche und Anzahl der Kontakt bildenden Aminosäuren). Deshalb bestimmen die TEW-vermittelten Kristallkontakte weitestgehend die Bildung des Kristalls, da sie dominierende Adhäsionsmodi zwischen den Proteinmolekülen zur Verfügung stellen [131]. Dies bedeutet, dass die Proteinmoleküle überwiegend über die TEW-gestützten Kontakte miteinander wechselwirken und assemblieren. Im Gegensatz dazu fehlt es in den Kristallen ohne TEW an bestimmenden bzw. bevorzugten Adhäsionsmodi, was zu mehr und vor allem unspezifischen Protein-Protein-Kontakten führt. Dadurch wechselwirken die Proteinmoleküle eher zufällig miteinander, da mehr gleichwertige Bindestellen (an der wachsenden Kristalloberfläche) zur Verfügung stehen, was letztendlich zu einer Vielzahl von Anordnungsfehlern und einer geringeren (long-range-) Periodizität führt, die für gut streuende Kristalle notwendig ist. Dasselbe könnte auch auf die pilzliche Tyrosinase zutreffen, da vor kurzem die Kristallstruktur der latenten Form ohne die Verwendung von TEW erhalten wurde [132]. Die neue Struktur hat eine deutlich geringere Auflösung als die, die mit Hilfe von TEW erhalten wurde (2.8 vs. 3.3 Å). Die TEW-haltige und die TEW-freie Struktur bzw. die zugrunde liegende Kristallpackung konnte aufgrund des unterschiedlichen Kristallinhaltes (nur latente Form vs. Heterodimer) nicht vernünftig verglichen werden [132]. Nichtsdestotrotz spielen in den oben angeführten Beispielen die TEW-vermittelten Kristallkontakte eine entscheidende Rolle für die Kristallqualität.

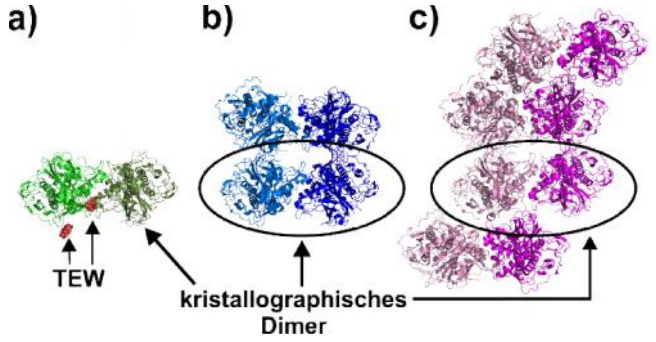

Abb. 22 Vergleich der asymmetrischen Einheiten der TEW-haltigen Kristallform mit den TEW-freien Kristallformen von *Cg*AUS1. a) Asymmetrische Einheit des *Cg*AUS1-TEW Kristalls. Die TEW-Moleküle sind als Cluster roter Kugeln dargestellt, wobei ein TEW-Molekül das kristallographische Dimer stabilisiert. b) Asymmetrische Einheit der TEW-freien Kristallform, die aus vier Proteinen besteht (zwei kristallographische Dimere). c) Asymmetrische Einheit der TEW-freien Kristallform, die aus acht Proteinen besteht (vier kristallographische Dimere). Alle drei Kristallformen bestehen aus demselben kristallographischen Dimer, wobei die unterschiedlichen Proteinmoleküle in unterschiedlichen Farbtönen dargestellt sind (grün/dunkelgrün, blau/dunkelblau, rosa/dunkelrosa).

Die Fähigkeit von TEW, einen deutlichen Entropiegewinn zur Proteinkristallisation beizusteuern

TEW wirkt sich deutlich auf die Lösungsmittelentropie, die die treibende Kraft der Kristallisation ist, aus. Deshalb kann TEW den Kristallisationsvorgang einiger Proteine energetisch begünstigen [130]. Wie bereits besprochen, wird die Kristallisation durch die Tatsache, dass Proteine aufgrund der unregelmäßigen Verteilung hydrophiler und hydrophober Bereiche auf ihrer Oberfläche nur eine geringe Anzahl an schwachen Kristallkontakten bieten, stark limitiert [6]. Die freie Kristallisationsenergie ΔG^{o}_{Krist} setzt sich sowohl aus enthalpischen (ΔH^{o}_{Krist}) als auch entropischen ($-T\Delta S^{o}_{Krist}$) Beiträgen zusammen und kann wie folgt beschrieben werden [133]:

$$\Delta G^{o}_{Krist} = \Delta H^{o}_{Krist} - T(S^{o}_{Protein} + S^{o}_{Lösungsmittel})_{Krist}$$

Damit es zur Kristallisation kommen kann, muss die Kristallisationsenergie ΔG^{o}_{Krist} negativ sein. Der enthalpische Term ΔH^{o}_{Krist} wird aufgrund der wenigen und schwachen intermolekularen Kristallkotakte im besten Fall nur leicht negativ. Erschwerend kommt hinzu, dass der Phasenübergang der Proteinmoleküle aus der Lösung, wo sie sehr beweglich sind, in ein rigides und geordnetes Kristallgitter die Proteinentropie ($S^{o}_{Protein}$) stark reduziert, da die

Proteinmoleküle ihre Freiheitsgrade (Beweglichkeit) verlieren. Dies führt zu einem für die Kristallisation sehr ungünstigen negativen $S^{o}_{Protein}$-Term. Deshalb muss dieser Entropieverlust durch die Lösungsmittelentropie $S^{o}_{Lösungsmittel}$ kompensiert werden. Die Lösungsmittelentropie steigt mit der Anzahl der aus der Hydratationshülle der Proteine freigesetzten Lösungsmittelmoleküle, die die Hülle während der Bildung von Protein-Protein-Kontakten (Kristallkontakte) verlassen. Nur wenn die gebildeten Kristallkontakte eine ausreichend hohe Lösungsmittelentropie $S^{o}_{Lösungmittel}$ herbeiführen (d.h. genügend Lösungsmittelmoleküle freigesetzt werden), die den Wert der Gesamtenergie der Kristallisation negativ macht, kann ein Kristall gebildet werden. Die Untersuchung der Kristallisation von drei Proteinen (*Cg*AUS1, *Ab*PPO4 und HEWL), die jeweils mit und ohne TEW kristallisiert wurden, zeigte, dass es in Anwesenheit von TEW und nach Ausbildung der TEW-stabilisierten Kristallkontakte zu einem deutlichen Zuwachs an Lösungsmittelentropie $S^{o}_{Lösungmittel}$ kommt [130]. Die Zunahme in der Lösungsmittelfreisetzung während der TEW-induzierten Kristallbildung und -packung ist deutlich höher als die, die durch die üblichen Additive (z.B. verschiedene kleine Anionen oder Kationen wie I^-, NO_3^-, Zn^{2+}, Y^{3+}, etc.) induziert wird. Dies ist, unter anderem, mit der Größe des TEWs und der damit assoziierten großen TEW-Protein-Kontaktfläche verbunden, da mehr Lösungsmittelmoleküle aus der Hydratationshülle des TEWs und des Proteins freigesetzt werden können (Abb. 23). Andere POMs könnten eine ähnliche Wirkung auf die Kristallisationsenergie haben, da der Gewinn an Lösungsmittelentropie hauptsächlich von der Größe des POMs und dessen Affinität zu Proteinen abhängt. Leider wurde dies noch nicht für andere POM-Protein-Systeme untersucht.

Aufgrund der hier diskutierten Eigenschaften ist TEW prädestiniert für die Verwendung als Kristallisationsadditiv, da es viele Vorteile im Vergleich zu anderen POM-Archetypen und gängigen Kristallisationsadditiven mit sich bringt. Deshalb werden Kristallographen von dessen künftiger Anwendung profitieren. Aufgrund der bereits erwähnten Erfolge in der Proteinkristallographie wurde TEW in den Markt eingeführt und ist nun bei Jena Bioscience (www.jenabioscience.com) als ‚Crystallisation-Screen' kommerziell erhältlich.

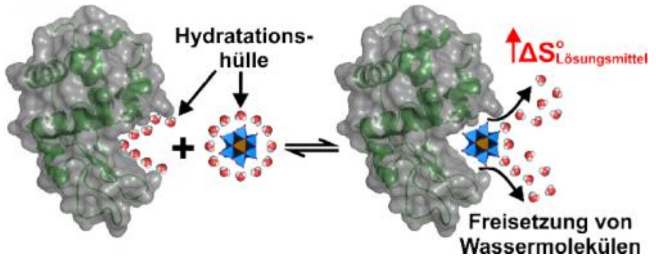

Abb. 23 Schematische Darstellung des Beitrages von TEW zur Lösungsmittelentropie der Kristallisation mittels der Freisetzung von Wassermolekülen. Bevor das Protein und TEW miteinander wechselwirken, sind beide von einer Hydrathülle (dargestellt durch kleine Wassermoleküle) umhüllt. Nach der Bindung des TEW sorgt die Freisetzung der Wassermoleküle aus der Hydratationshülle beider Moleküle (partielle Dehydratation) für einen Zuwachs in der Lösungsmittelentropie. Die Bindung eines zweiten Proteinmoleküls, die für eine weitere Freisetzung von Wassermolekülen und zur Vervollständigung des Kristallkontaktes führt, ist der Deutlichkeit halber weggelassen worden. Das Protein (Hühnereiweiß-Lysozym als Anschauungsbeispiel) ist als grüner Cartoon in Kombination mit einer Oberflächen-Darstellung (transparente, graue Oberfläche) angezeigt, wohingegen das TEW in der Polyeder-Darstellung abgebildet ist. Farbschema: blau, Wolfram; braun, Tellur; rot, Sauerstoff, weiß, Wasserstoff.

Hinweise zur praktischen Nutzung von Polyoxometallaten in der Proteinkristallisation

Wie wir in diesem Artikel zeigen konnten, stellen POMs eine vielversprechende Gruppe potentieller Kristallisationsadditive dar. Die meisten ihrer Eigenschaften und Funktionen können zu Gunsten des Kristallographen abgestimmt werden. Beispielsweise lässt sich die Löslichkeit der POMs durch die Wahl des Gegenkations ändern, die Ladung durch die passende Kombination der Addenda- und Heteroatome abstimmen und ihre Form und chemischen Eigenschaften mittels der Modifizierung ihrer Oberflächen (z.B. durch den Austausch eines Addenda-Atoms durch ein anderes Metall) manipulieren. Trotz der vielversprechenden Eigenschaften der POMs sollte man sie mit Vorsicht für die Kristallisation verwenden, da die meisten POMs ein unberechenbares Verhalten in Lösung zeigen. Die Chemie der POMs in wässriger Lösung ist sehr komplex und größtenteils abhängig vom verwendeten Addenda-Atom und pH-Wert. Generell bilden POMs mehrere Polyoxo-Spezies bei bestimmten pH-Wertbereichen aus, die dann in der Lösung koexistieren [134]. Eine pH-abhängige Instabilität bei neutralen oder basischen pH-Werten wurde bereits bei einigen POM-Archetypen beobachtet (z.B. zerfallen die Keggin- und die Wells-Dawson-Struktur in niedrigmolekulare Spezies), wodurch eine genaue Bestimmung der in der Lösung vorliegenden POM-Spezies erschwert wird. Dies stellt ein besonderes Problem im Hinblick auf ihre Verwendung in der Proteinkristallisation dar, da die meisten Proteine im neutralen pH-Bereich kristallisiert werden. Leider gibt es

keine (hoch)zuverlässigen Methoden zur schnellen Bestimmung der vorherrschenden POM-Spezies in Lösung ohne jegliche Vorkenntnisse über das Gleichgewicht zwischen Zusammensetzung und Abbau eines bestimmten POMs. Klassische chemische Ansätze wie die Elementaranalyse können wertvolle Informationen über die Zusammensetzung des POMs liefern. Die zuverlässigste und am häufigsten verwendete Methode zur Bestimmung der POM-Struktur ist die Einkristall-Röntgenstrukturanalyse. Die Kristallstruktur liefert jedoch nur strukturelle Informationen über die POM-Struktur im festen Zustand, was jedoch keine Garantie dafür ist, ob die beobachtete Struktur die in der Lösung vorherrschende Spezies darstellt. Das kristallisierte POM könnte auch nur die POM-Spezies in einer Lösung, die mehrere POM-Spezies beherbergt, darstellen, die am schnellsten bzw. am leichtesten auskristallisiert. Andere Methoden, die auch häufig zur Charakterisierung der POM-Struktur angewendet werden, sind die NMR-, UV/Vis- und IR-Spektroskopie, die Massenspektrometrie und die Röntgenkleinwinkelstreuung (SAXS). All diese Methoden liefern wertvolle strukturelle Informationen, jedoch reicht die Charakterisierung durch nur eine dieser Methoden häufig nicht zum vollständigen Strukturnachweis aus. Deshalb ist es am besten und zuverlässigsten, eine Kombination der genannten Methoden für die korrekte Bestimmung der POM-Struktur zu verwenden.

Ein weiterer Aspekt, der bedacht werden sollte, ist die Größe und Form des POMs, das man verwenden möchte. Zu große POMs oder POM-Archetypen werden die Bildung günstiger Protein-Protein-Kontakte eher stören, anstatt sie zu fördern bzw. zu stabilisieren, da sie vor allem bei der Kristallisation kleinerer Proteine nicht zwischen die Proteinmoleküle innerhalb eines bestimmten Kristallgitters passen werden. Das bis jetzt größte POM, das in der Röntgenstrukturanalyse verwendet wurde, ist das Preyssler-Anion $[NaP_5W_{30}O_{110}]^{14-}$ mit einer Größe von ~ 15 x 18 x 10 Å [105, 113]. Es muss jedoch erwähnt werden, dass dieses POM zum Phasing eines sehr großen Proteins verwendet wurde, der Riboflavinsynthase (~ 1 MDa). Zudem steht die Größe eines POMs in direkter Verbindung zu dessen Ladungsdichte, die wiederum eine entscheidende Rolle bei der Wechselwirkung des POMs mit Proteinen spielt. Wenn eine bestimmte Ladung über eine zu große POM-Oberfläche verteilt und somit verdünnt wird, dann verringert sich nicht nur die Ladungsdichte des POMs, sondern auch unter Umständen dessen Affinität zu Proteinen.

Wie zuvor diskutiert, gibt es auch hydrolytisch aktive POMs, die Proteine spalten. Während es bei POMs, die z.B. eine starke Lewis-Säure in ihrer Struktur tragen, offensichtlich ist, dass sie hydrolytisch aktiv sind bzw. sein könnten, sieht man es manchen anderen POMs leider nicht an, dass sie proteolytisch aktiv sind und somit vermieden werden sollten. Bekannte POMs wie beispielsweise das gerade erwähnte Preyssler-POM, die von der Zusammensetzung eher unauffällig sind, sind in der Lage, Proteine partiell zu denaturieren (z.B. humanes Serumalbumin) [93]. Deshalb sollte man die POMs auch diesbezüglich testen, bevor man sie für die Proteinkristallisation verwendet. Die SDS-PAGE oder die Circulardichroismus-Spektroskopie liefern Informationen über die Stabilität des Proteins in Anwesenheit des POMs. Es muss jedoch erwähnt werden, dass einige Kristallisationsansätze Proteasen mit Vorsatz beinhalten, um die Kristallisation von großen (Multi-Domänen) oder hochflexiblen Proteinen zu ermöglichen. Die Strategie dahinter ist es, hochflexible Loops proteolytisch zu entfernen (im Fall der flexiblen Proteine) und/oder große Proteine in kleinere Fragmente zu spalten (im Fall der Multi-Domänen Proteine), was (in beiden Fällen) die Kristallisierbarkeit des Proteins deutlich erhöht.

Der Kristallisationspuffer kann auch zu einem entscheidenden Punkt werden, da einige POM-Puffer-Kombinationen zu unerwünschten Ergebnissen führen können. Die Pufferkomponenten sollten chemisch nicht mit dem POM interferieren, da dies zu einer Veränderung der POM-Struktur und/oder Einschränkung der POM-Funktion führen kann. Einige Polyoxovanadate sind dafür bekannt, Komplexe mit Puffern wie z.B. dem TRIS-Puffer zu bilden [133]. Zudem kann der Puffer die Funktion des POMs einschränken, ohne dabei direkt mit diesem zu interagieren. Beispielsweise ändern Puffer, die flüchtige Komponenten beinhalten, den pH-Wert der Lösung mit der Zeit, was zur Dissoziation des POMs führen kann, da deren Stabilität stark pH-abhängig ist. Hohe Konzentrationen von salzhaltigen Puffern, die man für die Kristallisation einiger Proteine benötigt, sorgen für eine hohe Ionenstärke in der Lösung, die sich negativ auf die Wechselwirkung zwischen den POMs und den Proteinen auswirken kann. Der Grund dafür ist, dass es zu einem Konkurrenzkampf zwischen den POMs und den Anionen des Salzes um die (geladenen) Bindestellen an den Proteinmolekülen kommt.

Deshalb bedarf es bei der Verwendung von POMs in der Proteinkristallographie guter Vorausplanung und experimentellen Aufwands, um mögliche negative Auswirkungen der POMs auf das Protein und/oder den Kristallisationsprozess auszuschließen. Dies spart Zeit und Ressourcen (reines Protein, Kristallisationslösungen, Verbrauchmaterialien und vor allem den Aufwand des Kristallographen).

Schlussbemerkungen

Aufgrund der Vielseitigkeit der POMs in ihrer Struktur und physikochemischen Eigenschaften, die man gezielt abstimmen und fine-tunen kann, stellen sie nicht nur gute Phasing-Werkzeuge, sondern auch vielversprechende Kristallisationsadditive in der Proteinkristallographie dar. Die gezielte Anwendung einiger POMs führte zu aussichtsreichen Ergebnissen, da die POMs in der Lage waren, die Kristallisation einiger Proteine zu fördern und in einigen Fällen sogar nötig waren, um das Zielprotein zu kristallisieren. Dies geschah durch die Bildung und Stabilisierung von Kristallkontakten und/oder die Stabilisierung von Proteinkonformationen. In dieser Hinsicht war insbesondere das Anderson-Evans Polyoxowolframat sehr wirkungsvoll, da es einige Vorteile gegenüber anderen POM-Archetypen und häufig verwendeten Kristallisationsadditiven mit sich bringt. Ein erhöhter experimenteller Aufwand ist jedoch notwendig, um die Verwendung von POMs noch effizienter zu gestalten. Beispielsweise bedarf es eines noch besseren Verständnisses der POM-Protein- bzw. POM-vermittelten Protein-Protein-Wechselwirkungen, da es keine eindeutige Korrelation zwischen der Affinität eines POMs zu einem Protein und dessen strukturellen oder chemischen Charakteristika gibt. Zwar befanden sich die meisten POMs in Proteinbereichen, die ein positives elektrostatisches Potential aufwiesen, jedoch wurden POMs auch in elektrostatisch neutralen Proteinbereichen gefunden (polare oder hydrophobe Bereiche), obwohl dasselbe Protein über positiv geladene Regionen verfügte. Deshalb scheinen auch andere Faktoren eine wichtige Rolle bei den POM-Protein-Wechselwirkungen zu spielen, wie beispielsweise:

- Die Kompatibilität zwischen der Größe/Form des POMs und der Bindestelle am Protein.
- Der entropische Effekt aufgrund der partiellen Dehydratation der Hydratationshüllen des POMs und des Proteins, der die Wechselwirkung zwischen den beiden Molekülen fördert.
- Die Ladungsdichte und die chaotrope Natur einiger POMs, die sogar hydrophobe Wechselwirkungen mit dem Protein ermöglichen.

Darüber hinaus muss das Verhalten der POMs in wässrigen Lösungen genauer studiert werden. POMs neigen dazu, mehrere Kondensation-Hydrolyse-Gleichgewichtszustände (POM-Bildung und –zerfall) einzugehen, was zu einer Koexistenz mehrerer POM-Spezies in Lösung führt. Dies könnte zur Behinderung der Proteinkristallisation aufgrund der erhöhten Inhomogenität der Lösung (durch die Anwesenheit mehrerer POM-Spezies) führen. Nichtsdestotrotz könnten die jüngsten Erfolge der POMs in der Proteinkristallisation der Beginn einer erfolgreichen Zukunft sein, in deren Verlauf einige Bereiche der Chemie, insbesondere die, die auf den Input von 3D-Proteinstrukturen angewiesen sind (z.B. Strukturbiologie, Biochemie, Medizin und Pharmazie), von der Verwendung von POMs profitieren könnten.

Danksagung

Die Anfertigung dieses Lehrtextes wurde auf verschiedene Weise vom Fonds zur Förderung der wissenschaftlichen Forschung (FWF, P27534) und der Universität Wien gefördert. Die Autoren bedanken sich bei Nadiia Gumerova, PhD und Dipl.-Ing. Matthias Pretzler für das Korrekturlesen der englischen Version und Dr. Joscha Breibeck und Dr. Hans-Joachim Lunk für das Korrekturlesen der deutschen Version dieses Artikels.

Referenzen

1. Kendrew JC, Bodo G, Dintzis HM, Parrish RG, Wyckoff H, Phillips DC (1958) A three-dimensional model of the myoglobin molecule obtained by X-ray analysis. Nature 181:662–666

2. Perutz MF, Rossmann MG, Cullis AF, Muirhead H, Will G, North ACT (1960) Structure of hæmoglobin: a three-dimensional Fourier Synthesis at 5.5-Å resolution, obtained by X-Ray analysis. Nature 185:416–422

3. Drenth J (2007) Principles of protein X-ray crystallography, Springer-Verlag, New York

4. Goulding CW, Perry LJ (2003) Protein production in *Escherichia coli* for structural studies by X-ray crystallography. J Struct Biol 142:133–143

5. Walsh CT, Garneau-Tsodikova S, Gatto GJ (2005) Protein posttranslational modifications: the chemistry of proteome diversifications. Angew Chem Int Ed 44:7342–7372

6. Rupp B (2009) Biomolecular crystallography: principles, practice, and application to structural biology. Garland Science, New York

7. Eckert M (2012) Max von Laue and the discovery of X-ray diffraction in 1912. Ann Phys 524:83–85

8. Laue M (1913) Eine quantitative Prüfung der Theorie für die Interferenzerscheinungen bei Röntgenstrahlen. Ann Phys 346:989–1002

9. Bragg WH, Bragg WL (1913) The reflection of X-rays by crystals. Proc R Soc Lond Math Phys Eng Sci 88:428–438

10. Rice LM, Earnest TN, Brunger AT (2000) Single-wavelength anomalous diffraction phasing revisited. Acta Crystallogr Sect D Biol Crystallogr 56:1413–1420

11. Ealick SE (2000) Advances in multiple wavelength anomalous diffraction crystallography. Curr Opin Chem Biol 4:495–499

12. Taylor GL (2010) Introduction to phasing. Acta Cryst Sect D Biol Crystallogr 66:325–338

13. Scapin G (2013) Molecular replacement then and now. Acta Cryst Sect D Biol Crystallogr 69:2266–2275

14. Pike ACW, Garman EF, Krojer T, von Deft F, Carpenter EP (2016) An overview of heavy-atom derivatization of protein crystals. Acta Cryst Sect D Struct Biol 72:303–318

15. Djinović Carugo K, Helliwell JR, Stuhrmann H, Weiss MS (2005) Softer and soft X-rays in macromolecular crystallography. J Synchrotron Rad 12:410–419

16. Durbin SD, Feher G (1996) Protein crystallization. Annu Rev Phys Chem 47:171–20

17. McPherson A (2009) Introduction to Macromolecular Crystallography. Wiley, Hoboken

18. Nanev Christo N (2006) Protein crystal nucleation: recent notions. Cryst Res Technol 42:4–12

19. Milchev, A (2016) Nucleation phenomena in electrochemical systems: kinetic models, ChemTexts 2:4

20. McPherson A (2004) Introduction to protein crystallization. Methods 34:254–265

21. Asherie N (2004) Protein crystallization and phase diagrams. Methods 34:266–272

22. Rupp B (2015) Origin and use of crystallization phase diagrams. Acta Cryst Sect F Struct Biol Commun 71:247–260

23. Bergfors T (2009) Protein Crystallization. International University Line, La Jolla

24. Lu J, Wang XJ, Ching CB (2002) Batch crystallization of soluble proteins: effect of precipitant, temperature and additive. Prog Cryst Growth Charact Mater 45:201–217

25. Thomas DH, Rob A, Rice DW (1989) A novel dialysis procedure for the crystallization of proteins. Protein Eng 2:489–491

26. Salemme FR (1972) A free interface diffusion technique for the crystallization of proteins for x-ray crystallography. Arch Biochem Biophys 151:533–539

27. Rhodes G (2006) Crystallography made crystal clear: a guide for users of macromolecular models. Elsevier, Burlington

28. Collins KD (2004) Ions from the Hofmeister series and osmolytes: effects on proteins in solution and in the crystallization process. Methods 34:300–311

29. Privé GG (2007) Detergents for the stabilization and crystallization of membrane proteins. Methods 41:388–397

30. Trakhanov Sergei, Quiocho Florante A. (2008) Influence of divalent cations in protein crystallization. Protein Science 4:1914–1919

31. McPherson A (1999) Crystallization of Biological Macromolecules. Cold Spring Harbor Laboratory Press, Cold Spring Harbor

32. Pope MT (1983) Heteropoly and isopoly oxometalates. Springer-Verlag, Berlin

33. Wang SS, Yang GY (2015) Recent advances in polyoxometalate-catalyzed reactions. Chem Rev 115:4893–4962

34. Song YF, Tsunashima R (2012) Recent advances on polyoxometalate-based molecular and composite materials. Chem Soc Rev 41:7384–7402

35. Rhule JT, Hill CL, Judd DA, Schinazi RF (1998) Polyoxometalates in medicine. Chem Rev 98:327–358

36. Yamase T (2005) Anti-tumor, -viral, and -bacterial activities of polyoxometalates for realizing an inorganic drug. J Mater Chem 15:4773–4782

37. Hasenknopf B (2005) Polyoxometalates: introduction to a class of inorganic compounds and their biomedical applications. Front Biosci 10:275–287

38. Bijelic A, Aureliano M, Rompel A (2018) The antibacterial activity of polyoxometalates: structures, antibiotic effects and future perspectives. Chem Commun 54:1153-1169

39. a) Bijelic A, Aureliano M, Rompel A (2018) Polyoxometalates as potential next-generation metallodrugs in the combat against cancer. Angew Chem Int Ed in press doi: 10.1002/anie.201803868
b) Bijelic A, Aureliano M, Rompel A (2018) Im Kampf gegen Krebs: Polyoxometallate als nächste Generation metallhaltiger Medikamente. Angew Chem in press doi: 10.1002/ange.201803868

40. Bijelic A, Rompel A (2015) The use of polyoxometalates in protein crystallography - An attempt to widen a well-known bottleneck. Coord Chem Rev 299:22–38

41. Bijelic A, Rompel A (2017) Ten good reasons for the use of the tellurium-centered Anderson–Evans polyoxotungstate in protein crystallography. Acc Chem Res 50:1441–1448

42. Hervé G, Tézé A, Contant R (2003) In: Borras-Alemar JJ, Coronado E, Müller A, Pope MT (ed) Polyoxometalate Molecular Science (1st edn). Springer-Verlag, Dordrecht

43. Pope MT (2007) In: Lippard SJ (ed) Progress in Inorganic Chemistry (volume 39). Wiley, New York

44. Berzelius JJ (1826) Beitrag zur näheren Kenntnis des Molybdäns. Ann Phys 82:369-392

45. Keggin JF (1933) Structure of the crystals of 12-phosphotungstic acid. Nature 132:351

46. Anderson JS (1937) Constitution of the poly-acids. Nature 140:850

47. Lunk H-J, Hartl H (2017) Discovery, properties and applications of molybdenum and its compounds. ChemTexts 3:13

48. Evans HT (1948) The crystal structures of ammonium and potassium molybdotellurates. J Am Chem Soc 70:1291–1292

49. Wells AF (1940) X. Finite complexes in crystals: a classification and review. Lond Edinb Dubl Phil Mag 30:103–134

50. Dawson B (1953) The structure of the 9(18)-heteropoly anion in potassium 9(18)-tungstophosphate, $K_6(P_2W_{18}O_{62})\cdot 14H_2O$. Acta Cryst 6:113–126

51. Lindqvist I (1952) On the structure of the paratungstate ion. Acta Cryst 5:667-670

52. Lipscomb WN (1965) Paratungstate ion. Inorg Chem 4:132-134

53. Lunk HJ, Čuvaev VF, Kolli ID, Spicyn VI (1968) Untersuchung der Struktur von Lithium-, Natrium- und Kalium-Parawolframat mittels ^{1}H-NMR (Russisch). Doklady Akad Nauk SSSR 181:357-360

54. Weiss G (1969) Die Struktur des Parawolframations am Beispiel des Ammoniumparawolframates $(NH_4)_{10}[H_2W_{12}O_{42}]\cdot 10H_2O$. Z Anorg Allg Chem 368: 279-283

55. Evans, Jr. HT, Prince E (1983) Location of internal hydrogen atoms in the paradodecatungstate polyanion by neuton diffraction. J Am Chem Soc 105: 4838-4839

56. Lindqvist I (1953) The structure of the hexaniobate ion in $7Na_2O\cdot 6Nb_2O_5\cdot 32H_2O$. Ark Kemi 5: 247-250

57. Dauter Z (2005) Use of polynuclear metal clusters in protein crystallography. Comptes Rendus Chimie 8:1808–1814

58. Felts RL, Reilly TJ, Tanner JJ (2006) Structure of Francisella tularensis AcpA: prototype of a unique superfamily of acid phosphatases and phospholipases C. J Biol Chem 281:30289–30298

59. Arvai AS, Bourne Y, Hickey MJ, Tainer JA (1995) Crystal structure of the human cell cycle protein CksHs1: single domain fold with similarity to kinase N-lobe domain. J Mol Biol 249:835–842

60. Davies DR, Hol WG (2004) The power of vanadate in crystallographic investigations of phosphoryl transfer enzymes. FEBS Lett 577:315–321

61. Crans DC, Smee JJ, Gaidamauskas E, Yang L (2004) The chemistry and biochemistry of vanadium and the biological activities exerted by vanadium compounds. Chem Rev 104:849–902

62. Tocilj A, Schlünzen F, Janell D, Glühmann M, Hansen HA, Harms J, Bashan A, Bartels H, Agmon I, Franceschi F, Yonath A (1999) The small ribosomal subunit from *Thermus thermophilus* at 4.5 Å resolution: pattern fittings and the identification of a functional site. Proc Natl Acad Sci USA 96:14252–14257

63. Schluenzen F, Tocilj A, Zarivach R, Harms J, Glühmann M, Janell D, Bashan A, Bartels H, Agmon I, Franceschi F, Yonath A (2000) Structure of functionally activated small ribosomal subunit at 3.3 angstroms resolution. Cell 102:615–623

64. Pioletti M, Schlünzen F, Harms J, Zarivach R, Glühmann M, Avila H, Bashan A, Bartels H, Auerbach T, Jacobi C, Hartsch T, Yonath A, Franceschi F (2001) Crystal structures of complexes of the small ribosomal subunit with tetracycline, edeine and IF3. EMBO J 20:1829–1839

65. Rudenko G, Henry L, Henderson K, Ichtchenko K, Brown MS, Goldstein JL, Deisenhofer J (2002) Structure of the LDL receptor extracellular domain at endosomal pH. Science 298:2353–2358

66. Li Y, He Y, Luo Y (2009) Crystal structure of an archaeal Rad51 homologue in complex with a metatungstate inhibitor. Biochemistry 48:6805–6810

67. Zebisch M, Krauss M, Schäfer P, Sträter N (2012) Crystallographic evidence for a domain motion in rat nucleoside triphosphate diphosphohydrolase (NTPDase) 1. J Mol Biol 415:288–306

68. Zebisch M, Krauss M, Schäfer P, Sträter N (2014) Structures of *Legionella pneumophila* NTPDase1 in complex with polyoxometallates. Acta Cryst Sect D Biol Crystallogr 70:1147–1154

69. Karakas E, Furukawa H (2014) Crystal structure of a heterotetrameric NMDA receptor ion channel. Science 344:992–997

70. Mauracher SG, Molitor C, Al-Oweini R, Kortz U, Rompel A (2014) Latent and active abPPO4 mushroom tyrosinase cocrystallized with hexatungstotellurate(VI) in a single crystal. Acta Cryst Sect D Biol Crystallogr 70:2301–2315

71. Bijelic A, Molitor C, Mauracher SG, Al-Oweini R, Kortz u, Rompel A (2015) Hen egg-white lysozyme crystallisation: protein stacking and structure stability enhanced by a tellurium(VI)-centered polyoxotungstate. ChemBioChem 16:233–241

72. Molitor C, Bijelic A, Rompel A (2016) *In situ* formation of the first proteinogenically functionalized $[TeW_6O_{24}O_2(Glu)]^{7-}$ structure reveals unprecedented chemical and geometrical features of the Anderson-type cluster. Chem Commun 52:12286–12289

73. Sap A, De Zitter E, Van Meervelt L, Parac-Vogt TN (2015) Structural characterization of the complex between hen egg-White lysozyme and Zr(IV)-substituted Keggin polyoxometalate as artificial protease. Chem. Eur. J. 21:11692–11695

74. Nilius B, Prenen J, Janssens A, Voets T, Droogmans G (2004) Decavanadate modulates gating of TRPM4 cation channels. J Physiol 560:753–765

75. Ren Y, Schmiege P, Blobel G (2017) Structural and biochemical analyses of the DEAD-box ATPase Sub2 in association with THO or Yra1. Elife 6:e20070

76. Mac Sweeney A, Chambovey A, Wicki M, Müller M, Artico N, Lange R, Bijelic A, Breibeck J, Rompel A (2018) The crystallization additive tellurium-centered Anderson−Evans polyoxotungstate alters the

crystallization behavior of the HSP70 nucleotide binding domain and is bound in two different crystal structures. PLoS ONE 13: e0199639

77. Bond CS, White MF, Hunter WN (2002) Mechanistic implications for *Escherichia coli* cofactor- dependent phosphoglycerate mutase based on the high-resolution crystal structure of a vanadate complex. J Mol Biol 316:1071–1081

78. Locher KP, Lee AT, Rees DC (2002) The *E. coli* BtuCD structure: a framework for ABC transporter architecture and mechanism. Science 296:1091–1098

79. Evans HR, Holloway DE, Sutton JM, Ayriss J, Shone CC, Acharya KR (2004) C3 exoenzyme from *Clostridium botulinum*: structure of a tetragonal crystal form and a reassessment of NAD-induced flexure. Acta Cryst Sec D Biol Crystallogr 60:1502–1505

80. Caradoc-Davies TT, Cutfield SM, Lamont IL, Cutfield JF (2004) Crystal structures of *Escherichia coli* uridine phosphorylase in two native and three complexed forms reveal basis of substrate specificity, induced conformational changes and influence of potassium. J Mol Biol 337:337–354

81. Nowotny M, Yang W (2006) Stepwise analyses of metal ions in RNase H catalysis from substrate destabilization to product release. EMBO J 25:1924–1933

82. Almo SC, Bonanno JB, Sauder JM, Emtage S, Dilorenzo TP, Malashkevich V, Wasserman SR, Swaminathan S, Eswaramoorthy S, Agarwal R, Kumaran D, Madegowda M, Ragumani S, Patskovsky Y, Alvarado J, Ramagopal UA, Faber-Barata J, Chance MR, Sali A, Fiser A, Zhang ZY, Lawrence DS, Burley SK (2007) Structural genomics of protein phosphatases. J Struct Funct Genomics 8:121–140

83. Reinelt S, Hofmann E, Gerharz T, Bott M, Madden DR (2003) The structure of the periplasmic ligand-binding domain of the sensor kinase CitA reveals the first extracellular PAS domain. J Biol Chem 278:39189–39196

84. Schemberg J, Schneider K, Demmer U, Warkentin E, Müller A, Ermler U (2007) Towards biological supramolecular chemistry: a variety of pocket-templated, individual metal oxide cluster nucleations in the cavity of a Mo/W-storage protein. Angew Chem Int Ed 46:2408–2413

85. Kowalewski B, Poppe J, Demmer U, Warkentin E, Dierks T, Ermler U, Schneider K (2012) Nature’s polyoxometalate chemistry: X-ray structure of the Mo storage protein loaded with discrete polynuclear Mo-O clusters. J Am Chem Soc 134:9768–9774

86. Zebisch M, Krauss M, Schäfer P, Lauble P, Sträter N (2013) Crystallographic Snapshots along the reaction pathway of nucleoside triphosphate diphosphohydrolases. Structure 21:1460–1475

87. Poppe J, Warkentin E, Demmer U, Kowalewski B, Dierks T, Schneider K, Ermler U (2014) Structural diversity of polyoxomolybdate clusters along the three-fold axis of the molybdenum storage protein. J Inorg Biochem 138:122–128

88. Weinert T, Olieric N, Cheng R, Brünle S, James D, Ozerov D, Gashi D, Vera L, Marsh M, Jaeger K, Dworkowski F, Panepucci E, Basu S, Skopintsev P, Dore AS, Geng T, Cooke RM, Liang M, Prota AE, Panneels V, Nogly P, Ermler U, Schertler G, Hennig M, Steinmetz MO, Wang M, Standfuss J (2017) Serial millisecond crystallography for routine room-temperature structure determination at synchrotrons. Nature Comm 8:542

89. Bae JH, Lew ED, Yuzawa S, Tome F, Lax I, Schlessinger J (2009) The selectivity of receptor tyrosine kinase signaling is controlled by a secondary SH2 domain binding site. Cell 138:514–524

90. Seixas JD, Mukhopadhyay A, Santos-Silva T, Otterbein LE, Gallo DJ, Rodrigues SS, Guerreiro BH, Goncalves AM, Penacho N, Marques AR, Coelho AC, Reis PM, Romao MJ, Romao CC (2013) Characterization of a versatile organometallic pro-drug (CORM) for experimental CO based therapeutics. Dalton Trans 42:5985–5998

91. Yu X, Yang G, Yan C, Baylon JL, Jiang J, Fan H, Lu G, Hasegawa K, Okumura H, Wang T, Tajkhorshid E, Li S, Yan N (2017) Dimeric structure of the uracil:proton symporter UraA provides mechanistic insights into the SLC4/23/26 transporters. Cell Res 27:1020–1033

92. Zhang G, Keita B, Craescu CT, Miron S, de Oliveira P, Nadjo L (2007) Polyoxometalate binding to human serum albumin: a thermodynamic and spectroscopic approach. J Phys Chem B 111:11253–11259

93. Zhang G, Keita B, Brochon JC, de Oliveira P, Nadjo L, Craescu CT, Miron S (2007) Molecular interaction and energy transfer between human serum albumin and polyoxometalates. J Phys Chem B 111:1809–1814

94. Zhang G, Keita B, Craescu CT, Miron S, de Oliveira, Nadjo L (2008) Molecular interactions between Wells-Dawson type polyoxometalates and human serum albumin. Biomacromolecules 9:812–817

95. Hungerford G, Hussain F, Patzke GR, Green M (2010) The photophysics of europium and terbium polyoxometalates and their interaction with serum albumin: a time-resolved luminescence study. Phys Chem Chem Phys 12:7266–7275

96. Goovaerts V, Stroobants K, Absillis G, Parac-Vogt TN (2013) Molecular interactions between serum albumin proteins and Keggin type polyoxometalates studied using luminescence spectroscopy. PhysChem ChemPhys 15:18378–18387

97. Arefian M, Mirzaei M, Eshtiagh-Hosseini H, Frontera A (2017) A survey of the different roles of polyoxometalates in their interaction with amino acids, peptides and proteins. Dalton Trans 46:6812–6829

98. Nabika H, Inomata Y, Itoh E, Unoura K (2013) Activity of Keggin and Dawson polyoxometalates toward model cell membrane. RSC Advances 3:21271–21274

99. Jing B, Hutin M, Connor E, Cronin L, Zhu Y (2013) Polyoxometalate macroion induced phase and morphology instability of lipid membrane. Chemical Science 4:3818–3826

100. Nabika H, Sakamoto A, Motegi T, Tero R, Yamaguchi D, Unoura K (2016) Imaging characterization of cluster-induced morphological changes of a model cell membrane. J Phys Chem C 120:15640–15647

101. Kobayashi D, Nakahara H, Shibata O, Unoura K, Nabika H (2017) Interplay of hydrophobic and electrostatic interactions between polyoxometalates and lipid molecules. J Phys Chem C 121:12895–12902

102. Molitor C, Mauracher SG, Rompel A (2016) Aurone synthase is a catechol oxidase with hydroxylase activity and provides insights into the mechanism of plant polyphenol oxidases. Proc Natl Acad Sci USA 113:1806-1815

103. Boggon TJ, Shapiro L (2000) Screening for phasing atoms in protein crystallography. Structure 8:143-149

104. Thygesen J, Weinstein S, Franceschi F, Yonath A (1996) The suitability of multi-metal clusters for phasing in crystallography of large macromolecular assemblies. Structure 4:513–518

105. Ladenstein R, Schneider M, Huber R, Bartunik HD, Wilson K, Schott K, Bacher A (1988) Heavy riboflavin synthase from *Bacillus subtilis.* Crystal structure analysis of the icosahedral beta 60 capsid at 3.3 Å resolution. J Mol Biol 203:1045–1070

106. Weaver TM, Levitt DG, Donnelly MI, Stevens PP, Banaszak LJ (1995) The multisubunit active site of fumarase C from *Escherichia coli.* Nat Struct Biol 2:654–662

107. Löwe J, Stock D, Jap B, Zwickl P, Baumeister W, Huber R (1995) Crystal structure of the 20S proteasome from the archaeon *T. acidophilum* at 3.4 Å resolution. Science 268:533–539

108. Ban N, Nissen P, Hansen J, Capel M, Moore PB, Steitz TA (1999) Placement of protein and RNA structures into a 5 Å-resolution map of the 50S ribosomal subunit. Nature 400:841–847

109. Fu J, Gnatt AL, Bushnell DA, Jensen GJ, Thompson NE, Burgess RR, David PR, Kornberg RD (1999) Yeast RNA polymerase II at 5 Å resolution. Cell 98:799–810

110. Rudenko G, Henry L, Vonrhein C, Bricogne G, Deisenhofer (2003) ’MAD’ly phasing the extracellular domain of the LDL receptor: a medium-sized protein, large tungsten clusters and multiple non-isomorphous crystals. Acta Cryst Sect D Biol Crystallogr 59:1978–1986

111. Naskar B, Diat O, Nardello-Rataj V, Bauduin P (2015) Nanometer-size polyoxometalate anions adsorb strongly on neutral soft surfaces. J Phys Chem C 119:20985–20992

112. Solé-Daura A, Goovaerts V, Stroobants K, Absillis G, Jimenez-Lozano P, Poblet JM, Hirst JD, Parac-Vogt TN, Carbo JJ (2016) Probing polyoxometalate–protein interactions using molecular dynamics simulations. Chem Eur J 22:15280–15289

113. Ladenstein R, Bacher A, Huber R (1987) Some observations of a correlation between the symmetry of large heavy-atom complexes and their binding sites on proteins. J Mol Biol 195:751–753

114. Hollas JM (1972) Symmetry in molecules. Chapman and Hall, London.

115. Mauracher SG, Molitor C, Al-Oweini R, Kortz U, Rompel A (2014) Crystallization and preliminary X-ray crystallographic analysis of latent isoform PPO4 mushroom (*Agaricus bisporus*) tyrosinase. Acta Cryst Sect F Struct Biol Cryst Commun 70:263–266

116. Schmidt KJ, Schrobilgen GJ, Sawyer JF (1986) Hexasodium hexatungstotellurate(VI) 22-hydrate. Acta Cryst Sect C Cryst Struct Commun 42:1115–1118

117. Lorenzo-Luis PA, Gili P, Sánchez A, Rodriguez-Castellón E, Jiménez-Jiménez J, Ruiz-Pérez C, Solans X (1999) Tungstotellurates of the imidazolium and 4-methylimidazolium cations. Transit Metal Chem 24:686–692

118. Blazevic A, Al-Sayed E, Roller A, Giester G, Rompel A (2015) Tris-functionalized hybrid Anderson polyoxometalates: synthesis, characterization, hydrolytic stability and inversion of protein surface charge. Chem Eur J 21:4762–4771

119. Al-Sayed E, Blazevic A, Roller A, Rompel A (2015) The synthesis and characterization of aromatic hybrid Anderson–Evans POMs and their serum albumin interactions: the shift from polar to hydrophobic interactions. Chem Eur J 21:17800–17807

120. Blazevic A, Rompel A (2016) The Anderson–Evans polyoxometalate: From inorganic building blocks via hybrid organic–inorganic structures to tomorrows “Bio-POM”. Coord Chem Rev 307:42–64

121. Gumerova NI, Roller A, Rompel A (2016) Synthesis and characterization of the first nickel(II)-centered single-side tris-functionalized Anderson-type polyoxomolybdate. Eur J Inorg Chem 2016:5507–5511

122. Gumerova NI, Roller A, Rompel A (2016) $[Ni(OH)W_6O_{18}(OCH_2)_3CCH_2OH]^{4-}$: the first tris-functionalized Anderson-type heteropolytungstate. Chem Commun 52:9263–9266

123. Nikitina, EA (1962) Heteropopolyverbindungen (in Russisch). Staatlicher wissenschaftlich-technischer Verlag für chemische Literatur, Moskau

124. Boyd T, Mitchell SG, Gabb D, Long DL, Cronin L (2011) Investigating cation binding in the polyoxometalate-super-Crown $[P_8W_{48}O_{184}]^{40-}$. Chem Eur J 17:12010–12014

125. Stroobants K, Moelants E, Ly HGT, Proost P, Bartik K, Parac-Vogt TN (2013) Polyoxometalates as a novel class of artificial proteases: selective hydrolysis of lysozyme under physiological pH and temperature promoted by a cerium(IV) Keggin-type polyoxometalate. Chem Eur J 19:2848–2858

126. Ly HGT, Parac-Vogt TN (2017) Spectroscopic study of the interaction between horse heart myoglobin and zirconium(IV)-substituted polyoxometalates as artificial proteases. ChemPhysChem 18:2451–2458

127. Yang L, Zhou Z, Ma PT, Zhang XF, Wang JP, Niu JY (2013) Three organic–inorganic hybrid B-Anderson polyoxoanions as building blocks: syntheses, structures, and characterization of $[(C_6H_5NO_2)_2Ln(H_2O)_6](CrMo_6O_{24}H_6)\cdot 2C_6H_5NO_2\cdot 6H_2O$ (Ln = Sm, Dy, Er). J Coord Chem 66:1058–1067

128. Gao Q, Li F, Wang Y, Xu L, Bai J, Wang Y (2014) Organic functionalization of polyoxometalate in aqueous solution: self-assembly of a new building block of $\{VMo_6O_{25}\}$ with triethanolamine. Dalton Trans 43:941–944

129. Molitor C, Mauracher SG, Rompel A (2015) Crystallization and preliminary crystallographic analysis of latent, active and recombinantly expressed aurone synthase, a polyphenol oxidase, from *Coreopsis grandiflora*. Acta Cryst Sect F Struct Biol Cryst Commun 71:746–751

130. Molitor C, Bijelic A, Rompel A (2017) The potential of hexatungstotellurate(VI) to induce a significant entropic gain during protein crystallization. IUCrJ 4:734–740

131. Hašek J (2011) Protein surface shielding agents in protein crystallization. J Synchrotron Rad 18:50–52

132. Pretzler M, Bijelic A, Rompel A (2017) Heterologous expression and characterization of functional mushroom tyrosinase (*Ab*PPO4). Sci Rep 7:1810

133. Derewenda ZS, Vekilov PG (2006) Entropy and surface engineering in protein crystallization. Acta Cryst Sect D Biol Crystallogr 62:116–124

134. Crans DC (1994) Aqueous chemistry of labile oxovanadates: relevance to biological studies. Comments Inorg Chem 16:1–33
